# Microglia Pyroptosis-Derived IL-18 Drives White Matter Injury in Developing Brain following Hypothermic Hypoxia-Ischemia

**DOI:** 10.1007/s12264-026-01602-9

**Published:** 2026-03-09

**Authors:** Hongtong Chen, Shengyu Jin, Mingdong Liu, Yifan Zhu, Liren Zhang, Cong Li, Peng Liu, Xiaoping Tong, Zhongqun Zhu

**Affiliations:** 1 Department of Cardiothoracic Surgery, Shanghai Children’s Medical Center, Shanghai Jiao Tong University School of Medicine, Shanghai, 200127 China; 2 Department of Obstetrics and Gynecology, Songjiang Research Institute, Shanghai Key Laboratory of Emotions and Affective Disorders, Songjiang Hospital Affiliated to Shanghai Jiao Tong University School of Medicine, Shanghai, 201620 China; 3 Department of Anatomy and Physiology, School of Basic Medical Sciences, Shanghai Jiao Tong University School of Medicine, Shanghai, 201318 China; 4 Key Laboratory of Molecular Neurobiology, Ministry of Education, Navy Military Medical University, Shanghai, 200433 China; 5 Guangzhou Women and Children’s Medical Center, Guangdong Provincial Clinical Research Center for Child Health, Guangzhou Medical University, Guangzhou, 510623 China; 6 Department of Neurology, Changhai Hospital, Naval Medical University, Shanghai, 200433 China

**Keywords:** Microglia, Pyroptosis, White matter injury, Hypothermic hypoxia-ischemia

## Abstract

Hypothermia is widely acknowledged to exert protective effects against cerebral hypoxic-ischemic injury. Despite the neuroprotective effects of hypothermia, the developing brain remains vulnerable to white matter injury (WMI) during hypothermic hypoxia-ischemia, potentially disrupting neurodevelopment and leading to long-term neurological deficits. However, the mechanisms underlying WMI and effective therapeutic strategies following hypothermic hypoxia-ischemia in the developing brain are not well understood. Our study demonstrates that microglia experience pyroptosis following hypothermic hypoxia-ischemia. The release of interleukin 18 (IL-18) derived from pyroptotic microglia induces mature oligodendrocyte death and axonal demyelination, resulting in WMI. Pharmacological inhibition of pyroptosis with disulfiram (DSF) significantly alleviates WMI *in vitro* and *in vivo*. These findings highlight microglia pyroptosis as a potential therapeutic target to prevent neurodevelopmental impairment in the developing brain following hypothermic hypoxia-ischemia.

**Supplementary Information:**

The online version contains supplementary material available at 10.1007/s12264-026-01602-9.

## Introduction

Hypothermia is recognized as a crucial neuroprotective strategy against cerebral hypoxia-ischemia in clinical practice. As a surgical technique centered on hypothermic protection, deep hypothermic circulatory arrest (DHCA) is a widely used technique in cardiac and macrovascular surgery, particularly for complex congenital heart disease [1, 2]. It reduces intraoperative blood loss and lowers the patient’s core body temperature (typically to 18℃) to provide cerebral protection, while also decreasing oxygen consumption and metabolic rate in peripheral organs to prevent ischemic injury [3, 4]. However, despite the neuroprotective effects of hypothermia, DHCA can lead to neurodevelopmental impairments in the developing brain, such as poor motor skills, delayed language development, and cognitive disorders [5]. MRI imaging has shown that the developing brain may experience white matter injury (WMI) following DHCA [6, 7]. This suggests that hypothermia still fails to fully prevent residual cerebral hypoxic-ischemic injury.

White matter is an essential component of the central nervous system, playing a critical role in the transmission of neural signals across different brain regions [8]. It is integral to higher cognitive functions, including memory, attention, behavior, and language processing [9–11]. Among the various risk factors associated with WMI, hypoxia-ischemia, infections, and the inherent vulnerability of immature white matter are particularly significant [12–16]. These factors can disrupt the normal development and functioning of white matter, leading to long-term neurological impairments.

DHCA causes cerebral hypothermic hypoxia-ischemia, making it a suitable model for studying WMI following hypothermic hypoxia-ischemia. Studies in adult rat DHCA models have shown cognitive dysfunction driven by microglia activation, which triggers inflammatory factor release and neuronal damage [17]. Besides, in the neonatal rat brain slice model simulating hypothermic circulatory arrest, microglia activation leads to inflammatory factor release and demyelination [18]. These findings establish microglia as central mediators in both gray and white matter injury due to hypothermic hypoxia-ischemia. However, the specific molecular mechanisms by which microglia activation causes WMI in the developing brain following hypothermic hypoxia-ischemia remain unclear.

Recent advances have highlighted microglial pyroptosis as a novel mechanism inducing brain injury. This gasdermin D (GSDMD)-dependent programmed cell death [19] process causes inflammatory pore formation, cytokine release, and tissue damage [20, 21]. In lysolecithin (LPC)-induced demyelination, microglia pyroptosis exacerbates inflammation and myelin loss *via* the NF-κB/ERK1/2 pathway [22]. These studies consider microglia pyroptosis as an emerging therapeutic target for brain protection.

Our prior research has shown that hypothermic hypoxia-ischemia activates microglia, leading to the release of inflammatory cytokines and subsequent demyelination [18]. Building on this, we developed hypothermic oxygen-glucose deprivation (OGD) and weanling rat DHCA models. Our findings indicate that hypoxia-ischemia induces gasdermin D (GSDMD)-dependent pyroptosis in microglia under hypothermia, resulting in the release of interleukin-18 (IL-18). This process drives oligodendrocyte death and axonal demyelination. Disulfiram-mediated pharmacological inhibition of pyroptosis significantly alleviated WMI in the developing brain in vitro (OGD) and in vivo (DHCA). Thus, our study underscores that hypothermic hypoxic-ischemic injury, primarily mediated by microglia pyroptosis, persists under hypothermic conditions. This finding suggests that targeting microglia pyroptosis could be a promising therapeutic approach for WMI in the developing brain following hypothermic hypoxia-ischemia.

## Materials and Methods

### Animals and Perfusion System

The cerebral hypoxia-ischemia was simulated *in vitro* using a perfusion system, following previously published methods [18]. We used 3-week-old Sprague-Dawley rats (Shanghai Children’s Medical Center, Shanghai), as their brains are in the developmental stage [23, 24]. This study was approved by the Institutional Animal Care and Use Committee of Shanghai Children’s Medical Center, Shanghai Jiao Tong University School of Medicine.

Rats were placed on ice, rapidly decapitated, and their brains were immediately immersed in ice-cold artificial slicing solution containing (mmol/L): 125 NaCl, 2.5 KCl, 1.25 NaH_2_PO_4_, 25 NaHCO_3_, 25 Glucose, 75 Sucrose, 1 MgCl_2_, 2 CaCl_2_, equilibrated with 95% O_2_/5% CO_2_, pH 7.4. Brain slices (400 μm) were prepared using a vibratome (VT1000S, Leica Microsystems, Mannheim, Germany). Only slices containing the corpus callosum—the primary white matter structure in the rat brain—were selected for perfusion experiments. The slices were incubated in oxygenated artificial cerebrospinal fluid (aCSF) at 31℃ for at least 1 h before further experimentation. Brain slices were transferred to the perfusion system and subjected to two distinct perfusion protocols: (1) Oxygenated glucose-aCSF (mmol/L): 125 NaCl, 2.5 KCl, 1.25 NaH_2_PO_4_, 25 NaHCO_3_, 10 Glucose, 1 MgCl_2_, 2 CaCl_2_, equilibrated with 95% O_2_/5% CO_2_, pH 7.4. (2) Hypoxic sucrose-aCSF (mmol/L): 125 NaCl, 2.5 KCl, 1.25 NaH_2_PO_4_, 25 NaHCO_3_, 10 Sucrose, 1 MgCl_2_, 2 CaCl_2_, equilibrated with 95% N_2_/5% CO_2_, pH 7.4. The temperature was maintained constant throughout all experimental procedures.

### Oxygen-Glucose Deprivation

Oxygen-glucose deprivation (OGD) was used to simulate hypoxia-ischemia. This was achieved by hypoxic sucrose-aCSF, as described previously. After OGD, the brain slices were reperfused with oxygenated glucose-aCSF and equilibrated with 95% O_2_/5% CO_2_.

### Perfusion Procedures

Three hypothermic protection strategies employed in clinical implementation: normothermia (31℃), moderate hypothermia (25℃), and deep hypothermia (18℃). Building on this, OGD was conducted under the following conditions: (1) 31℃ sham group; (2) OGD at 18℃, 25℃, and 31℃. Before OGD, the perfusion system was cooled to target temperatures for 5 min, followed by 60 min of OGD. Then the brain slices underwent 10 min of reperfusion with oxygenated glucose-aCSF, subsequently rewarmed to 31℃ for 30 min.

### Weanling Rats DHCA Model

Male Sprague-Dawley rats (3 weeks old) were maintained under standard conditions with access to food/water. Following anesthesia induction with 2% isoflurane (RWD Life Science, R510-22-10, Shenzhen, China), tracheal intubation was performed using an 18-gauge cannula (Xi’an Xijing Medical Appliance, Xian, China) connected to a ventilator (RWD Instruments, Shenzhen, China). ECG electrodes (Philips Intellivue mp2, Amstelplein, Netherlands) were positioned on three limbs before triple disinfection of the chest with iodophor (60005-1, Yuyan Biology, Shanghai, China). A scalpel incision extended from the chest to the xiphoid process, with immediate hemostasis achieved using an electrosurgical unit (Huihan Technology, Suzhou, China) while avoiding major vessels. After thoracic cavity exposure *via* thymus excision (electrocautery) and sternal retractor placement (Tigergene Technology, Nanjing, China), the right common carotid artery (RCCA) was blunt-dissected (mosquito forceps FS027, Beyotime, Shanghai, China) and looped proximally/distally with 2-0 sutures. A 22-gauge catheter was advanced into the RCCA to the ascending aorta and secured with sutures. Similarly, the superior vena cava (SVC) was dissected, incised proximally, and cannulated with a custom venous catheter advanced to the right atrium. Heparin (500 U/kg, 1 mL) was administered pre-cannulation. Post-cannulation stability was confirmed by normal ECG and vital signs. The circuit connected to the RCCA catheter (pump flow: 1–2 mL/min) and the SVC catheter-reservoir. Initial water bath temperature was set at 31℃; after heart rate stabilization, pump flow increased to 80–100 mL/kg per min. The water tank temperature was gradually adjusted to 18℃ while continuously monitoring the rats’ rectal temperature. When the rectal temperature reached 18℃, the pump was stopped, and blood from the SVC was drained into the reservoir. Upon cessation of heart rate, drainage was halted, and the circuit was closed. Gradual rewarming was then initiated until a core temperature of 31℃ was restored, after which cardiopulmonary bypass (CPB) was discontinued.

### Method of Disulfiram Administration

For the perfusion system, 10 mmol/L disulfiram was dissolved in DMSO and further diluted to 10 μmol/L in PBS [25]. For DHCA, rats were treated with DSF (50 mg/kg) formulated in corn oil or vehicle by intraperitoneal injection 4 h in advance of DHCA surgery [26].

### Quantitative Real-Time PCR (qPCR)

Total RNA was extracted from frozen tissue specimens (−80℃) using Trizol reagent (R0016, Beyotime, Shanghai, China) following the manufacturer’s protocol. Briefly, tissues were homogenized in 1 mL Trizol, mixed with 200 μL chloroform, and incubated at room temperature for 5 min. After centrifugation (12,000 ×g, 4℃, 15 min), the aqueous phase was collected and combined with an equal volume of isopropanol to precipitate RNA. The RNA pellet was washed twice with 75% ethanol (12,000 ×g, 4℃, 15 min each) and resuspended in 20 μL nuclease-free water. cDNA was synthesized from the isolated RNA. Target genes (*Nlrp3*, *Casp1*, *Gsdmd*, *Mbp*, *Il1β*, and *Il18*) were amplified using KAPA HiFi HotStart ReadyMix (KK2601, KAPA Biosystems, Wilmington, USA) with gene-specific primers (Table S1). *Gapdh* expression was used as the endogenous control for normalization.

### Western Blot

Tissues were homogenized in ice-cold lysis buffer consisting of 50 mmol/L Tris-HCl (pH 7.6), 150 mmol/L NaCl, 1% NP-40, 0.5% sodium deoxycholate, 0.1% SDS, and protease inhibitors. Lysates were centrifuged (12,000 ×g, 15 min, 4℃) to remove debris, and protein concentrations were determined using the BCA assay (Thermo Fisher Scientific, Waltham, USA). For SDS-PAGE, protein extracts were denatured (65℃, 30 min) and resolved in Tris-glycine buffer. Proteins were transferred to PVDF membranes (162-0177, Bio-Rad, Berkeley, USA) and blocked with 3% BSA (BBI Life Sciences, A600332-0100) in TBS-T (20 mmol/L Tris, 150 mmol/L NaCl, 0.1% Tween 20, pH 7.6) for 2 h at room temperature. Primary antibodies were incubated overnight at 4℃ using the following: mouse anti-GSDMD (1:1000, Santa Cruz, Dallas, USA, Cat# sc-393656, RRID: AB_2728694), mouse anti-MBP (1:500, Millipore, Darmstadt, Germany, Cat# MAB382, RRID: AB_94971), rabbit anti-cleaved CASP1 (1:1000, Cell Signaling, Danvers, USA, Cat# 89332, RRID: AB_2923067), rabbit anti-NLRP3 (1:1000, Cell Signaling, Danvers, USA, Cat# 15101, RRID: AB_2722591), rabbit anti-IKBα (1:1000, Abcam, Cambridge, United Kingdom, Cat# ab32518, RRID: AB_733068), rabbit anti-p-IKBα (1:1000, Abcam, Cambridge, United Kingdom, Cat# ab133462, RRID: AB_2801653), rabbit anti-p65 (1:1000, Abcam, Cambridge, United Kingdom, Cat# ab16502, RRID: AB_2224674), rabbit anti-p-p65 (1:1000, Cell Signaling, Danvers, USA, Cat# 3033, RRID: AB_331284), rabbit anti-p-MLKL (1:1000, Abcam, Cambridge, United Kingdom, Cat# ab196436, RRID: AB_2687465), rabbit anti-cleaved CASP3 (1:1000, Cell Signaling, Danvers, USA, Cat# 9661, RRID: AB_2341188) and rabbit anti-β-actin (1:2000, Invitrogen, Waltham, USA, Cat# MA515739, RRID: AB_10979409). All antibodies used are detailed in Table S2. Membranes were washed with TBS-T and incubated with HRP-conjugated secondary antibodies (anti-rabbit CW0103, 1:5000; anti-mouse CW0102, 1:5000, Cwbio, Beijing, China) for 2 h at room temperature. Blots were developed using Clarity™ Western ECL Substrate (Bio-Rad, Berkeley, USA) and imaged on a Tanon Chemiluminescence system. Band intensities were quantified using ImageJ v1.30 (National Institutes of Health, USA).

### Immunofluorescent Staining

Brain tissue sections (400 μm thick) were fixed overnight in 4% paraformaldehyde (PFA) at 4℃, embedded, and coronally sectioned at 35 μm using a Cryo-Vibratome to encompass the corpus callosum. After permeabilization with 0.3% Triton X-100/PBS for 13 min, sections were blocked for 2 h at room temperature with 10% donkey serum (w9030-05, Ruite Biotechnology, Guangzhou, China) in PBS-T (PBS + 0.1% Triton X-100). Primary antibodies were diluted in PBS with 5% donkey serum and incubated at 37℃ for 2 h, followed by overnight incubation at 4℃. Antibodies included: goat anti-Iba-1 (1:500, Wako, Tokyo, Japan, Cat# 011-27991, RRID: AB_2935833), rabbit anti-GSDMD (1:200, Abcam, Cambridge, United Kingdom, Cat# ab209845, RRID: AB_2783550), mouse anti-MBP (1:500, Millipore, Darmstadt, Germany, Cat# MAB382, RRID: AB_94971), rabbit anti-IL-18 (1:200, Abcam, Darmstadt, Germany, Cat# ab191152, RRID: AB_2737346), rabbit anti-S100β (1:500, Abcam, Darmstadt, Germany, Cat# ab52642, RRID: AB_882426), mouse anti-Olig2 (1:500, Millipore, Darmstadt, Germany, Cat# MABN50, RRID: AB_10807410), rabbit anti-GFAP (1:500, Millipore, Darmstadt, Germany, Cat# AB5804, RRID: AB_2109645), mouse anti-CD68 (1:500, Abcam, Cambridge, United Kingdom, Cat# ab955, RRID: AB_307338), mouse anti-Ki67 (1:500, Cell Signaling, Danvers, USA, Cat# 9449, RRID: AB_2797703) and mouse anti-APC (CC-1) (1:500, Millipore, Darmstadt, Germany, Cat# OP80, RRID: AB_2057371). After washing with PBS-T, sections were incubated with biotinylated secondary antibodies for 2 h at 37℃, including Donkey anti-Goat Alexa Fluor 647 (1:500, Invitrogen, Waltham, USA, Cat# A-21447, RRID: AB_2535864), Donkey anti-Rabbit Alexa Fluor 488 (1:500, Invitrogen, Waltham, USA, Cat# A-21206, RRID: AB_2535792), Donkey anti-Mouse Alexa Fluor 488 (1:500, Invitrogen, Waltham, USA, Cat# A-21202, RRID: AB_141607), Donkey anti-Rabbit Alexa Fluor 647 (1:500, Invitrogen, Waltham, USA, Cat# A-31573, RRID: AB_2536183), Donkey anti-Mouse Alexa Fluor 647 (1:500, Invitrogen, Waltham, USA, Cat# A-31571, RRID: AB_162542) and Donkey anti-Mouse Alexa Fluor 568 (1:500, Invitrogen, Waltham, USA, Cat# A10037, RRID: AB_11180865). Brain sections were counterstained with DAPI (1:1000, Cell Signaling, Danvers, USA, 4083S) for 10 min at room temperature before mounting with Fluoromount™ aqueous medium (13800, AQUA-MOUNT, Burlingame, USA). Images were acquired using a Leica TCS SP8 confocal microscope with HC PL APO CS2 objectives (×20/0.75 dry, ×40/1.30 oil, ×63/1.40 oil) and analyzed in ImageJ v1.30.

### Enzyme-Linked Immunosorbent Assay (ELISA)

The aCSF from the perfusion system and the serum samples from DHCA rats were collected. Target protein concentrations were quantified using rat-specific ELISA kits (Amoy Lunchangshuo Biotech, Xiamen, China) according to the manufacturer’s instructions. The ELISA was performed following standard protocols with minor modifications. A 96-well microplate was coated overnight at 4℃ with the target antigen (or capture antibody) diluted in 0.1 mol/L carbonate-bicarbonate buffer (pH 9.6). The plate was then blocked with 5% bovine serum albumin in PBS for 1–2 h at room temperature to prevent nonspecific binding. After blocking, the plate was washed three times with PBS containing 0.05% Tween-20. Serial dilutions of standards and test samples were added to the wells and incubated for 2 h at 37℃. Following incubation, the plate was washed, and a biotinylated detection antibody was added for an additional 1 h at 37℃. After another washing step, streptavidin-horseradish peroxidase conjugate was applied and incubated for 30 min at room temperature. The reaction was developed using 3,3',5,5'-tetramethylbenzidine substrate, and the enzymatic reaction was stopped with 2 mol/L sulfuric acid. Absorbance was measured at 450 nm using a microplate reader, and sample concentrations were determined from a standard curve.

### Hoechst 33342/Propidium Iodide (PI) Staining

Primary microglia underwent double-staining using a Hoechst 33342/PI Apoptosis Assay Kit (BL116A, Biosharp, Hefei, China) per manufacturer instructions. Cells were harvested and washed twice with ice-cold PBS. Following centrifugation at 300 × g for 5 min, the pellet was resuspended in PBS containing 5 μg/mL Hoechst 33342 and 2 μg/mL propidium iodide. The cell suspension was incubated for 15 min at 37℃ in the dark. After staining, cells were immediately analyzed by fluorescence microscopy (Eclipse Ti, Nikon, Tokyo, Japan) using UV excitation (350 nm) for Hoechst 33342 and green excitation (535 nm) for PI detection. Images were acquired on a Leica TCS SP8 confocal microscope with HC PL APO CS2 objectives.

### Annexin V-FITC/Propidium Iodide (PI) Flow Cytometry

The tissue samples were digested into cells and then stained using the Annexin V-FITC Apoptosis Detection Kit (Millipore, Darmstadt, Germany). Briefly, 1×10^6^ cells were harvested, washed twice with ice-cold PBS, and resuspended in 100 μL of 1× binding buffer. Subsequently, cells were stained with 5 μL Annexin V-FITC and 5 μL propidium iodide (PI, 50 μg/mL) for 15 min at room temperature in the dark. Before analysis, 400 μL of 1× binding buffer was added to each sample. Subsequent quantification was performed on a NovoCyte 2040R flow cytometer (ACEA Bioscience, San Diego, USA).

### Primary Microglia Culture

During primary microglia cell culture, the brains of 24-hour-old neonatal rats were dissected, and meninges were removed in Hanks’ Balanced Salt Solution (HBSS). The rats’ tissues were transferred to digestion solution [(trypsin solution, 0.25% (Gibco, Baltimore, USA); Dnase I, 75 U/mL (Worthington, Lakewood, USA)] and incubated for 10 min in the culture incubator at 37℃. Cells were pelleted by centrifugation (1000 × g, 5 min) using a swinging bucket rotor and cultured in Dulbecco’s Modified Eagle Medium (DMEM) (Gibco, Baltimore, USA) supplemented with 10% fetal bovine serum (FBS) in 75 cm^2^ flasks at 37℃ under 5% CO₂. The medium was replaced after 24 h and refreshed twice weekly. Microglia cell purification was performed as previously described [27]. Briefly, on day 21, cells were digested with 0.0625% trypsin-HBSS for 1–2 h at 37℃. The astrocyte-enriched supernatant was removed, and adherent microglia were maintained in DMEM. For maturation, purified microglia were cultured for 3 d before experimentation.

### Transmission Electron Microscopy

Under a dissecting microscope, the corpus callosum was isolated and minced into 1–3 mm^3^ fragments before primary fixation in 2.5% glutaraldehyde (> 3 h). After 0.1 mol/L phosphate buffer washes, specimens underwent secondary fixation with 0.5% osmium tetroxide under agitation (4℃, 3 h), followed by additional phosphate buffer rinses. Tissues were progressively dehydrated through an ethanol series, infiltrated with propylene oxide, and embedded in Spurr’s resin for polymerization (70℃). Ultrathin sections (≈ 70 nm) prepared using UC7 ultramicrotome (Leica, Mannheim, Germany) were contrasted with uranyl acetate and lead citrate before imaging on Talos 120 transmission electron microscope (Thermo Fisher Scientific, Waltham, USA). The corpus callosum was microdissected into 1–3 mm^3^ fragments under a dissecting microscope and fixed in 2.5% glutaraldehyde (> 3 h). After washing with 0.1 mol/L phosphate buffer, samples were post-fixed in 0.5% osmium tetroxide (4℃, 3 h) with agitation, followed by additional buffer rinses. Tissues were dehydrated through a graded ethanol series, cleared in propylene oxide, and embedded in Spurr’s resin for polymerization (70℃). Ultrathin sections (≈ 70 nm) were cut using a UC7 ultramicrotome, stained with uranyl acetate and lead citrate, and imaged on a Talos 120 transmission electron microscope.

### Single-cell Sequencing Analysis

The acquisition of corpus callosum tissue sections was performed as described previously. The corpus callosum tissue was subjected to single-cell RNA-seq at Shanghai Biotechnology Corporation (Seekgene, Beijing, China). Single-cell RNA Seq libraries were prepared using SeekOne® MM Single Cell 3’ library preparation kit (SeekGene). Briefly, an appropriate number of cells were loaded into the flow channel of the SeekOne® MM chip, which had 170,000 microwells and allowed to settle in the microwells by gravity. After removing the unsettled cells, sufficient Cell Barcoded Magnetic Beads (CBBs) were pipetted into the flow channel and also allowed to settle in microwells with the help of a magnetic field. Next, excess CBBs were rinsed out, and cells in the MM chip were lysed to release RNA, which was captured by the CBB in the same microwell. Then all CBBs were collected, and reverse transcription was performed at 37℃ for 30 min to label cDNA with the cell barcode on the beads. Further Exonuclease I treatment was performed to remove unused primer on CBBs. Subsequently, barcoded cDNA on the CBBs was hybridized with a random primer that had reads 2 SeqPrimer sequence on the 5’ end and could extend to form the second strand DNA with cell barcode on the 3’ end. The resulting second-strand DNA was denatured off the CBBs, purified, and amplified in a PCR reaction. The amplified cDNA product was then cleaned to remove unwanted fragments and added to full length sequencing adapter and sample index by indexed PCR. The indexed sequencing libraries were cleaned up with SPRI beads, quantified by quantitative PCR (KK4824, KAPA Biosystems, Wilmington, USA), and then sequenced on Illumina NovaSeq 6000 with PE150 read length or DNBSEQ-T7 platform with PE100 read length. The Seurat R package (version 3.2.0) was used for further inspection and data analysis [28]. The resulting filtered matrix consisted of ~60,000 cells. The matrix was normalized using the NormalizeData function, and variable features were identified using the FindVariableFeatures function with 2000 genes. The ScaleData function was used to center the gene expression. Next, principal component analysis (PCA) was performed, using the RunPCA function, to obtain the top 50 principal components (PCs). Clustering was conducted using the FindNeighbors and FindClusters functions using 20 PCs and a resolution parameter set to 0.3. Differential gene expression analysis was performed using PRESTO (Supplementary File 2). For visualization, the dimensionality of the data sets was reduced by UMAP. Cell populations were matched to cell types based on the expression of known marker genes and previously identified expression signatures [29]. Gene enrichment was analyzed using Metascape. Single-cell RNA-seq data are available in PRJNA1312850.

### Rat Cytokine Array Panel A

Collect corpus callosum tissue from both the sham and OGD groups. Add RIPA lysis buffer, centrifuge (12,000 × g, 15 min, 4℃), and take the supernatant. Sonicate for 20 s. Equilibrate the array membrane at room temperature for 30 min, then wash with 1× Wash Buffer for 5 min. Add 300 μL of diluted sample (containing an equal amount of total protein, recommended ≥ 1 mg/mL) to each membrane, and incubate at room temperature overnight at 4℃. Wash the membrane three times with 1× Wash Buffer, 10 min each time. Add biotin-labeled antibody mixture and incubate at room temperature for 2 h. Wash the membrane three times with 1× Wash Buffer, 10 min each time. Add streptavidin-HRP and incubate at room temperature for 30 min. Wash the membrane three times with 1× Wash Buffer, 10 min each time. Cover the membrane with ECL chemiluminescence reagent and incubate in the dark for 1 min. Then the membranes were visualized using Clarity^TM^ Western ECL Substrate (Bio-Rad, Hercules, USA) and imaged on a Tanon Chemiluminescence system. Equivalence of protein loading was corrected by probing for the standard value. Band densities were quantified using ImageJ v1.30 (National Institutes of Health, USA).

### Magnetic Resonance Imaging (MRI)

MRI was performed at Shanghai Children’s Medical Center using a 3.0T scanner (Siemens) with a 64-channel infant head coil and cardiorespiratory monitoring. Postoperative pediatric patients (Table S3) were scanned. Anatomical imaging included T1-weighted (repetition time [TR], 2400 milliseconds (ms), echo time [TE], 2.22 ms, slice thickness, 0.80 mm, field of view (FOV), 256 mm, matrix, 320 × 320) and T2-weighted (TR, 3200 ms, TE, 563 ms, slice thickness, 0.8 mm, FOV, 256 mm) sequences. Resting-state MRI (rs-fMRI) acquired 420 volumes over 336 s using an echo-planar imaging (EPI) sequence (TR, 800 ms, TE, 37 ms, FOV, 208 mm, matrix, 104 × 104, slice thickness, 2mm, 72 slices, 144 volumes). Subjects maintained relaxed wakefulness with head immobilization. Diffusion tensor imaging (DTI) used a single-shot EPI sequence (TR, 4200 ms, TE, 89 ms, FOV, 240 mm, matrix, 140 × 140, slice thickness, 2mm, motion-probing gradient in 102 diffusion-encoding directions with a diffusion weighting of 3000 s/mm^2^ [b value] and 10 times of non-diffusion weighted image). White matter injury (WMI) was graded according to established criteria for congenital heart disease based on imaging characteristics defined in prior literature [7, 30], assessed by neurologists.

### DTI and Atlas-Based Analysis

Diffusion tensor imaging (DTI) analysis was performed using FSL (www.fmrib.ox.ac.uk/fsl) [31] following established methods [32]. Preprocessing included eddy current correction, linear registration of diffusion-weighted volumes to the non-diffusion volume (b0) [33, 34], and brain masking [35]. The diffusion tensor model was fitted voxelwise to compute fractional anisotropy (FA) maps, where FA quantifies regional water diffusion directionality in white matter and increases with microstructural maturation [36]. The JHU (Johns Hopkins University) atlas was registered to each child’s individual diffusion-weighted imaging (DWI) space using the ANTs toolkit (https://github.com/ANTsX/ANTs) through a multi-step process. First, the individual’s DWI image was linearly registered to their corresponding T2-weighted structural image. Second, this T2 structural image was nonlinearly registered to a pediatric standard template (the UNC-BCP 4D Infant Brain Volumetric Atlas 2 months). Subsequently, the resulting linear and nonlinear transformation fields were combined to compute their composite inverse transformation field. Finally, the JHU-ICBM-labels-2 mm atlas [37] was warped into the individual child’s space using this composite inverse transformation field and interpolated, enabling atlas-based parcellation for extracting fractional anisotropy (FA) metric values from the participant’s FA image.

### Tractography Methodology

Fiber tracking employed DSI Studio using generalized q-sampling imaging (sampling length ratio = 1.25) to reconstruct orientation distribution functions. Quantitative anisotropy (QA) values guided automated tract identification per published protocols [38].

### Ethical Approval

The ethical approval was reviewed and approved by the Institutional Animal Protection and Use Committee of Shanghai Children’s Medical Center, Shanghai Jiao Tong University School of Medicine (SCMC-LAWEC-2022-613). Ethical approval for research involving human participants was granted by Shanghai Children’s Medical Center, Shanghai Jiao Tong University School of Medicine (approval number: SCMCIRB-K2025167-1).

### Statistical Analysis

All statistical analysis was run in GraphPad Prism 9.0. The graphs were created in GraphPad Prism 9.0. Data are presented as means ± SEM, and the error bars represent SEM for each set of data to be compared. All data were first assessed for normality using the Shapiro-Wilk test and for homogeneity of variances using the *F* test. Based on the outcome of these tests, appropriate parametric or non-parametric tests were chosen for group comparisons. Specifically, for comparisons between two groups that followed a normal distribution, an unpaired two-tailed *t* test was used if variances were equal, or Welch’s corrected *t* test was applied if variances were unequal. For data that did not meet the normality assumption, the Mann-Whitney test was used. Statistical comparisons among three or more groups were performed using a one-way analysis of variance (ANOVA). Researchers were blinded to the groups or samples during the experiments. Regions with FA reduction (DHCA vs. CPB: ΔFA < 0) were mapped to the WMI probability area. The statistical methods were not used to pre-determine sample size or to randomize. Statistical significance was set at *P < 0.05, **P < 0.01, ***P < 0.001.

## Results

### White Matter Injury in Pediatric Patients and Weanling Rats following Hypothermic Hypoxia-ischemia

**Fig. 1:**
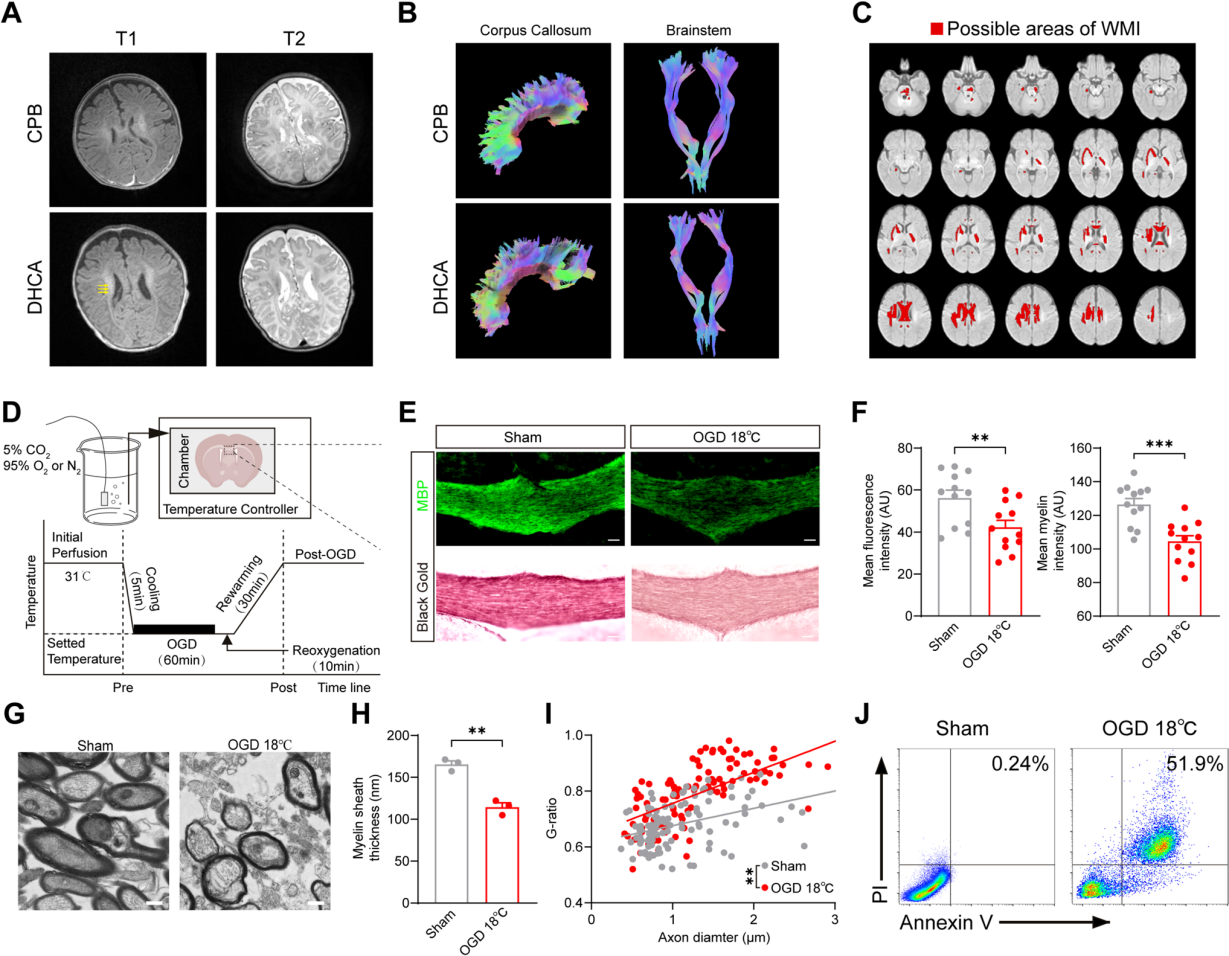
White matter injury in pediatric patients and weanling rats following hypothermic hypoxia-ischemia. **A** Images of the punctate lesions distributed in the bilateral paraventricular white matter. Punctate lesions were observed in the white matter of pediatric patients following DHCA. T1: T1-weighted images, T2: T2-weighted images, CPB: the pediatric patient underwent cardiopulmonary bypass, DHCA: the pediatric patient underwent deep hypothermia circulation arrest. The yellow arrow indicates punctate lesions. **B** Images of the fiber tractography of white matter. A reduction in white matter tracts was observed in pediatric patients following DHCA. **C** Images of the possible areas of WMI. Red areas indicate changes of fractional anisotropy between CPB and DHCA pediatric patient (ΔFA < 0). ΔFA: change in Fractional Anisotropy, CPB vs. DHCA pediatric patient. WMI: White matter injury. **D** Schematic illustration of OGD in brain slices of weanling rats. The brain slices were incubated, then cooled to the target temperature for 5 min, followed by transfer to sucrose-aCSF for 60 min of OGD. After OGD, the slices underwent 10 min of reoxygenation before being rewarmed to 31°C. OGD: oxygen and glucose deprivation. **E, F** Representative images of immunofluorescence staining and black gold myelin staining of MBP (**E**) and summary bar graphs (**F**) in the corpus callosum of rats. WMI was observed in the corpus callosum of the OGD 18°C group. *n* = 12 brain sections from 4 rats for both sham and OGD 18℃ groups, two-tailed unpaired *t* test for both black gold myelin staining and immunofluorescence staining of MBP, **P < 0.01, ***P < 0.001. Scale bar, 100 μm. MBP: Myelin Basic Protein. **G–I** Representative electron micrographs of myelinated axons (**G**). Summary bar graph of myelin thickness (**H**). Scatter plot with G-ratio on the y-axis and axon diameter on the x-axis (**I**). A reduction in myelin thickness was observed in the corpus callosum of the OGD 18°C group. *n* = 3 rats for both sham and OGD 18℃ groups, two-tailed unpaired *t* test for bar graph, simple linear regression of slopes for scatter plot, **P < 0.01. Scale bar, 400 nm. **J** Representative flow cytometry stained by Annexin V and PI in the corpus callosum of rats. The quadrants necrosis (Annexin V^-^/PI^+^), early apoptotic (Annexin V^+^/PI^-^), and late apoptotic/pyroptotic (Annexin V^+^/PI^+^) cell populations. *n* = 3 rats for both the sham and OGD 18℃ groups.

Hypothermic protection is widely utilized in clinical practice. However, hypothermia cannot completely prevent hypoxic-ischemic injury [39]. Notably, the myelinating white matter in the developing brain demonstrates particular susceptibility to hypoxic-ischemic injury [40]. To investigate the extent of injury under clinical hypothermia, we performed brain MRI on two pediatric patients with total anomalous pulmonary venous connection (TAPVC): one underwent surgery with deep hypothermic circulatory arrest (DHCA), while the other underwent surgery with cardiopulmonary bypass (CPB) alone. MRI revealed delayed brain development and white matter injury (WMI) in the DHCA pediatric patient, characterized by punctate lesions in the bilateral paraventricular white matter. On T1-weighted images, these lesions exhibited high signal intensity, whereas T2-weighted images showed low signal intensity (Fig. 1A), consistent with prior reports [7, 30, 41]. Fiber tractography further demonstrated reduced white matter volume and structural connectivity in the DHCA patient (Fig. 1B and Table S4). Microstructurally, fractional anisotropy (FA) values were significantly lower [42] in the corpus callosum, corona radiata, and centrum semiovale (Fig. 1C). These findings suggest that hypoxic-ischemic injury to the developing brain persists under hypothermic conditions.

To simulate hypothermic hypoxia-ischemia, we subjected cultured brain slices to hypothermic oxygen-glucose deprivation (OGD) at 18℃ (Fig. 1D), the optimal temperature for neuroprotection [18]. Despite its neuroprotective effects, the corpus callosum still showed significant damage following 60 min of hypothermic OGD, as evidenced by reduced myelin basic protein (MBP) immunofluorescence and Black Gold staining (Fig. 1E, F). Transmission electron microscopy (TEM) further confirmed thinner myelin sheaths and elevated G-ratios (Fig. 1G–I), indicating impaired myelination.

Next, we performed flow cytometry to analyze Annexin V and propidium iodide (PI) staining in the corpus callosum. There was a marked increase in Annexin V/PI double-positive cells in the OGD group (Fig. 1J). This pattern suggests pyroptotic cell death, distinguished by simultaneous phosphatidylserine exposure (Annexin V binding) and membrane rupture (PI uptake). In contrast, apoptosis typically features Annexin V single positivity, while necrosis is PI-dominant [43]. In summary, although hypothermia (18℃) confers neuroprotection, it cannot fully prevent OGD-induced WMI, which appears to be mediated, at least in part, through pyroptosis.

### Hypothermic OGD Triggers Microglia Pyroptosis in Corpus Callosum

**Fig. 2:**
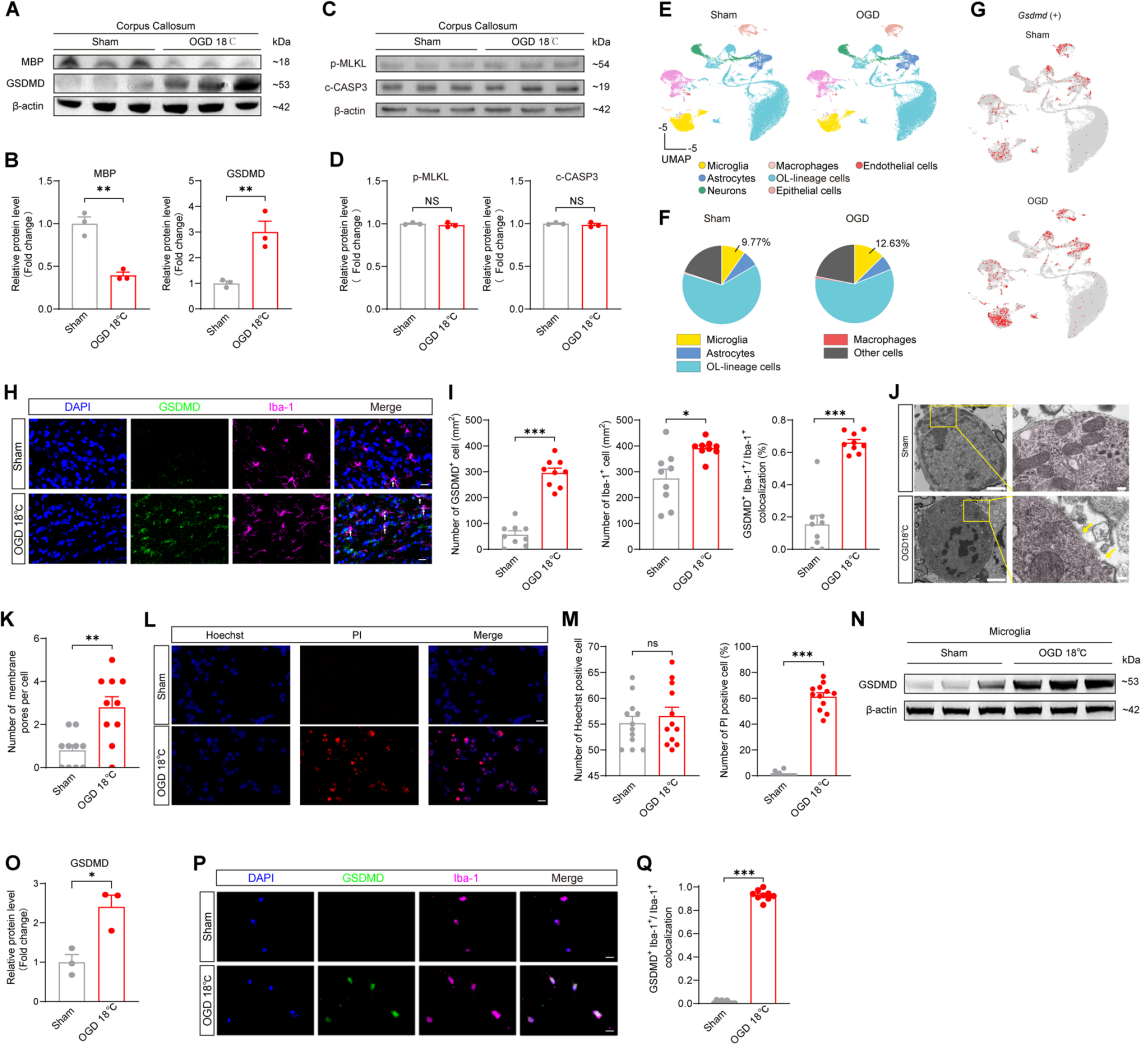
Hypothermic OGD triggers microglia pyroptosis in the corpus callosum. **A, B** Representative images of protein levels of MBP and GSDMD (**A**) and summary bar graphs (**B**) in the corpus callosum of rats. An increase in GSDMD protein and a decrease in MBP protein were observed in the OGD 18°C group. *n* = 3 rats for both the sham and OGD 18℃ groups, two-tailed unpaired *t* test for both MBP and GSDMD, **P < 0.01. OGD: oxygen and glucose deprivation. MBP: Myelin Basic Protein. **C, D** Representative images of protein levels of p-MLKL and c-CASP3 (**C**) and summary bar graphs (**D**) in the corpus callosum of rats. No significant difference in the protein levels of p-MLKL and c-CASP3 was observed between the sham and OGD 18°C groups. *n* = 3 rats for both sham and OGD 18℃ groups, two-tailed unpaired *t* test for both p-MLKL and c-CASP3, ns: not significant. **E** Single-cell RNA Sequencing analyses of corpus callosum. Based on their distinct transcriptomic profiles, cells were classified into seven clusters, which were annotated as microglia, astrocytes, neurons, macrophages, oligodendrocyte lineage cells (OL-lineage cells), epithelial cells, and endothelial cells. *n* = 3 rats for both the sham and OGD 18℃ groups. **F** The pie chart of the proportion of microglia in sham and OGD 18℃. An increased proportion of microglia was observed in the OGD 18°C group. *n* = 3 rats for both the sham and OGD 18℃ groups. **G** Feature plots of *Gsdmd* expression in sham and OGD 18℃. Upregulation of the *Gsdmd* gene expression was observed in the OGD 18°C group. *n* = 3 rats for both the sham and OGD 18℃ groups. **H, I** Representative images of immunofluorescence staining of GSDMD and Iba-1 (**H**) and summary bar graphs (**I**) in the corpus callosum of rats. The OGD 18°C group showed an increase in GSDMD-positive cells, concomitant with microglial accumulation and a consequent rise in the proportion of GSDMD-expressing microglia. *n* = 9 brain sections from 3 rats for both sham and OGD 18℃ groups, two-tailed unpaired *t* test for GSDMD positive cell‌, two-tailed unpaired *t* test with Welch’s correction for Iba-1 positive cell, and colocalization analysis, *P < 0.05, ***P < 0.001. Scale bar, 20 μm. The white arrow indicates colocalization of GSDMD and Iba-1. **J, K** Representative electron micrographs of membrane pores in microglia (**H**) and summary bar graph (**I**) in the corpus callosum of rats. An increased number of membrane pores was observed on microglia in the OGD 18°C group. *n* = 10 samples from 3 rats for both sham and OGD 18℃ groups, two-tailed unpaired *t* test, **P < 0.01. Scale bars, left: 2 μm; right: 200 nm. The red area indicates microglia. A membrane pore is located between the two yellow arrows. **L, M** Representative images of Hoechst and PI (Propidium Iodide) staining (L) and summary bar graphs (**M**) in microglia. More PI-positive cells were observed in primary cultured microglia from the OGD 18°C group. *n* = 12 cell samples from 4 rats for both the sham and OGD 18℃ groups, two-tailed unpaired *t* test for Hoechst positive cell, two-tailed unpaired *t* test with Welch’s correction for PI positive cell, ***P < 0.001, ns: not significant. Scale bar, 20 μm. **N, O** Representative images of protein levels of GSDMD (**N**) and summary bar graphs (**O**) in microglia. An increase in GSDMD protein was observed in primary cultured microglia from the OGD 18°C group. *n* = 3 samples from 3 rats for both the sham and OGD 18℃ groups, two-tailed unpaired *t* test, *P < 0.05. **P, Q** Representative images of immunofluorescence staining of GSDMD and Iba-1 (**P**) and summary bar graphs (**Q**) in microglia. More GSDMD-positive cells were observed in primary cultured microglia from the OGD 18°C group. *n* = 9 samples from 3 rats for both sham and OGD 18℃ groups, two-tailed Mann-Whitney tests, ***P < 0.001. Scale bar, 20 μm.

Gasdermin D (GSDMD) is a 487-amino acid cytoplasmic protein that is activated by cleaved caspase-1 (c-CASP1) to form membrane pores and functions as a key executor of pyroptosis [44, 45]. Based on this, we conducted RT-qPCR and western blotting to examine the changes in pyroptosis-related genes at mRNA and protein levels in the rat corpus callosum. Hypothermic OGD significantly upregulated the expression of NLRP3, GSDMD, and c-CASP1 in rat corpus callosum, concomitant with decreased MBP levels (Fig. 2A, B, and Fig. S1A–C). However, no significant increase in apoptosis- or necrosis-related proteins was detected (Fig. 2C, D). Previous studies have reported temperature-dependent characteristics of hypoxia-ischemia-induced WMI [46]. Our results also revealed that microglia pyroptosis increases with temperature (Figs. S2 and S3). Hypothermia-mediated neuroprotection may be partially attributed to the suppression of this process, although it cannot eliminate OGD-induced pyroptosis. Overall, these results demonstrated that hypothermic OGD induces pyroptosis in the corpus callosum.

To identify the specific cell types undergoing pyroptosis, we performed single-cell RNA sequencing (scRNA-seq) on the rat corpus callosum. Analysis using Seurat’s canonical correlation analysis (CCA) with UMAP visualization revealed distinct cellular clusters defined by lineage markers (Fig. 2E). The results showed an increased proportion of microglia (Fig. 2F). Furthermore, immunofluorescence staining revealed a significant increase in CD68-positive microglia, indicating microglial activation (Fig. S4). Importantly, feature plots first indicated that hypothermic OGD markedly up-regulated *Gsdmd* expression and that this increase was restricted almost exclusively to microglia (Fig. 2G and Fig. S5). Immunofluorescence staining subsequently validated the elevation of GSDMD protein and its predominant localization within microglia in hypothermic OGD (Fig. 2H, I and Fig. S6). Since the key feature of pyroptosis is the disruption of plasma membrane integrity [47], TEM analysis demonstrated pyroptosis-induced membrane damage in corpus callosum microglia, with significantly elevated pore formation (Fig. 2J, K).

To further confirm that hypothermic OGD can induce microglia pyroptosis, we subjected primary microglia to this condition. Results demonstrated that hypothermic OGD induced membrane rupture in microglia, as evidenced by PI uptake (Fig. 2L, M). Furthermore, western blotting and immunofluorescence revealed increased GSDMD expression in primary microglia following hypothermic OGD (Fig. 2N–Q). Collectively, these findings demonstrate that hypothermic OGD triggers microglia pyroptosis.

### WMI is Induced by IL-18 Release Through NF-κB/GSDMD-Dependent Pyroptosis

**Fig. 3:**
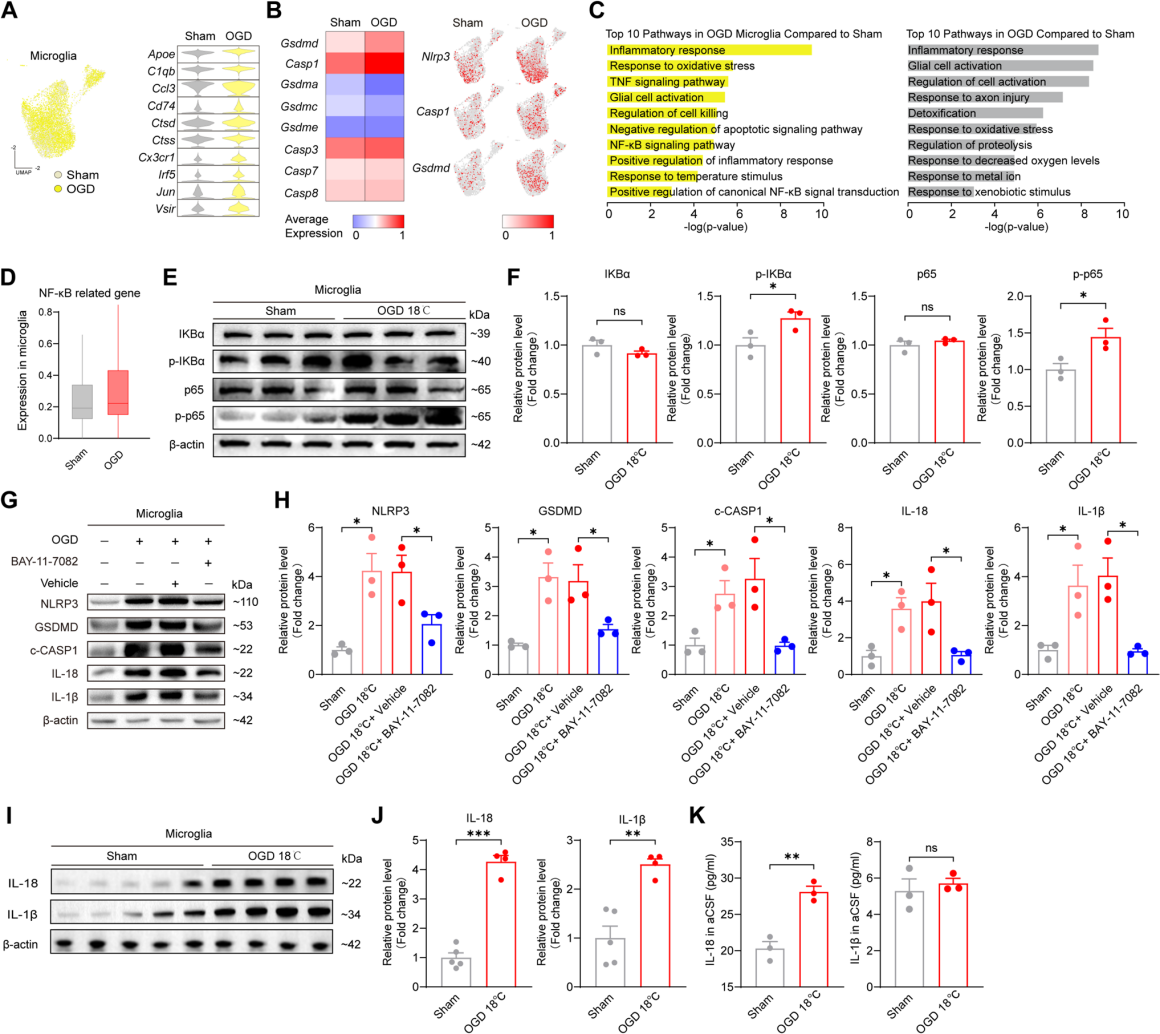
NF-κB/GSDMD drives microglia pyroptosis and subsequent IL-18 release. **A** Uniform manifold approximation and projection (UMAP) of microglia and a heat map of microglia activation-related gene expression in sham and OGD 18℃. Enhanced microglial activation was observed in the corpus callosum of the OGD 18°C group. *n* = 3 rats for both the sham and OGD 18℃ groups. OGD: oxygen and glucose deprivation. **B** Heat map of pyroptosis-related gene expression in sham and OGD 18℃ (left). Feature plots of *Gsdmd*, *Casp1*, and *Nlrp3* expression in sham and OGD 18℃ (right). Microglia in the OGD 18°C group showed an upregulation of GSDMD-dependent pyroptosis-related genes. *n* = 3 rats for both the sham and OGD 18℃ groups. **C** Enriched 10 pathway of OGD 18℃ microglia and corpus callosum tissue. *n* = 3 rats for both the sham and OGD 18℃ groups. **D** Box plot of NF-κb-related gene expression in the sham and OGD 18℃. Microglia in the OGD 18°C group showed an upregulation of NF-κB signaling pathway-related genes. *n* = 3 rats for both the sham and OGD 18℃ groups. **E, F** Representative images of protein levels of IKB, p-IKB, p65, and p-p65 (**E**) and summary bar graphs (**F**) in microglia. An increase in p-IKB and p-p65 protein was observed in the OGD 18°C group. No significant difference in the protein levels of IKB and p65 was observed between the sham and OGD 18°C groups. *n* = 3 cell samples for both sham and OGD 18℃ groups, two-tailed unpaired *t* test, *P < 0.05, ns: not significant. **G, H** Representative images of protein levels of NLRP3, GSDMD, c-CASP1, IL-18, and IL-1β (**G**) and summary bar graphs (**H**) in microglia. The protein levels of NLRP3, GSDMD, c-CASP1, IL-18, and IL-1β were elevated in microglia of the OGD 18°C group, which was suppressed by the NF-κB inhibitor BAY-11-7082. *n* = 3 cell samples for sham, OGD 18℃, OGD 18℃ + Vehicle and OGD 18℃ + BAY-11-7082 groups. Comparisons were made between the sham and OGD 18℃ groups, and between the OGD 18℃ + Vehicle and OGD 18℃ + BAY-11-7082 groups. Two-tailed unpaired *t* test with Welch’s correction for GSDMD between sham and OGD 18℃ groups and for IL-1β between OGD 18℃ + Vehicle and OGD 18℃ + BAY-11-7082 groups, two-tailed unpaired *t* test for the rest, *P < 0.05. **I, J** Representative images of protein levels of IL-18 and IL-1β (**I**) and summary bar graphs (**J**) in microglia. An increase in IL-18 and IL-1β protein was observed in the OGD 18°C group. *n* = 5 samples from 5 rats for the sham group and 4 samples from 4 rats for the OGD 18℃ group, two-tailed unpaired *t* test for IL-1β and IL-18, **P < 0.01, ***P < 0.001, ns: not significant. **K** Summary bar graphs of IL-1β and IL-18 levels in aCSF from the perfusion system measured by ELISA. *n* = 3 samples from 3 rats for both sham and OGD 18℃ groups, two-tailed unpaired *t* test for IL-18 and IL-1β, **P < 0.01, ns: not significant.

Having identified microglia pyroptosis as a key event, we next focused on elucidating the specific pyroptotic pathway operating in microglia. Consistent with previous findings, scRNA-seq revealed the activation of microglia following hypothermic OGD, as evidenced by the upregulation of specific chemokines (Fig. 3A). Heatmap and feature plots further confirmed hypothermic OGD triggers GSDMD-dependent pyroptosis, as indicated by the upregulation of *Nlrp3*, *Gsdmd,* and *Casp1* (Fig. 3B). To identify the signaling pathways mediating GSDMD-dependent pyroptosis in microglia, we utilized pathway enrichment analysis. We conducted analysis separately for the corpus callosum and microglia following hypothermic OGD. The corpus callosum showed inflammatory response and axonal injury, while microglia exhibited NF-κB-driven inflammation (Fig. 3C), which was further supported by the upregulation of NF-κB-related genes (Fig. 3D). Previous studies indicate that phosphorylation of p65 and IKB triggers activation of the NF-κB pathway [48]. To validate the activation of the NF-κB pathway in microglia, we examined the primary microglia following hypothermic OGD. Consistent with prior reports, hypothermic OGD significantly upregulated the expression of p-IKB and p-p65 (Fig. 3E, F). Notably, inhibition of NF-κB by BAY-11-7082 effectively suppressed pyroptosis in hypothermic OGD (Fig. 3G, H). Pathway enrichment analysis revealed NF-κB-driven inflammation in microglia. Because pyroptosis is known to trigger robust inflammatory responses through IL-18 and IL-1β release, and our cytokine array detected no upregulation of other inflammatory factors (Fig. S7A, B), we further examined whether hypothermic OGD specifically induces IL-18 and IL-1β release through microglia pyroptosis. Primary microglia showed upregulated protein levels of IL-18 and IL-1β following hypothermic OGD (Fig. 3I, J). However, ELISA analysis of IL-1β and IL-18 levels in aCSF revealed that only IL-18 was significantly elevated (Fig. 3K). These results demonstrate that the NF-κB/GSDMD pathway drives hypothermic OGD-induced microglia pyroptosis, and suggest that the inflammatory response in the corpus callosum may be mediated by IL-18.

**Fig. 4:**
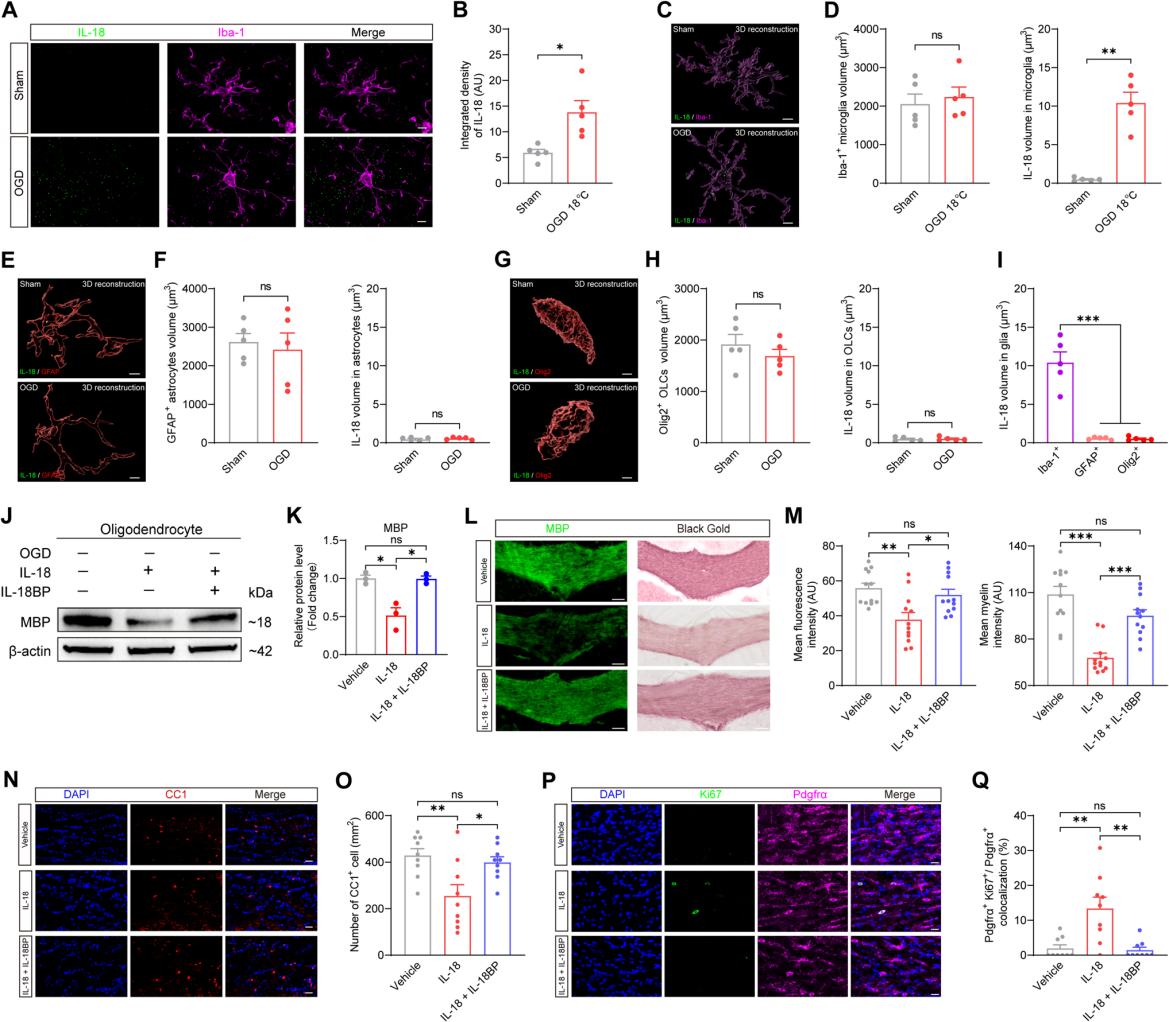
IL-18 derived from microglia pyroptosis leads to WMI. **A, B** Representative images of immunofluorescence staining of IL-18 and Iba-1 (**A**) and summary bar graphs (**B**) in the corpus callosum of rats. Increased IL-18 fluorescence intensity was observed in the OGD 18°C group. *n* = 5 rats for both the sham and OGD 18℃ groups, two-tailed unpaired *t* test with Welch’s correction for integrated density of IL-18, *P < 0.05. Scale bar, 5 μm. OGD: oxygen and glucose deprivation. **C, D** Imaris 3D-reconstruction of IL-18 and Iba-1 (**C**) and summary bar graphs (**D**) in the corpus callosum of rats. Enhanced intracellular IL-18 signal was observed within microglia of the OGD 18°C group. *n* = 5 rats for both sham and OGD 18℃ groups, two-tailed unpaired *t* test for microglia volume, two-tailed unpaired *t* test with Welch’s correction for IL-18 volume, **P < 0.01, ns: not significant. Scale bar, 5 μm. **E, F** Imaris 3D-reconstruction of IL-18 and GFAP (**E**) and summary bar graphs (**F**) in the corpus callosum of rats. A low level of IL-18 signal was detected in astrocytes, with comparable low levels in both the sham and OGD 18°C groups. *n* = 5 rats for both sham and OGD 18℃ groups, two-tailed unpaired *t* test, ns: not significant. Scale bar, 5 μm. **G, H** Imaris 3D-reconstruction of IL-18 and Olig2 (**G**) and summary bar graphs (**H**) in the corpus callosum of rats. A low level of IL-18 signal was detected in oligodendrocyte lineage cells, with comparable low levels in both the sham and OGD 18°C groups. *n* = 5 rats for both sham and OGD 18℃ groups, two-tailed unpaired *t* test, ns: not significant. Scale bar, 2 μm. OLCs: Oligodendrocyte Lineage Cells. **I** Summary bar graphs of intracellular IL-18 signal within microglia, astrocytes, and oligodendrocyte lineage cells. IL-18 signal was predominantly localized to microglia. *n* = 5 rats for both sham and OGD 18℃ groups, one-way ANOVA followed by Tukey’s post hoc test, ***P < 0.001. **J, K** Representative images of protein levels of MBP (**L**) and summary bar graphs (**M**) in oligodendrocyte. The protein level of MBP was decreased in oligodendrocyte of the IL-18 group, which was suppressed by the IL-18BP. *n* = 3 samples from 3 rats for Vehicle, IL-18 and IL-18 + IL-18BP groups, two-tailed unpaired *t* test between groups, *P < 0.05, ns: not significant. MBP: Myelin Basic Protein. **L, M** Representative images of black gold myelin staining and immunofluorescence staining of MBP (**L**) and summary bar graphs (**M**) in the corpus callosum of rats. WMI was observed in the corpus callosum of the IL-18 group, which was attenuated by IL-18BP. *n* = 12 brain sections from 4 rats for Vehicle, IL-18 and IL-18 + IL-18BP groups, two-tailed unpaired *t* test between groups, *P < 0.05, **P < 0.01, ***P < 0.001, ns: not significant. Scale bar, 100 μm. **N, O** Representative images of immunofluorescence staining of CC1 (**N**) and summary bar graphs (**O**) in the corpus callosum of rats. The IL-18 group showed a reduction in the number of mature oligodendrocytes, which was prevented by IL-18BP. *n* = 9 brain sections from 3 rats for Vehicle, IL-18 and IL-18 + IL-18BP groups, two-tailed unpaired *t* test between groups, *P < 0.05, **P < 0.01, ns: not significant. Scale bar, 20 μm. **P, Q** Representative images of immunofluorescence staining of Ki67 and Pdgfrα (**P**) and summary bar graphs (**Q**) in the corpus callosum of rats. An increase in Ki67⁺/PDGFRα⁺ double-positive cells was observed in the IL-18 group, which was suppressed by IL-18BP. *n* = 9 brain sections from 3 rats for Vehicle, IL-18 and IL-18 + IL-18BP groups, two-tailed unpaired *t* test with Welch’s correction between Vehicle and IL-18 groups, two-tailed unpaired *t* test with Welch’s correction between IL-18 and IL-18 + IL-18BP groups, two-tailed unpaired *t* test between Vehicle and IL-18 + IL-18BP groups, **P < 0.01, ns: not significant. Scale bar, 20 μm.

Given the specific elevation of IL-18, we investigated its cellular source. Immunofluorescence revealed enhanced IL-18 density in the corpus callosum following hypothermic OGD (Fig. 4A, B). 3D-reconstruction using Imaris demonstrated a primarily intracellular localization of IL-18 within microglia (Fig. 4C–I and Fig. S7C–F). Since pyroptosis primarily occurs in microglia following hypothermic OGD, these findings indicate that IL-18 is mainly derived from pyroptotic microglia.

Having confirmed WMI after hypothermic OGD, we next investigated whether IL-18 mediates the axonal demyelination. In primary oligodendrocyte cultures, IL-18 reduced MBP expression; this effect was abolished when the IL-18 binding protein IL-18BP was co-administered, confirming specificity (Fig. 4J, K). Ex vivo, IL-18 produced pronounced axonal demyelination in the corpus callosum, evident by both MBP immunofluorescence and Black-Gold staining (Fig. 4L, M), whereas IL-1β had no such impact (Fig. S8B–E). Accordingly, IL-18, rather than IL-1β, appears to be the principal cytokine driving hypothermic-OGD-induced white-matter injury (Fig. S8A). Finally, IL-18 administration decreased the number of mature (CC1^+^) oligodendrocytes (Fig. 4N, O) while simultaneously triggering reactive proliferation of oligodendrocyte precursor cells (OPCs) (Fig. 4P, Q). These findings demonstrate that IL-18 mediates axonal demyelination by inducing mature oligodendrocyte cell death, consistent with prior reports of its role in myelin damage [49]. The observed compensatory proliferation of OPCs likely represents an insufficient endogenous repair response to this degenerative process. In summary, we conclude that IL-18 derived from microglia pyroptosis is a key contributor to axonal demyelination and the subsequent development of WMI.

### DSF Alleviates Pyroptosis-Driven WMI in Weanling Rat Following Hypothermic OGD and DHCA

**Fig. 5:**
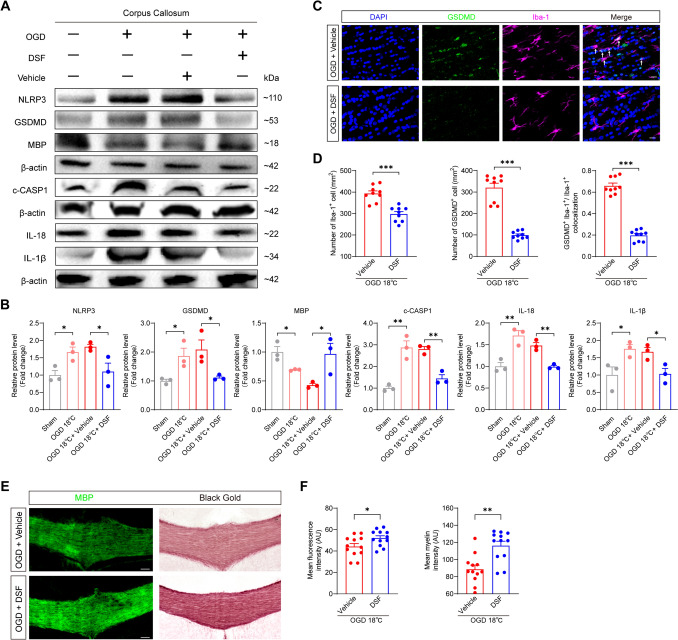
DSF inhibits microglia pyroptosis and alleviates pyroptosis-driven WMI in hypothermic OGD**. A, B** Representative images of protein levels of NLRP3, GSDMD, c-CASP1, MBP, IL-1β, and IL-18 (**A**) and summary bar graphs (**B**) in the corpus callosum of rats. The protein levels of NLRP3, GSDMD, c-CASP1, IL-18, and IL-1β were elevated in the corpus callosum of the OGD 18°C group, concomitant with a decrease in MBP. All these alterations were suppressed by DSF (Disulfiram). *n* = 3 rats for sham, OGD 18℃, OGD 18℃ + Vehicle and OGD 18℃ + DSF groups. Comparisons were made between the sham and OGD 18℃ groups, and between the OGD 18℃ + Vehicle and OGD 18℃ + DSF groups. Two-tailed unpaired *t* test with Welch’s correction for GSDMD between the OGD 18℃ + Vehicle and OGD 18℃ + DSF groups and for MBP between the sham and OGD 18℃ groups, two-tailed unpaired *t* test for the rest, *P < 0.05, **P < 0.01. OGD: oxygen and glucose deprivation. MBP: Myelin Basic Protein. **C, D** Representative images of immunofluorescence staining of GSDMD and Iba-1 (**C**) and summary bar graphs (**D**) in the corpus callosum of rats. DSF treatment reduced the number of GSDMD-positive cells following OGD; this was concomitant with a decrease in the total microglial population and, specifically, a reduction in the proportion of GSDMD-positive microglia. *n* = 9 brain sections from 3 rats for OGD 18℃ + Vehicle and OGD 18℃ + DSF groups, two-tailed unpaired *t* test for Iba-1 positive cell and colocalization analysis‌, two-tailed Mann-Whitney tests for GSDMD positive cell, ***P < 0.001. Scale bar, 20 μm. The white arrow indicates colocalization of GSDMD and Iba-1. **E, F** Representative images of black gold myelin staining and immunofluorescence staining of MBP (**E**) and summary bar graphs (**F**) in the corpus callosum of rats. DSF attenuated the OGD-induced WMI. *n* = 12 brain sections from 4 rats for both OGD 18℃ + Vehicle and OGD 18℃ + DSF groups, two-tailed unpaired *t* test for fluorescence intensity, two-tailed Mann-Whitney tests for myelin intensity, *P < 0.05, **P < 0.01. Scale bar, 100 μm.

Having established that the NF-κB/GSDMD/IL-18 axis in microglia contributes to axonal demyelination, we tested disulfiram (DSF), a GSDMD pore formation inhibitor, as a potential intervention. Western blotting demonstrated that DSF treatment reduced pyroptosis-related proteins (NLRP3, c-CASP1, GSDMD, IL-1β, and IL-18) in the corpus callosum, concomitant with increased MBP levels (Fig. 5A, B). Furthermore, immunofluorescence confirmed decreased GSDMD expression in microglia (Fig. 5C, D). This suppression of pyroptosis alleviated the severity of OGD-induced WMI in the corpus callosum (Fig. 5E, F).

**Fig. 6:**
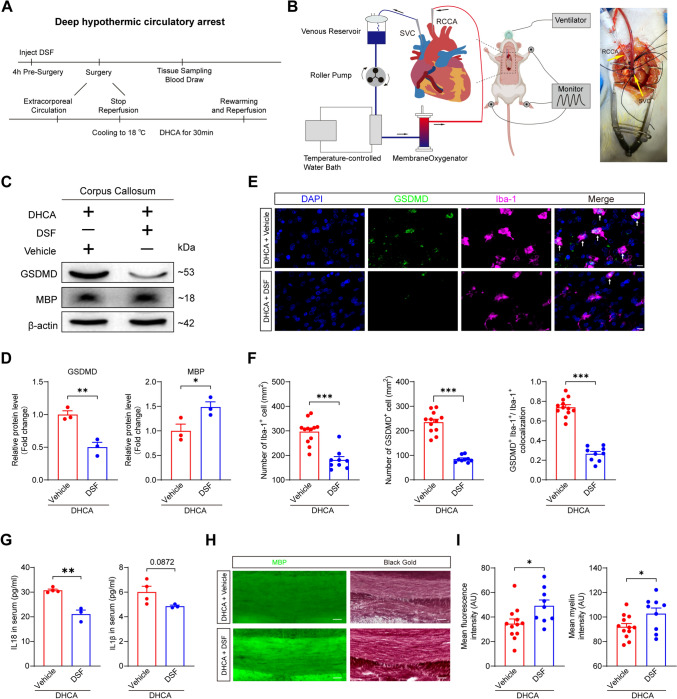
DSF inhibits microglia pyroptosis and alleviates pyroptosis-driven WMI in the weanling rat following DHCA. **A** Timeline of the weanling rat DHCA model. DSF (Disulfiram) was administered for 4 h before surgery. During the procedure, cardiopulmonary bypass was first established, followed by cooling to 18°C. Deep hypothermic circulatory arrest was then maintained for 30 min, after which rewarming was conducted, and cardiac function was restored. DHCA: deep hypothermic circulatory arrest. B Schematic illustration of DHCA in weanling rats (left). Vascular cannulation photographs in surgery (right). RCCA: right common carotid artery. SVC: superior vena cava. **C, D** Representative images of protein levels of GSDMD and MBP (**C**) and summary bar graphs (**D**) in the corpus callosum of weanling rats. Compared to the DHCA group, a decrease in GSDMD protein and an increase in MBP were observed in the corpus callosum following DSF administration. *n* = 3 rats for both DHCA + Vehicle and DHCA + DSF groups, two-tailed unpaired *t* test, *P < 0.05, **P < 0.01. MBP: Myelin Basic Protein. **E, F** Representative images of immunofluorescence staining of GSDMD and Iba-1 (**E**) and summary bar graphs (**F**) in the corpus callosum of weanling rats. Compared to the DHCA group, DSF administration reduced the number of GSDMD-positive cells in the corpus callosum; this was concomitant with a decrease in the total microglial population and, specifically, a reduction in the proportion of GSDMD-positive microglia. *n* = 12 brain sections from 4 rats for DHCA + Vehicle group, *n* = 9 brain sections from 3 rats for DHCA + DSF group, two-tailed unpaired *t* test with Welch’s correction for GSDMD positive cell, two-tailed Mann-Whitney tests for Iba-1 positive cell, two-tailed unpaired *t* test for colocalization analysis‌, ***P < 0.001. Scale bar, 20 μm. The white arrow indicates colocalization of GSDMD and Iba-1. **G** Summary bar graphs of IL-1β and IL-18 levels in serum following DHCA measured by ELISA. Compared to the DHCA group, serum levels of IL-18 and IL-1β were decreased following DSF administration. *n* = 4 rats for DHCA + Vehicle group, *n* = 3 rats for DHCA + DSF group, two-tailed unpaired *t* test for IL-18, two-tailed unpaired *t* test with Welch’s correction for IL-1β, **P < 0.01. **H, I** Representative images of black gold myelin staining and immunofluorescence staining of MBP (**H**) and summary bar graphs (**I**) in the corpus callosum of weanling rats. DSF attenuated the DHCA-induced WMI. *n* = 12 brain sections from 4 rats for DHCA + Vehicle group, *n* = 9 brain sections from 3 rats for DHCA + DSF group, two-tailed unpaired *t* test, *P < 0.05. Scale bar, 100 μm.

Further, we evaluated its prophylactic efficacy against pyroptosis-driven WMI in vivo. To facilitate the clinical translation of our findings, we established a DHCA model in weanling rats to investigate WMI in the developing brain. Given the fragile developing vasculature and hearts, we used a procedure that fully simulated open-chest surgery, involving 30 min DHCA followed by 40 min rewarming (Fig. 6A, B). Blood-gas analysis showed no inter-group differences in pH, Hb, Hct, Na⁺, or K⁺, confirming that DHCA did not disturb rat physiological status between DHCA and CPB groups (Fig. S9). Rats then received DSF (50 mg/kg, i.p.) 4h before surgery [26]. Western blotting analysis demonstrated that DSF administration reduced GSDMD protein levels while increasing MBP levels in rats following DHCA (Fig. 6C, D). This reduction in pyroptosis also occurred predominantly in microglia, as evidenced by decreased GSDMD expression in microglia by immunohistochemistry (Fig. 6E, F). Consistently, serum IL-18 and IL-1β were decreased post-DHCA (Fig. 6G). Corroborating our earlier findings, DSF treatment attenuated axonal demyelination, thereby alleviating pyroptosis-driven WMI in weanling rat DHCA model, as evidenced by reduced MBP immunofluorescence and Black Gold staining (Fig. 6H, I). Taken together, these findings establish that DSF-mediated inhibition of microglia pyroptosis represents a potential therapeutic strategy for protecting the developing brain from WMI following hypothermic hypoxia-ischemia.

## Discussion

In this study, we observed WMI in the pediatric patient and weanling rats following hypothermic hypoxia-ischemia. Using a hypothermic OGD model, we demonstrated that microglia activated the NF-κB signaling pathway, triggering pyroptosis and subsequent release of the inflammatory cytokine IL-18, which ultimately drives mature oligodendrocyte death and demyelination, resulting in WMI. Furthermore, by administering disulfiram (DSF), a pyroptosis inhibitor, we validated its protective effects against WMI in the hypothermic OGD model and the weanling rat DHCA model.

A recent study showed that microglia are the first brain cells to react to systemic inflammation induced by infarction [50], and they may exert neuroprotective effects [51]. Similarly, we found that microglia are the earliest responders to hypothermic OGD and subsequently undergo pyroptosis. This suggests that in hypothermic hypoxia-ischemia-induced white matter injury, microglia may play a crucial role. Moreover, microglia have a dual function in the brain, exerting either pro-inflammatory or anti-inflammatory effects [52–55]. Our study revealed that hypothermic OGD-induced pyroptotic microglia exhibited upregulation of specific chemokines, indicating their activation. Enrichment analysis further supported the conclusion that these microglia predominantly drive pro-inflammatory responses. On the other hand, under hypothermia, pyroptosis in other glia of the corpus callosum is relatively rare. This preferential induction of pyroptosis in microglia may thereby protect neighboring neural cells.

Pyroptosis is a robust inflammatory response [56]. Pyroptosis typically occurs in phagocytic cells, such as neutrophils and macrophages. Its morphological characteristics, mechanisms of occurrence, and regulation are distinct from apoptosis, autophagy, and necrosis, representing a distinct form of programmed cell death [57]. NF-κB represents a critical upstream regulator of pyroptosis that becomes potently activated under hypoxic-ischemic conditions [58]. This activation is particularly pronounced in microglia [59], where NF-κB orchestrates pyroptotic cell death through multiple effector mechanisms: inflammasome assembly, gasdermin-mediated pore formation, and synergistic interactions with hypoxia-responsive elements including HIF-1α. Importantly, the HIF-1α axis itself demonstrates temperature-dependent regulation [60], creating a feedback loop between hypoxic signaling and thermal modulation. The reduction of microglia pyroptosis observed with NF-κB pathway inhibition substantiates its pivotal role in hypoxic-ischemic pathogenesis, likely through modulation of pyroptotic signaling cascades under hypoxic-ischemic conditions. Notably, we found that as the temperature increased, the hypoxia-ischemia-induced microglia pyroptosis became more pronounced. Previous studies have reported that cold-inducible RNA-binding protein (CIRP) in microglia is also elevated during DHCA [46], suggesting that temperature may be a critical factor in microglia responses under hypoxic-ischemic conditions. Our results confirm that hypothermia indeed attenuates microglia pyroptosis, but the potential mechanisms through which temperature influences pyroptosis require further investigation. Hypothermia is widely recognized as a crucial neuroprotective strategy in hypoxic-ischemia. In our previous study, we observed that elevated perfusion temperature during OGD correlated with increased microglia activation and a reduction in MBP expression [18]. Our present study further reveals that deep hypothermia (18℃) cannot completely prevent the WMI caused by hypoxia-ischemia, though 18℃ is the most widely used temperature in clinical practice. If excessively low temperatures (such as below 18℃) were used, this would prolong the duration of rewarming, potentially exacerbating cryodamage to cellular structure, which could also lead to injury. Therefore, novel therapeutic strategies beyond temperature modulation must be developed to alleviate WMI following deep hypothermia hypoxia-ischemia.

Pyroptosis is classically mediated by CASP1, which catalyzes the cleavage of GSDMD and the precursors of IL-1β and IL-18, leading to the disruption of membrane integrity and the release of inflammatory mediators such as IL-1β and IL-18. The non-classical pathway, on the other hand, relies on CASP4/5/11, which directly acts on GSDMD and indirectly activates CASP1 to induce pyroptosis [61]. Hypoxia-ischemia potently induces NF-κB pathway activation *via* IKBα degradation and subsequent nuclear translocation of the p65/p50 heterodimer, initiating proinflammatory cascades that contribute to cellular demise. In our single-cell sequencing analysis of microglia, we observed a significant upregulation of key molecules in the classical pyroptosis pathway. The classical pyroptosis pathway is activated, as indicated by pathway enrichment analysis pointing to NF-κB, and ultimately leads to CASP1-mediated cleavage of GSDMD. This cleavage results in the formation of membrane pores that release cellular inflammatory factors into the extracellular space. Furthermore, we observed IL-18 enrichment in microglia and its extracellular release. As IL-18 is recognized as a critical factor contributing to demyelination [49], our results demonstrate that it induces cell death in mature oligodendrocytes, which may lead to demyelination. Interestingly, while the total number of oligodendrocyte lineage cells remained unchanged after hypothermic OGD, the inflammatory response triggered by IL-18 promotes the proliferation of oligodendrocyte precursor cells (OPCs). This likely represents a compensatory mechanism wherein OPCs proliferate for subsequent differentiation into oligodendrocytes to facilitate myelin repair. However, the precise mechanisms by which IL-18 selectively kills mature oligodendrocytes while stimulating OPC proliferation, as well as its comprehensive effects on the oligodendrocyte lineage, require further investigation.

In this study, while MRI revealed signs of WMI in an infant with TAPVC, the sample size is too small to rule out the influence of individual variations, making it impossible to draw generalizable clinical conclusions. This limitation stems from the practical challenges of performing prolonged MRI scans in pediatric patients. To enhance the reliability and clinical relevance of the present findings, our future studies will aim to expand the sample size and conduct systematic clinical cohort investigations. This should include enrolling more pediatric patients with various types of congenital heart disease who undergo DHCA or CPB surgery, combined with long-term postoperative neurodevelopmental follow-up data. Such efforts will allow for a more comprehensive assessment of the impact of different cardiac anomalies and surgical approaches on white matter development.

In the present work, we have for the first time established a weanling rat DHCA model involving thoracotomy, which more closely simulates the clinical scenario of surgical pediatric patients, to investigate changes in the developing brain and neuroprotective strategies. During this process, we have also paid attention to the duration of circulatory arrest [62] and the rewarming time [63], which are in line with current research standards. Our model primarily simulates cerebral WMI that occurs in the developing brain following hypothermic hypoxia-ischemia. Although this weanling rat DHCA model used in this study presents certain technical challenges that require specialized training, this technique facilitates further research into cognitive and neurodevelopmental improvements in pediatric patients. Therefore, we conclude that by elucidating the mechanisms of cerebral hypoxic-ischemic injury under hypothermic conditions using a DHCA model, targeted interventions can be implemented to enhance cerebral protection. Based on our mechanistic findings, we identified disulfiram as a potential therapeutic intervention. Originally used as an anti-alcohol drug, disulfiram was later found to inhibit pyroptosis. Studies have demonstrated that disulfiram improves cognitive function in Parkinson’s disease by suppressing microglia pyroptosis [26]. We administered disulfiram both in vitro (OGD) and in vivo (DHCA) and observed attenuated white matter demyelination in both settings. Although we were unable to identify specific subpopulations of microglia or receptors on microglia as precise targets for therapeutic intervention, results suggest that targeted inhibition of pyroptosis may represent a promising therapeutic strategy for hypothermic hypoxic-ischemic injury.

## Supplementary Information

Below is the link to the electronic supplementary material.

Supplementary file1 (PDF 1343 kb)

Supplementary file2 (PDF 1508 kb)
