# Supplementary material for: Microglia Pyroptosis-Derived IL-18 Drives White Matter Injury in Developing Brain following Hypothermic Hypoxia-Ischemia
Source: Neurosci Bull. 2026 Mar 9;42(6):1199–217. doi: 10.1007/s12264-026-01602-9 (PMC13221561; doi:10.1007/s12264-026-01602-9)
Supplement: Supplementary file 1 — Supplementary file1 (PDF 1343 kb) [file 12264_2026_1602_MOESM1_ESM.pdf]

Supplementary Materials

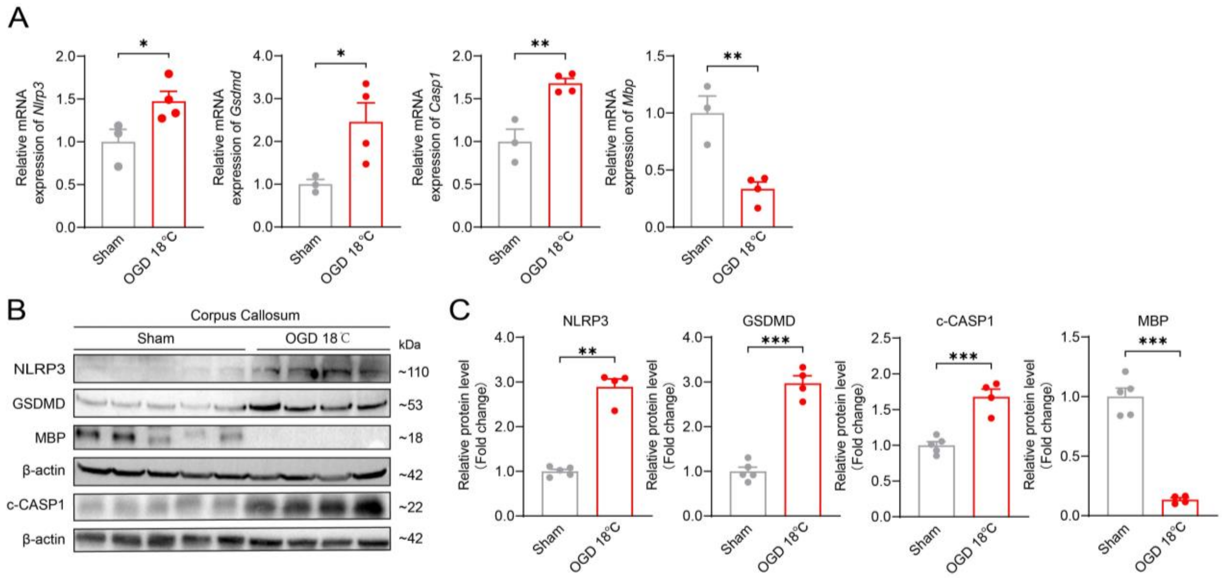

Fig. S1 The changes in protein levels of the pyroptosis-related gene in the rat corpus callosum. **A** Summary bar graphs of *Nlrp3*, *Gsdmd*, *Casp1,* and *Mbp* transcript levels in the corpus callosum of rats. $n = 3$ rats for sham group and 4 rats for OGD 18°C group, two-tailed unpaired t test for *Nlrp3*, *Gsdmd*, *Casp1*, and *Mbp*, $*P < 0.05$, $**P < 0.01$. OGD: oxygen and glucose deprivation. MBP: Myelin Basic Protein. **B, C** Representative images of protein levels of NLRP3, GSDMD, c-CASP1, and MBP (**B**) and summary bar graphs (**C**) in the corpus callosum of rats. $n = 5$ rats for sham group and 4 rats for OGD 18°C group, two-tailed unpaired t test for GSDMD and c-CASP1, two-tailed unpaired t test with Welch's correction for NLRP3 and MBP, $**P < 0.01$, $***P < 0.001$.

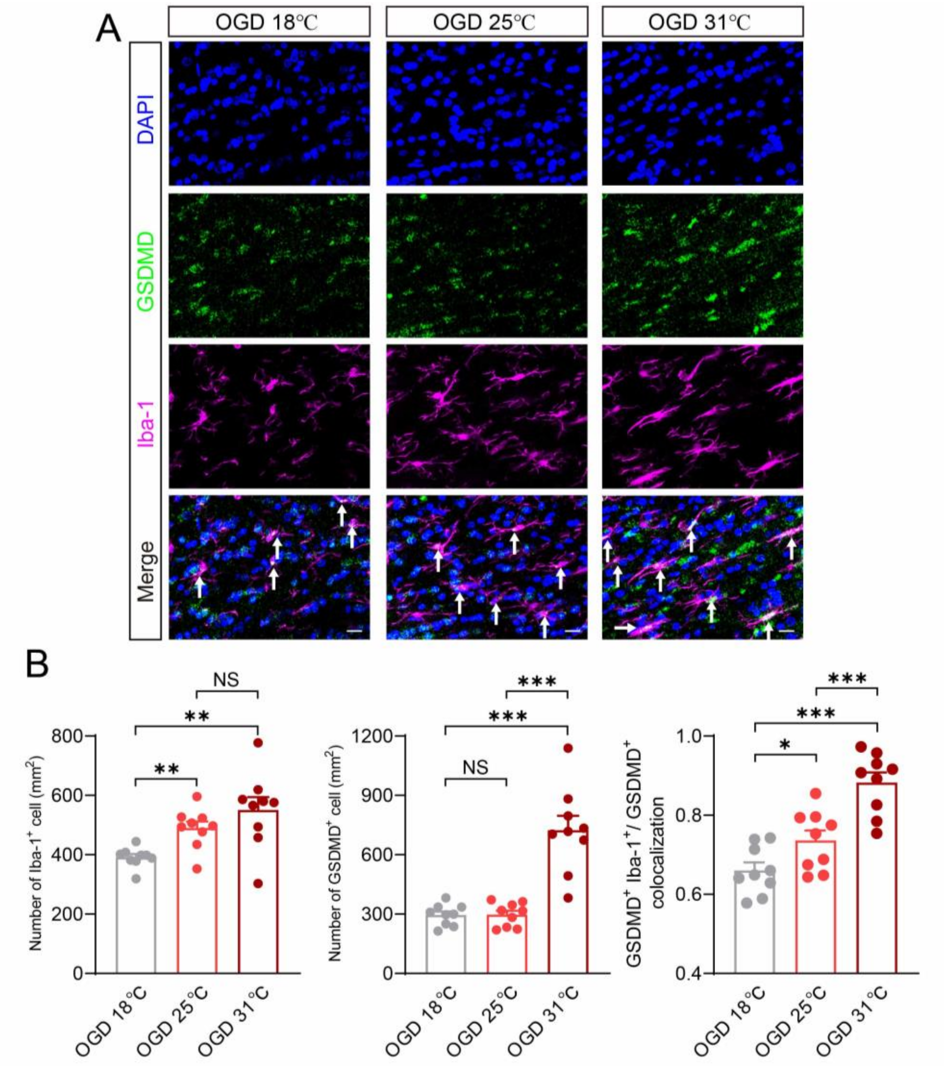

Fig. S2 Microglia pyroptosis following OGD at different temperatures. **A, B** Representative images of immunofluorescence staining of GSDMD and Iba-1 (**A**) and summary bar graphs (**B**) in the corpus callosum of rats. n = 9 brain sections from 3 rats for OGD 18°C, OGD 25°C and OGD 31°C groups, for Iba-1 positive cell, unpaired t test between OGD 18°C and OGD 25°C, OGD 25°C, and OGD 31°C groups, two-tailed unpaired t test with Welch's correction between OGD 18°C and OGD 31°C groups; for GSDMD positive cell, unpaired t test between OGD 18°C and OGD 25°C groups, two-tailed unpaired t test with Welch's correction between OGD 18°C and OGD 31°C groups, OGD 25°C and OGD 31°C; for colocalization analysis, unpaired t test between groups, *$P < 0.05$, **$P < 0.01$, ***$P < 0.001$, ns: not significant. Scale bar, 20 μm. OGD: oxygen and glucose deprivation.

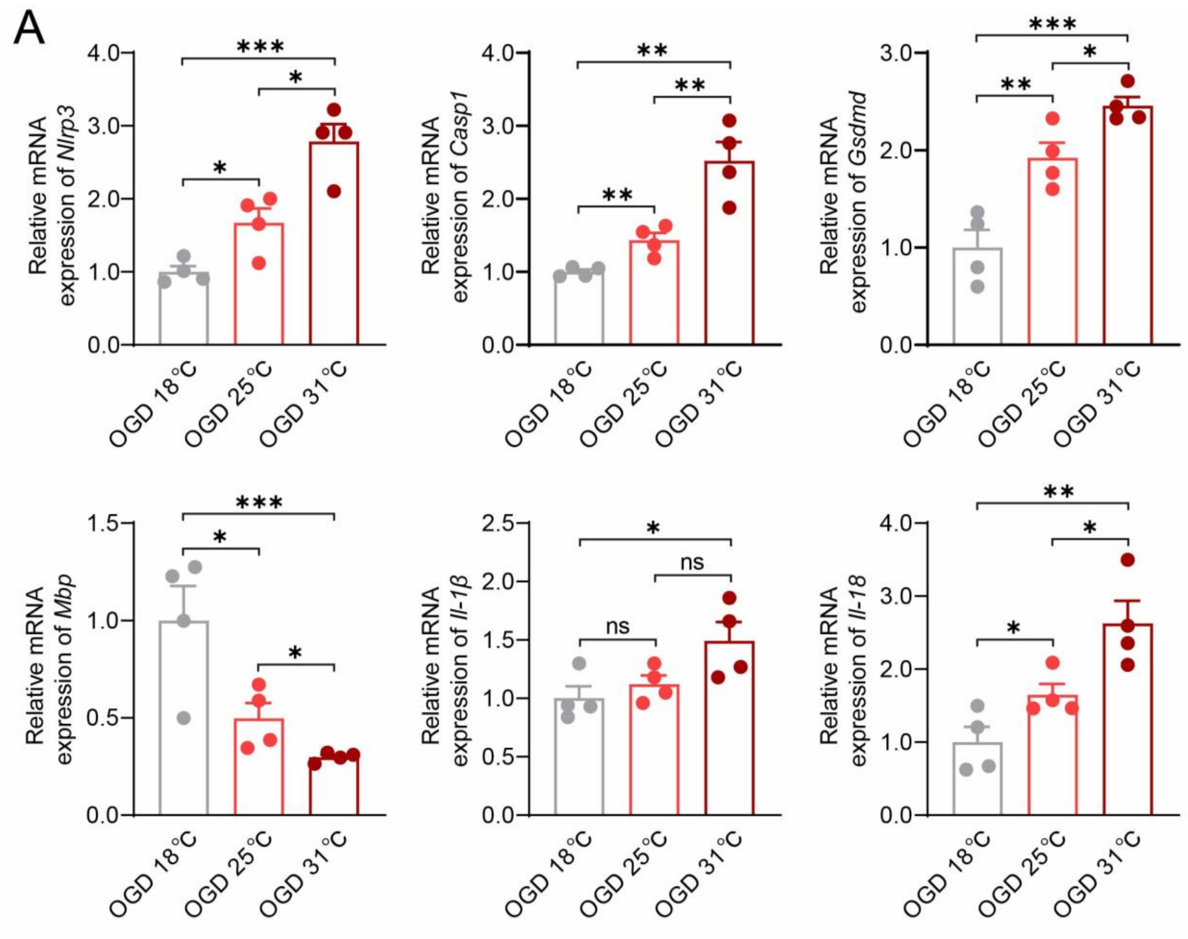

Fig. S3 The changes in mRNA levels of pyroptosis-related genes following OGD at different temperatures. **A** Summary bar graphs of *Nlrp3*, *Casp1*, *Gsdmd*, *Mbp*, *Il1β*, and *Il18* transcript levels in the corpus callosum of rats. n = 4 rats OGD 18°C, OGD 25°C and OGD 31°C groups, for *Nlrp3*, unpaired t test between groups; for *Casp1*, unpaired t test between OGD 18°C and OGD 25°C, OGD 25°C and OGD 31°C groups, two-tailed unpaired t test with Welch's correction between OGD 18°C and OGD 31°C groups; for *Gsdmd*, unpaired t test between groups; for *Mbp*, unpaired t test between OGD 18°C and OGD 25°C groups, two-tailed unpaired t test with Welch's correction between OGD 25°C and OGD 31°C groups, OGD 18°C and OGD 31°C groups; for *Il1β*, unpaired t test between groups; for *Il18*, unpaired t test between groups, *$P < 0.05$, **$P < 0.01$, ***$P < 0.001$, ns: not significant. OGD: oxygen and glucose deprivation. MBP: Myelin Basic Protein.

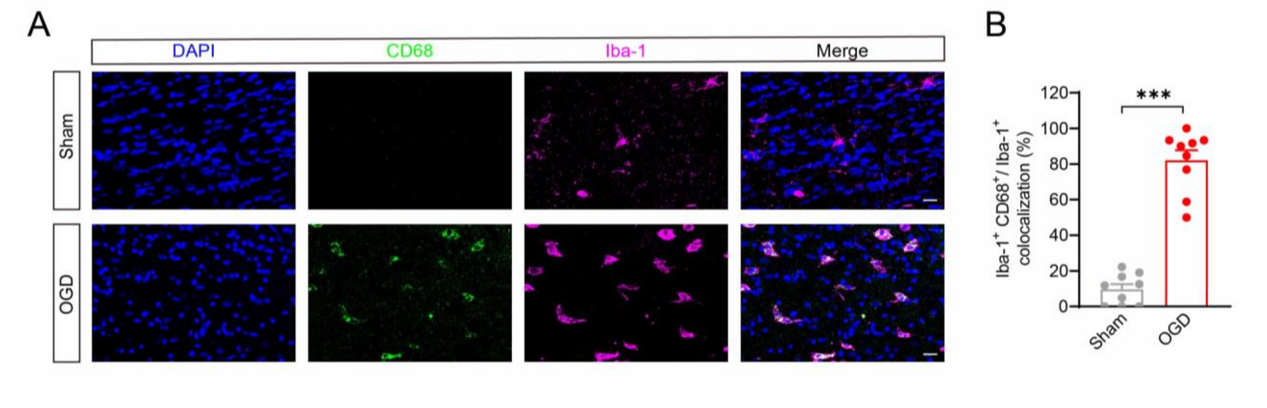

Fig. S4 The increase in CD68-positive microglia following OGD. **A, B** Representative images of immunofluorescence staining of CD68 and Iba-1 (**A**) and summary bar graphs (**B**) in the corpus callosum of rats. *n* = 9 brain sections from 3 rats for both sham and OGD 18°C groups, two-tailed unpaired *t* test, ****P* < 0.001. Scale bar, 20 μm. OGD: oxygen and glucose deprivation.

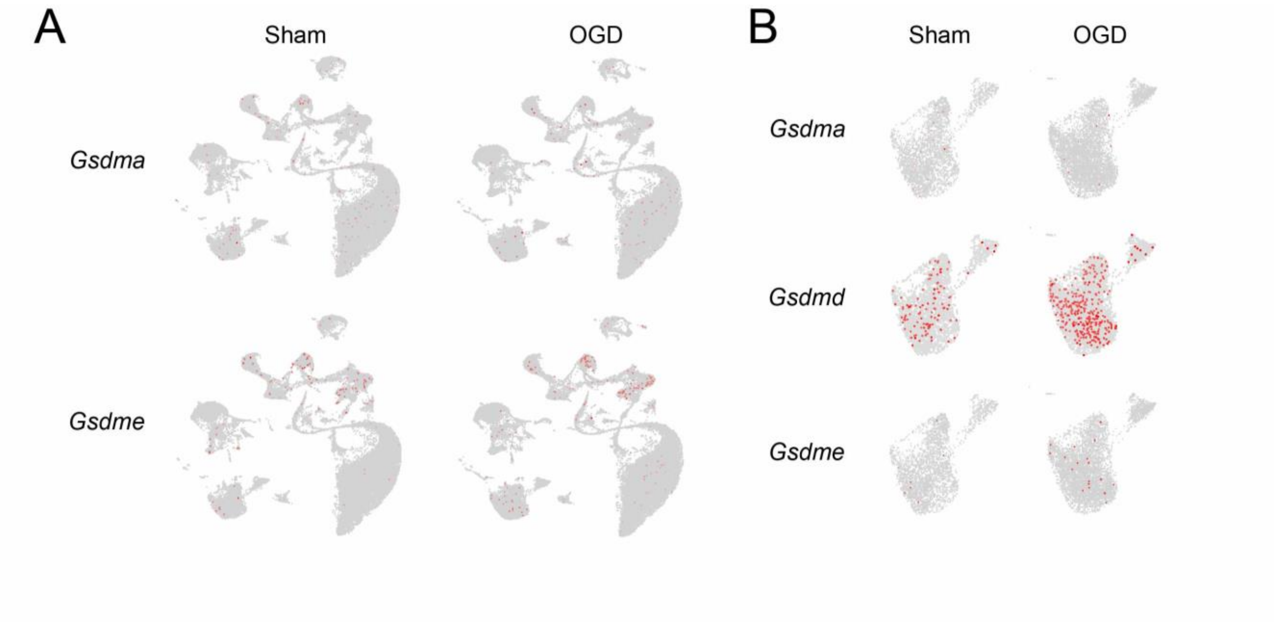

Fig. S5 The expression of *Gsdma* and *Gsdme* following OGD**. A** Feature plot of *Gsdma* and *Gsdme* expression in the sham and OGD 18°C groups. *n* = 3 rats for both the sham and OGD 18°C groups. **B** Feature plot of *Gsdma*, *Gsdme*, and Gsdmd expression in the sham and OGD 18°C groups. *n* = 3 rats for both the sham and OGD 18°C groups. OGD: oxygen and glucose deprivation.

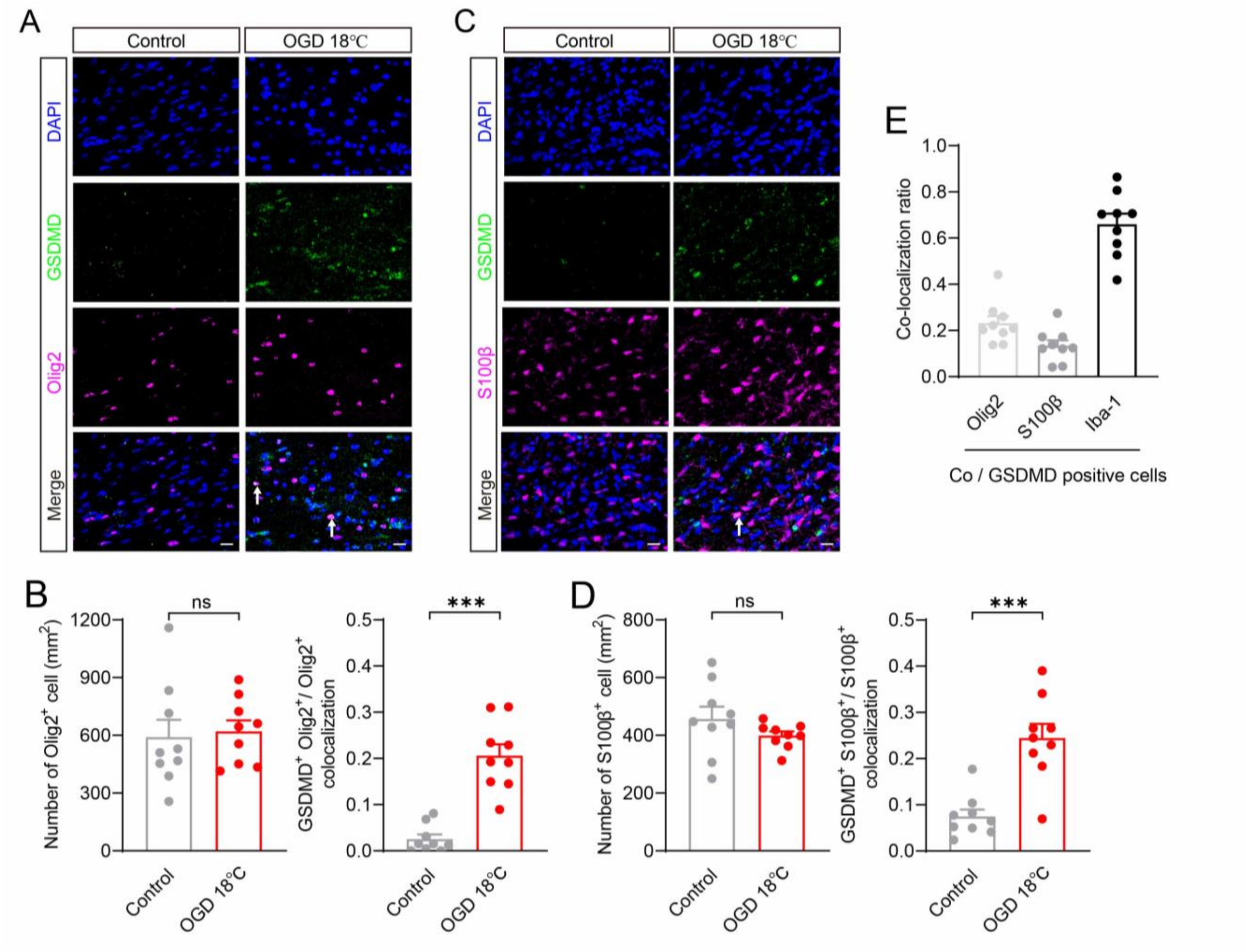

Fig. S6 The pyroptosis of oligodendrocyte lineage cells and astrocytes following OGD. **A, B** Representative images of immunofluorescence staining of GSDMD and Olig2 (**A**) and summary bar graphs (**B**) in the corpus callosum of rats. $n = 9$ brain sections from 3 rats for both sham and OGD 18℃ groups, two-tailed unpaired t test for Olig2 positive cell, two-tailed unpaired t test with Welch's correction for colocalization analysis, $^{***}P < 0.001$, ns: not significant. Scale bar, 20 μm. OGD: oxygen and glucose deprivation. **C, D** Representative images of immunofluorescence staining of GSDMD and S100β (**C**) and summary bar graphs (**D**) in the corpus callosum of rats. $n = 9$ brain sections from 3 rats for both sham and OGD 18℃ groups, two-tailed unpaired t test, $^{***}P < 0.001$, ns: not significant. Scale bar, 20 μm. **E** The ratio of dual-positive cells (colocalization of glia marker and GSDMD) to total GSDMD-positive cells. $n = 9$ brain sections from 3 rats for Olig2, S100β and Iba-1.

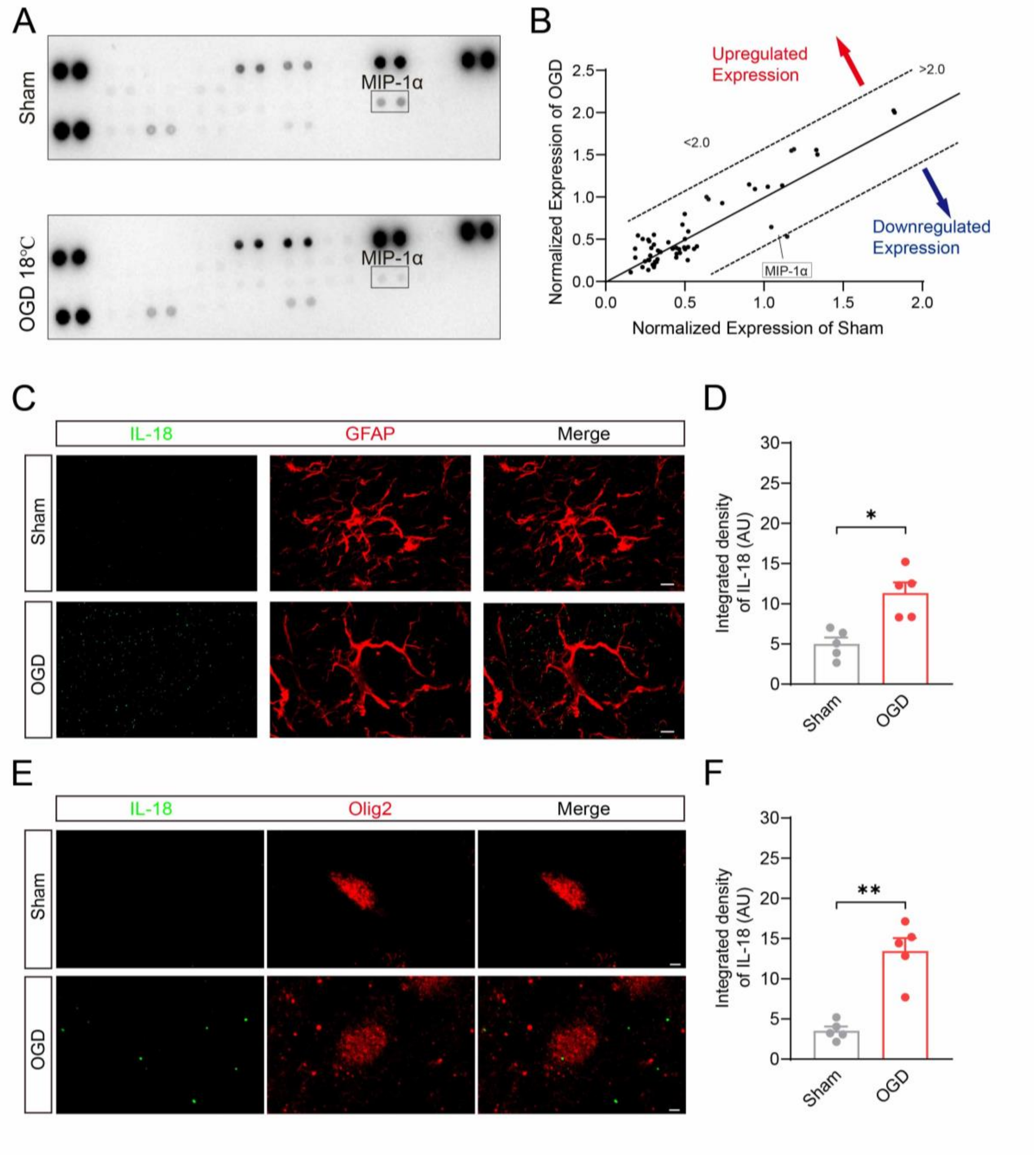

Fig. S7 Changes in various cytokines and IL-18 signal following OGD**. A, B** Representative images of Cytokine arrays (**A**) and scatter plot (**B**) in the corpus callosum of rats. $n = 4$ samples for both the sham and OGD 18℃ groups. OGD: oxygen and glucose deprivation. **C, D** Representative images of immunofluorescence staining of IL-18 and GFAP (**C**) and summary bar graphs (**D**) in the corpus callosum of rats. $n = 5$ rats for both the sham and OGD 18℃ groups, two-tailed unpaired t test, $^*P < 0.05$, ns: not significant. Scale bar, 5 μm. **E, F** Representative images of immunofluorescence staining of IL-18 and Olig2 (**E**) and summary bar graphs (**F**) in the corpus callosum of rats. $n = 5$ rats for both

the sham and OGD 18°C groups, two-tailed unpaired *t* test with Welch's correction, $^{**}P < 0.01$. Scale bar, 2 μm.

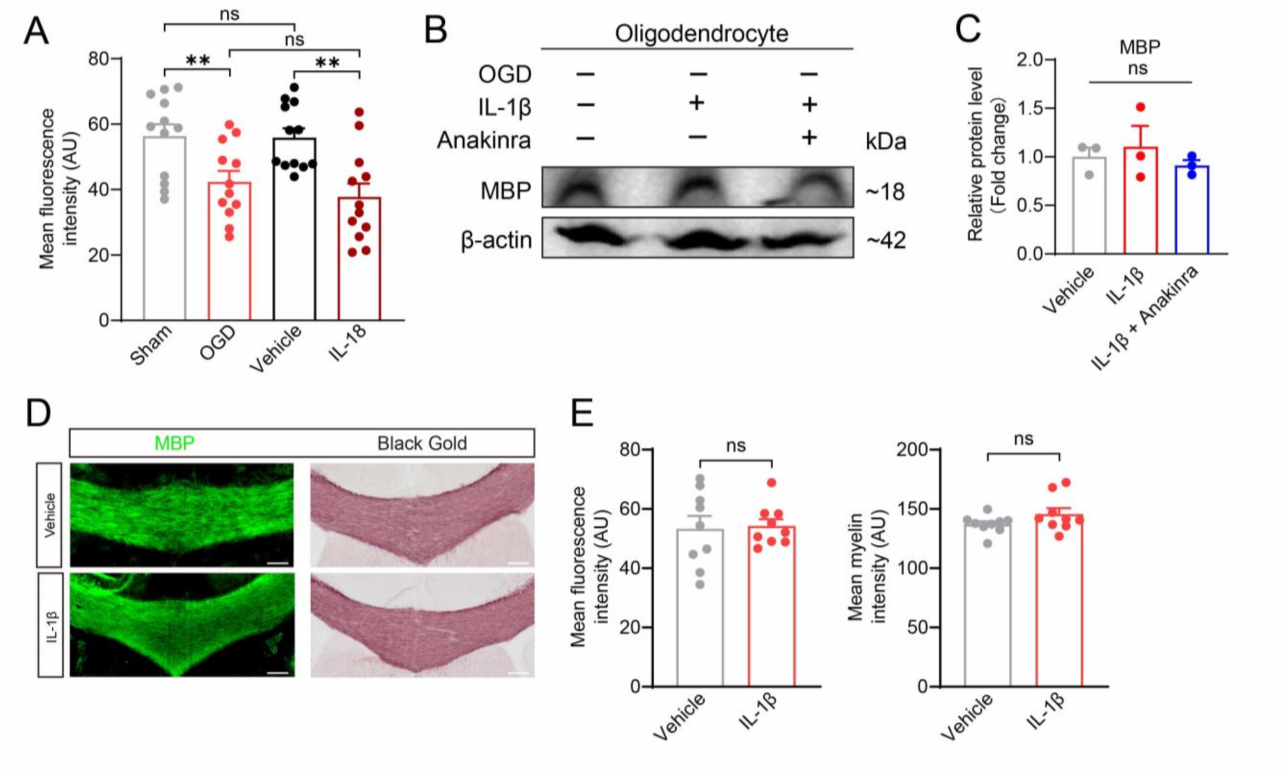

Fig. S8 IL-1β did not affect MBP expression in oligodendrocytes or myelin integrity in the corpus callosum. **A** Summary bar graphs across the various groups. *n* = 12 brain sections from 4 rats for Sham, OGD, Vehicle, and IL-18 groups, two-tailed unpaired *t* test between groups, $^{**}P < 0.01$, ns: not significant. OGD: oxygen and glucose deprivation. **B, C** Representative images of protein levels of MBP (B) and summary bar graphs (**C**) in oligodendrocytes. *n* = 3 samples from 3 rats for Vehicle, IL-1β, and IL-1β + Anakinra groups, two-tailed unpaired *t* test between groups, ns: not significant. MBP: Myelin Basic Protein. **D, E** Representative images of black gold myelin staining and immunofluorescence staining of MBP (**D**) and summary bar graphs (**E**) in the corpus callosum of rats. *n* = 12 brain sections from 4 rats for Vehicle and IL-1β, two-tailed unpaired *t* test between groups, ns: not significant. Scale bar, 100 μm.

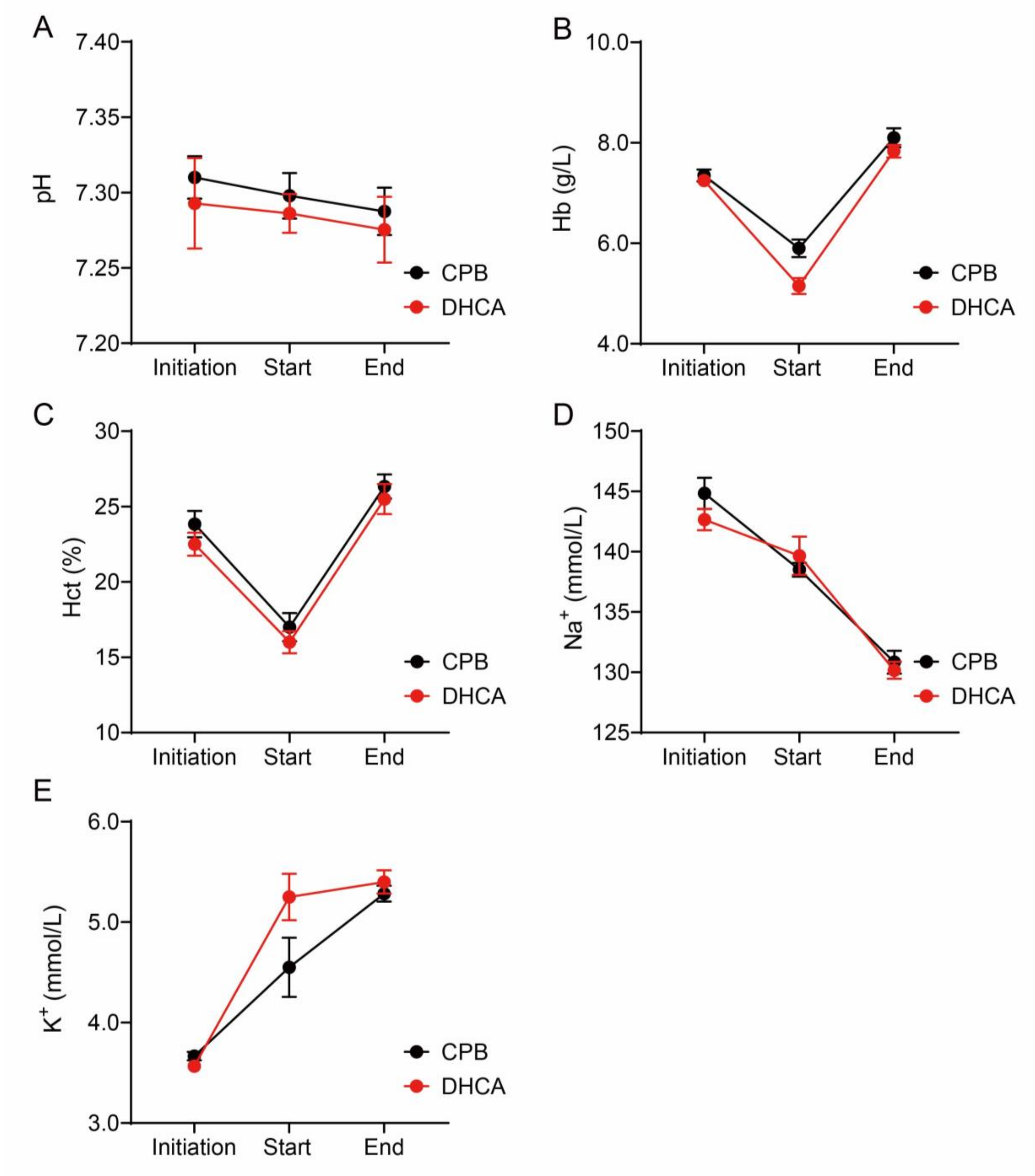

Fig. S9 The blood gas parameters of CPB and DHCA rats. **A–E.** Blood was collected and analyzed at three time points: initiation of surgery, start of cardiopulmonary bypass, and end of surgery. The analysis of pH (**A**), Hb (hemoglobin) (**B**), Hct (hematocrit) (**C**), Na^+ (**D**), and K^+ (**E**) data is shown in the figure. n = 4 rats for both CPB and DHCA groups; two-tailed unpaired t test for each time point; no significant. OGD: oxygen and glucose deprivation.

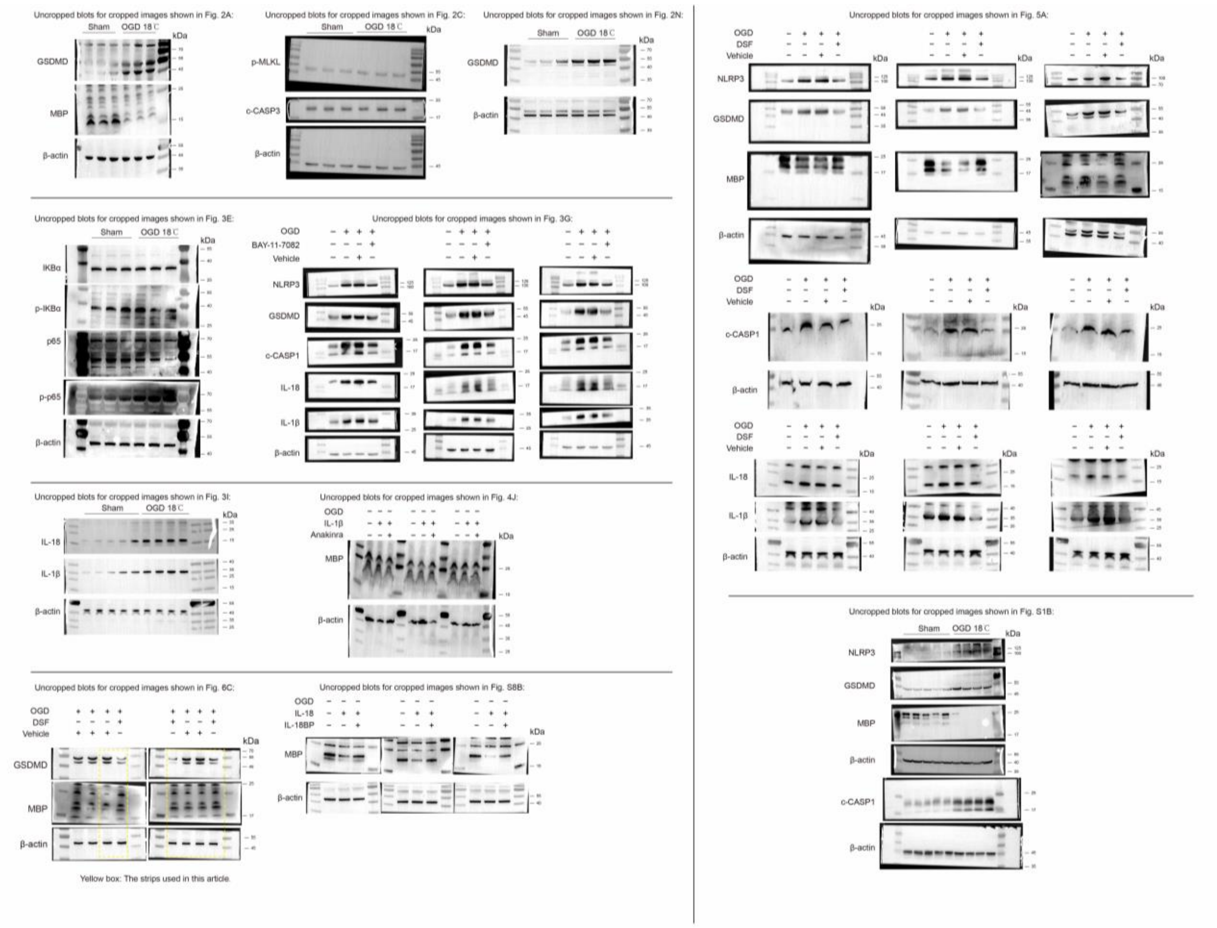

Fig. S10 The uncropped images of western blot data.

Table S1 Primer sequences used in the qPCR.

Gene	Primer sequences
Nlrp3	Forward:5'-CCAGACCTCCAAGACCACTACG-3'
	Reverse:5'-CAGAGAAGAGATGCTCCTCAATGC-3'
Caspase1	Forward:5'-GAACAACCAGAATTTAGGCTACAGATG-3'
	Reverse:5'-GTATTGGCTTCTTATTGGCACGATTC-3'
GSDMD	Forward:5'-CCTCCCTTCCCACAACATCTCC-3'
	Reverse:5'-GACTGAGTATGGTTCTTGGCTTCC-3'
Il-18	Forward:5'-TTCTCTGTGGTTCCATGCTTTCTG-3'
	Reverse:5'-AGGTTTGAGGCGGCTTTCTTTG-3'
Il-1B	Forward:5'-CACCTCACAAGCAGAGCACAAG-3'
	Reverse:5'-TTAGAAACAGTCCAGCCCATACTTTAG-3'
Mbp	Forward:5'-ACCGAGGAGAGGCTGGAAAGAAG-3'
	Reverse:5'-CTGCTGTGTGCTTGGAGTCTGTC-3'
Gapdh	Forward:5'-CAGCAATGCATCCTGCACC-3'
	Reverse:5'-TGGACTGTGGTCATGAGCCC-3'

Table S2 List of antibodies used in the article

Antibodies	SOURCE	IDENTIFIER
goat anti-Iba-1	Wako	Cat# 011-27991, RRID:AB_2935833
rabbit anti-GSDMD	Abcam	Cat# ab209845, RRID:AB_2783550
rabbit anti-MBP	Millipore	Cat# MAB382, RRID:AB_94971
rabbit anti-IL-18	Abcam	Cat# ab191152, RRID:AB_2737346
rabbit anti-S100β	Abcam	Cat# ab52642, RRID:AB_882426
mouse anti-Olig2	Millipore	Cat# MABN50, RRID:AB_10807410
rabbit anti-GFAP	Millipore	Cat# AB5804, RRID: AB_2109645
mouse anti-CD68	Abcam	Cat# ab955, RRID: AB_307338
mouse anti-Ki67	Cell Signaling	Cat# 9449, RRID: AB_2797703
mouse anti-APC (CC-1)	Millipore	Cat# OP80, RRID:AB_2057371
mouse anti-GSDMD	Santa Cruz	Cat# sc-393656, RRID:AB_2728694
rabbit anti-cleaved CASP1	Cell Signaling	Cat# 89332, RRID:AB_2923067
rabbit anti-NLRP3	Cell Signaling	Cat# 15101, RRID:AB_2722591
rabbit anti-β-actin	Invitrogen	Cat# MA515739, RRID:AB_10979409
rabbit anti-IKBα	Abcam	Cat# ab32518, RRID:AB_733068
rabbit anti-p-IKBα	Abcam	Cat# ab133462, RRID:AB_2801653
rabbit anti-p65	Abcam	Cat# ab16502, RRID:AB_2224674
rabbit anti-p-p65	Cell Signaling	Cat# 3033, RRID:AB_331284
rabbit anti-p-MLKL	Abcam	Cat# ab196436, RRID:AB_2687465
rabbit anti-cleaved CASP3	Cell Signaling	Cat# 9661, RRID:AB_2341188

Table S3 Clinical characteristics of 2 TAPVC patients.

Case number	Age	Preoperative SpO2	Birth weight (g)	Preterm	Type
1	1 momth	85%	3490	—	DHCA
2	2 month	85%	3320	—	CPB

Table S4 The numerical values of fiber bundle parameters

**Corpus Callosum**	**DHCA**	**CPB**
number of tracts	10776	19555
mean length(mm)	69.5308	78.6501
total volume(mm^3)	32324.8	40082.3

**Brainstem**	**DHCA**	**CPB**
number of tracts	18340	18428
mean length(mm)	87.9919	88.627525
total volume(mm^3)	12337.42	12854.5
