# Supplementary material for: Microglia Pyroptosis-Derived IL-18 Drives White Matter Injury in Developing Brain following Hypothermic Hypoxia-Ischemia
Source: Neurosci Bull. 2026 Mar 9;42(6):1199–217. doi: 10.1007/s12264-026-01602-9 (PMC13221561; doi:10.1007/s12264-026-01602-9)
Supplement: Supplementary file 2 — Supplementary file2 (PDF 1508 kb) [file 12264_2026_1602_MOESM2_ESM.pdf]

Differentially Expressed Genes

gene name	p_val	p_val_adj	cluster	avgExpr1	avgExpr2
Uba6	2.31E-06	0.04978	Microglia_SHAM_vs_Microglia_OGD	0.206316718	0.301983144
Cops6	2.28E-06	0.04921	Microglia_SHAM_vs_Microglia_OGD	0.440062597	0.558960892
Cc2d1a	2.26E-06	0.04863	Microglia_SHAM_vs_Microglia_OGD	0.038844948	0.083145795
Mad2l2	2.25E-06	0.0486	Microglia_SHAM_vs_Microglia_OGD	0.08890673	0.149688362
Pick1	2.24E-06	0.04829	Microglia_SHAM_vs_Microglia_OGD	0.038023715	0.087537716
Lgals8	2.24E-06	0.04822	Microglia_SHAM_vs_Microglia_OGD	0.441929059	0.583971688
Zbtb1	2.19E-06	0.04717	Microglia_SHAM_vs_Microglia_OGD	0.282066873	0.373340708
Gtf2f2	2.16E-06	0.04646	Microglia_SHAM_vs_Microglia_OGD	0.243509928	0.314881391
Psmb7	2.14E-06	0.04616	Microglia_SHAM_vs_Microglia_OGD	0.98478903	0.766530732
Pcmtd2	2.12E-06	0.04563	Microglia_SHAM_vs_Microglia_OGD	0.140081734	0.188104468
Mpst	2.11E-06	0.04549	Microglia_SHAM_vs_Microglia_OGD	0.113913779	0.182816898
Slc7a11	2.09E-06	0.04501	Microglia_SHAM_vs_Microglia_OGD	0.080499886	0.023502667
Api5	2.07E-06	0.04463	Microglia_SHAM_vs_Microglia_OGD	0.787036419	0.954644806
Sparcl1	2.06E-06	0.04451	Microglia_SHAM_vs_Microglia_OGD	0.108097879	0.205403432
Folr2	2.04E-06	0.04407	Microglia_SHAM_vs_Microglia_OGD	0.102172033	0.058937777
Mrps12	2.04E-06	0.04404	Microglia_SHAM_vs_Microglia_OGD	0.205295815	0.296087956
Camk1	2.02E-06	0.04344	Microglia_SHAM_vs_Microglia_OGD	0.283222803	0.390036349
Arrb2	1.98E-06	0.04277	Microglia_SHAM_vs_Microglia_OGD	0.636012048	0.759324605
Nfx1	1.98E-06	0.04265	Microglia_SHAM_vs_Microglia_OGD	0.408734053	0.502559926
Fkbp1a	1.93E-06	0.04168	Microglia_SHAM_vs_Microglia_OGD	1.412464151	1.679158744
Uts2b	1.93E-06	0.04151	Microglia_SHAM_vs_Microglia_OGD	0.028335392	0.004726252
Fbxl3	1.92E-06	0.04141	Microglia_SHAM_vs_Microglia_OGD	0.183857661	0.280481353
Odf2	1.91E-06	0.04123	Microglia_SHAM_vs_Microglia_OGD	0.22794076	0.3031448
Il16	1.91E-06	0.04115	Microglia_SHAM_vs_Microglia_OGD	0.055547035	0.105080016
Coq9	1.91E-06	0.04114	Microglia_SHAM_vs_Microglia_OGD	0.039571254	0.089682711
Atp5f1c	1.90E-06	0.04097	Microglia_SHAM_vs_Microglia_OGD	1.768832498	2.020954652
Fam151b	1.90E-06	0.04096	Microglia_SHAM_vs_Microglia_OGD	0.071612556	0.122664335
Piwil4	1.89E-06	0.04084	Microglia_SHAM_vs_Microglia_OGD	0.029224458	0.004058583
Rnf2	1.89E-06	0.04078	Microglia_SHAM_vs_Microglia_OGD	0.214448398	0.290460612
Esco1	1.89E-06	0.0407	Microglia_SHAM_vs_Microglia_OGD	0.305925591	0.394503968
Hivep2	1.89E-06	0.04068	Microglia_SHAM_vs_Microglia_OGD	0.122179742	0.054321369
Rrm1	1.88E-06	0.04062	Microglia_SHAM_vs_Microglia_OGD	0.082417114	0.143652384
Cltb	1.86E-06	0.0402	Microglia_SHAM_vs_Microglia_OGD	0.47019486	0.60179909
Slfn13	1.84E-06	0.03969	Microglia_SHAM_vs_Microglia_OGD	0.864323619	0.623914248
Eif3el1	1.83E-06	0.0395	Microglia_SHAM_vs_Microglia_OGD	0.390406313	0.489011634
Casp1	1.82E-06	0.03918	Microglia_SHAM_vs_Microglia_OGD	0.548070342	0.690725782
Wdr1	1.81E-06	0.03901	Microglia_SHAM_vs_Microglia_OGD	1.056931021	1.237884235
Pik3c2a	1.81E-06	0.03896	Microglia_SHAM_vs_Microglia_OGD	1.155510071	0.867611296
Rexo1	1.80E-06	0.03889	Microglia_SHAM_vs_Microglia_OGD	0.18447479	0.258480276
Ppp1r10	1.79E-06	0.03863	Microglia_SHAM_vs_Microglia_OGD	1.664584625	1.269895915
Sesn3	1.78E-06	0.03837	Microglia_SHAM_vs_Microglia_OGD	0.287452289	0.371501574
Clec4a1	1.77E-06	0.03826	Microglia_SHAM_vs_Microglia_OGD	0.459375573	0.598581752
Zc3h11a	1.77E-06	0.03825	Microglia_SHAM_vs_Microglia_OGD	0.833537553	0.978421004
Pm20d2	1.77E-06	0.03816	Microglia_SHAM_vs_Microglia_OGD	0.301261798	0.408302843
Glt8d1	1.76E-06	0.03785	Microglia_SHAM_vs_Microglia_OGD	0.150934454	0.223007032
Nlrp1a	1.75E-06	0.03783	Microglia_SHAM_vs_Microglia_OGD	0.215356429	0.302734049
Slc16a3	1.74E-06	0.0375	Microglia_SHAM_vs_Microglia_OGD	0.124442921	0.200346354
Tmem11	1.73E-06	0.03732	Microglia_SHAM_vs_Microglia_OGD	0.580180071	0.724419759
Ubxn1	1.73E-06	0.0373	Microglia_SHAM_vs_Microglia_OGD	1.298970689	1.505069212
Eif2b5	1.69E-06	0.03647	Microglia_SHAM_vs_Microglia_OGD	0.205845217	0.287779026
Anxa3	1.67E-06	0.03599	Microglia_SHAM_vs_Microglia_OGD	18.34026494	19.40501028
Sike1	1.66E-06	0.03578	Microglia_SHAM_vs_Microglia_OGD	0.084553906	0.144633762
Glmp	1.66E-06	0.0357	Microglia_SHAM_vs_Microglia_OGD	0.911427471	1.109804492
Actl6a	1.64E-06	0.0354	Microglia_SHAM_vs_Microglia_OGD	0.137490319	0.202716116
Zfand5	1.64E-06	0.0354	Microglia_SHAM_vs_Microglia_OGD	2.482142476	2.014158449
Mprip	1.61E-06	0.0347	Microglia_SHAM_vs_Microglia_OGD	0.464139839	0.302938664
Tlr3	1.58E-06	0.03408	Microglia_SHAM_vs_Microglia_OGD	0.267452679	0.378011395
Cdyl	1.58E-06	0.03404	Microglia_SHAM_vs_Microglia_OGD	0.114456975	0.1768584
Sla	1.58E-06	0.03398	Microglia_SHAM_vs_Microglia_OGD	0.442837935	0.281119846
Foxn3	1.56E-06	0.03355	Microglia_SHAM_vs_Microglia_OGD	2.623935232	2.941314174
Vps28	1.54E-06	0.03327	Microglia_SHAM_vs_Microglia_OGD	0.541302353	0.636557054
Ppp2r5a	1.49E-06	0.03202	Microglia_SHAM_vs_Microglia_OGD	0.546597951	0.637285328
Tmem33	1.48E-06	0.03193	Microglia_SHAM_vs_Microglia_OGD	0.713388504	0.858347591
Edem1	1.48E-06	0.03186	Microglia_SHAM_vs_Microglia_OGD	0.67524812	0.844259027
Rhov	1.48E-06	0.03185	Microglia_SHAM_vs_Microglia_OGD	0.015057963	0.057148316
Slc25a28	1.48E-06	0.03185	Microglia_SHAM_vs_Microglia_OGD	0.208359291	0.290622717
Abi1	1.47E-06	0.03169	Microglia_SHAM_vs_Microglia_OGD	1.612355476	1.318554952
Mbd2	1.47E-06	0.03159	Microglia_SHAM_vs_Microglia_OGD	0.248841338	0.150667055
Klhdc2	1.44E-06	0.03115	Microglia_SHAM_vs_Microglia_OGD	0.095969202	0.142700604
Sall1	1.44E-06	0.03109	Microglia_SHAM_vs_Microglia_OGD	0.610842625	0.772095645
Fam72a	1.44E-06	0.03097	Microglia_SHAM_vs_Microglia_OGD	0.079131425	0.146309564
Wasl	1.42E-06	0.03071	Microglia_SHAM_vs_Microglia_OGD	0.588577475	0.693044979
Rcl1	1.41E-06	0.03033	Microglia_SHAM_vs_Microglia_OGD	0.157168169	0.235523067
Nudt8	1.39E-06	0.02996	Microglia_SHAM_vs_Microglia_OGD	0.034543784	0.08867517
Tmem65	1.38E-06	0.02981	Microglia_SHAM_vs_Microglia_OGD	0.527261348	0.635450374
St6galnac4	1.38E-06	0.02974	Microglia_SHAM_vs_Microglia_OGD	0.098607167	0.151542643
Ythdf1	1.36E-06	0.02929	Microglia_SHAM_vs_Microglia_OGD	0.526602299	0.627284638
Mctp1	1.33E-06	0.02876	Microglia_SHAM_vs_Microglia_OGD	0.363522263	0.481456566
Dnm1l	1.33E-06	0.02871	Microglia_SHAM_vs_Microglia_OGD	0.579606165	0.687803344
Msantd3	1.30E-06	0.02791	Microglia_SHAM_vs_Microglia_OGD	0.034603461	0.005623151
Tcof1	1.29E-06	0.02776	Microglia_SHAM_vs_Microglia_OGD	0.087462362	0.155151971
Ssh2	1.28E-06	0.02754	Microglia_SHAM_vs_Microglia_OGD	1.406485157	1.521554104
Aif1	1.27E-06	0.02745	Microglia_SHAM_vs_Microglia_OGD	15.13332528	16.10807623
Neu1	1.27E-06	0.02743	Microglia_SHAM_vs_Microglia_OGD	0.47063787	0.565115439

Tbrg1	1.27E-06	0.02739	Microglia_SHAM_vs_Microglia_OGD	0.065195565	0.122282271
Caprin1	1.27E-06	0.0273	Microglia_SHAM_vs_Microglia_OGD	0.828560575	0.981617769
Capza1	1.26E-06	0.02711	Microglia_SHAM_vs_Microglia_OGD	1.117432125	1.354614621
Cuta	1.25E-06	0.02702	Microglia_SHAM_vs_Microglia_OGD	0.772391849	0.916790945
Atp5pf	1.25E-06	0.02698	Microglia_SHAM_vs_Microglia_OGD	1.519719027	1.870243429
Bnip2	1.24E-06	0.02681	Microglia_SHAM_vs_Microglia_OGD	0.880243437	1.040035394
Pin1	1.24E-06	0.02676	Microglia_SHAM_vs_Microglia_OGD	0.63688736	0.754930598
Tmem50a	1.20E-06	0.02585	Microglia_SHAM_vs_Microglia_OGD	1.660956655	1.968690916
Qars1	1.19E-06	0.0257	Microglia_SHAM_vs_Microglia_OGD	0.227192822	0.31883289
Psme2	1.19E-06	0.02566	Microglia_SHAM_vs_Microglia_OGD	1.033395335	1.237391773
Mrps18c	1.19E-06	0.02563	Microglia_SHAM_vs_Microglia_OGD	0.242435872	0.331050057
Primpol	1.18E-06	0.0255	Microglia_SHAM_vs_Microglia_OGD	0.073695515	0.134478293
Zfp830	1.17E-06	0.02513	Microglia_SHAM_vs_Microglia_OGD	0.184234002	0.270341644
Eif4a3	1.16E-06	0.02499	Microglia_SHAM_vs_Microglia_OGD	0.931196428	1.067644944
Mgat2	1.15E-06	0.02478	Microglia_SHAM_vs_Microglia_OGD	0.245440663	0.31895335
Csf2ra	1.13E-06	0.02433	Microglia_SHAM_vs_Microglia_OGD	0.183719173	0.280770222
RT1-Da	1.11E-06	0.02388	Microglia_SHAM_vs_Microglia_OGD	1.512046629	1.739018524
Tomm20	1.09E-06	0.02354	Microglia_SHAM_vs_Microglia_OGD	3.60382012	4.026591339
Pip5k1a	1.08E-06	0.02331	Microglia_SHAM_vs_Microglia_OGD	0.768648536	0.522128265
Magi3	1.08E-06	0.02328	Microglia_SHAM_vs_Microglia_OGD	0.239394591	0.148980393
Map3k4	1.08E-06	0.02319	Microglia_SHAM_vs_Microglia_OGD	0.288459879	0.384231307
Cd300a	1.06E-06	0.02279	Microglia_SHAM_vs_Microglia_OGD	0.102103349	0.159769883
Ndufb2	1.05E-06	0.02265	Microglia_SHAM_vs_Microglia_OGD	0.984879661	1.213966386
Asb8	1.04E-06	0.02248	Microglia_SHAM_vs_Microglia_OGD	0.228660149	0.335024687
Mpc2	1.03E-06	0.02226	Microglia_SHAM_vs_Microglia_OGD	0.212552426	0.272682465
Zfp358	1.03E-06	0.02219	Microglia_SHAM_vs_Microglia_OGD	0.073973409	0.128821596
Col4a3	1.02E-06	0.02197	Microglia_SHAM_vs_Microglia_OGD	0.094289183	0.030421142
Mrc2	1.02E-06	0.02197	Microglia_SHAM_vs_Microglia_OGD	0.168865932	0.245069733
Wdr44	1.01E-06	0.02181	Microglia_SHAM_vs_Microglia_OGD	0.390871512	0.50963934
Trim28	1.01E-06	0.02179	Microglia_SHAM_vs_Microglia_OGD	0.423083596	0.539938965
Phtf1	1.01E-06	0.0217	Microglia_SHAM_vs_Microglia_OGD	0.181632583	0.26078691
Cfap20	9.95E-07	0.02146	Microglia_SHAM_vs_Microglia_OGD	0.226351808	0.328968923
Cdip1	9.78E-07	0.02109	Microglia_SHAM_vs_Microglia_OGD	0.164298734	0.243999959
Rnh1	9.78E-07	0.02109	Microglia_SHAM_vs_Microglia_OGD	1.89650229	1.478629945
Psip1	9.76E-07	0.02105	Microglia_SHAM_vs_Microglia_OGD	0.43252295	0.561428471
Pomk	9.75E-07	0.02103	Microglia_SHAM_vs_Microglia_OGD	0.046029017	0.097490513
Nedd4l	9.74E-07	0.021	Microglia_SHAM_vs_Microglia_OGD	1.626132947	1.213904815
Thyn1	9.65E-07	0.0208	Microglia_SHAM_vs_Microglia_OGD	0.147124498	0.204049161
Snx18	9.55E-07	0.02059	Microglia_SHAM_vs_Microglia_OGD	3.92334992	3.391467499
U6.430	9.53E-07	0.02056	Microglia_SHAM_vs_Microglia_OGD	0.034618258	0.008549575
Pkn2	9.52E-07	0.02052	Microglia_SHAM_vs_Microglia_OGD	0.52798556	0.63516186
Sigmar1	9.51E-07	0.0205	Microglia_SHAM_vs_Microglia_OGD	0.187501318	0.247535506
Ccdc84	9.50E-07	0.02048	Microglia_SHAM_vs_Microglia_OGD	0.159487864	0.240217984
Nnt	9.42E-07	0.02032	Microglia_SHAM_vs_Microglia_OGD	0.257275971	0.33136578
Smim12	9.28E-07	0.02001	Microglia_SHAM_vs_Microglia_OGD	0.130762591	0.190493824
Icoslg	9.17E-07	0.01976	Microglia_SHAM_vs_Microglia_OGD	3.666047854	4.121037708
Ctbp2	9.16E-07	0.01975	Microglia_SHAM_vs_Microglia_OGD	0.172198697	0.273480914
Kdelr1	9.04E-07	0.01949	Microglia_SHAM_vs_Microglia_OGD	0.655259674	0.786131359
Plbd2	9.01E-07	0.01942	Microglia_SHAM_vs_Microglia_OGD	0.743631198	0.90546727
Rp2	8.89E-07	0.01918	Microglia_SHAM_vs_Microglia_OGD	0.093944615	0.15922243
Washc4	8.88E-07	0.01914	Microglia_SHAM_vs_Microglia_OGD	0.393786768	0.492917839
Oxa1l	8.78E-07	0.01894	Microglia_SHAM_vs_Microglia_OGD	0.331038537	0.418692533
RT1-Ba	8.77E-07	0.01891	Microglia_SHAM_vs_Microglia_OGD	0.838781994	1.002912804
Prxl2c	8.70E-07	0.01875	Microglia_SHAM_vs_Microglia_OGD	0.131559828	0.216002664
Ankrd6	8.69E-07	0.01873	Microglia_SHAM_vs_Microglia_OGD	0.57136916	0.706081501
Slc25a4	8.63E-07	0.01862	Microglia_SHAM_vs_Microglia_OGD	2.338916043	2.649046727
Tmem214	8.58E-07	0.0185	Microglia_SHAM_vs_Microglia_OGD	0.112907326	0.181149601
Cybc1	8.49E-07	0.0183	Microglia_SHAM_vs_Microglia_OGD	0.971445688	1.12190251
Setd7	8.40E-07	0.0181	Microglia_SHAM_vs_Microglia_OGD	0.2318182	0.298054204
Rpl18a	8.24E-07	0.01776	Microglia_SHAM_vs_Microglia_OGD	7.958971132	8.82905324
Mcts1	8.22E-07	0.01772	Microglia_SHAM_vs_Microglia_OGD	0.448128156	0.565098157
Bcor	8.21E-07	0.01771	Microglia_SHAM_vs_Microglia_OGD	0.553785796	0.686821828
Pfn1	8.15E-07	0.01758	Microglia_SHAM_vs_Microglia_OGD	20.92497381	22.02677984
Vps13c	8.10E-07	0.01747	Microglia_SHAM_vs_Microglia_OGD	0.686792976	0.786849789
Tmem222	8.10E-07	0.01746	Microglia_SHAM_vs_Microglia_OGD	0.243135371	0.339597671
Ergic3	8.05E-07	0.01737	Microglia_SHAM_vs_Microglia_OGD	0.577762567	0.695444289
Gpr89b	8.04E-07	0.01733	Microglia_SHAM_vs_Microglia_OGD	0.145265321	0.208549684
Cript	8.02E-07	0.01729	Microglia_SHAM_vs_Microglia_OGD	0.362562911	0.470717677
Por	7.98E-07	0.01721	Microglia_SHAM_vs_Microglia_OGD	0.624390645	0.411907809
Creg1	7.80E-07	0.01681	Microglia_SHAM_vs_Microglia_OGD	2.783648946	3.167658644
Usf1	7.77E-07	0.01675	Microglia_SHAM_vs_Microglia_OGD	0.244654425	0.35137055
Irf2bp2	7.59E-07	0.01636	Microglia_SHAM_vs_Microglia_OGD	0.055957436	0.105137364
Gsr	7.48E-07	0.01613	Microglia_SHAM_vs_Microglia_OGD	0.297590813	0.412384739
Tmco1	7.39E-07	0.01593	Microglia_SHAM_vs_Microglia_OGD	1.591921836	1.327684387
Sppl3	7.38E-07	0.01591	Microglia_SHAM_vs_Microglia_OGD	0.593368884	0.380670807
Rab8a	7.36E-07	0.01586	Microglia_SHAM_vs_Microglia_OGD	0.88515481	1.041080853
Mrps7	7.30E-07	0.01575	Microglia_SHAM_vs_Microglia_OGD	0.282645015	0.390948146
Stx4	7.09E-07	0.01529	Microglia_SHAM_vs_Microglia_OGD	0.130812086	0.203894483
Mrpl27	7.00E-07	0.0151	Microglia_SHAM_vs_Microglia_OGD	0.514545027	0.649430752
Fbxo3	7.00E-07	0.01509	Microglia_SHAM_vs_Microglia_OGD	0.166621331	0.247252751
Gmppa	6.99E-07	0.01507	Microglia_SHAM_vs_Microglia_OGD	0.048213058	0.108111938
Zfp106	6.87E-07	0.01482	Microglia_SHAM_vs_Microglia_OGD	1.162660851	1.350507457
Sema4c	6.79E-07	0.01463	Microglia_SHAM_vs_Microglia_OGD	0.260622991	0.351605399
Cd6	6.78E-07	0.01462	Microglia_SHAM_vs_Microglia_OGD	0.232470965	0.349836617
Crls1	6.77E-07	0.01459	Microglia_SHAM_vs_Microglia_OGD	0.09106635	0.165132108
Kcnk13	6.74E-07	0.01453	Microglia_SHAM_vs_Microglia_OGD	0.508045446	0.618558521

Twist1	6.72E-07	0.01449	Microglia_SHAM_vs_Microglia_OGD	0.083473493	0.027341228
Nme6	6.64E-07	0.01431	Microglia_SHAM_vs_Microglia_OGD	0.030282658	0.077623909
Rnf41	6.62E-07	0.01428	Microglia_SHAM_vs_Microglia_OGD	0.286931971	0.366054469
Cers5	6.61E-07	0.01425	Microglia_SHAM_vs_Microglia_OGD	0.457324839	0.592888362
Gpi	6.55E-07	0.01411	Microglia_SHAM_vs_Microglia_OGD	0.73157643	0.908559745
Mark2	6.54E-07	0.0141	Microglia_SHAM_vs_Microglia_OGD	0.440432636	0.577269808
Col4a1	6.48E-07	0.01398	Microglia_SHAM_vs_Microglia_OGD	0.032979908	0.069536438
Cul3	6.47E-07	0.01395	Microglia_SHAM_vs_Microglia_OGD	0.648850925	0.475579917
R3hdm2	6.40E-07	0.01381	Microglia_SHAM_vs_Microglia_OGD	0.700776471	0.849116311
Ndufaf3	6.37E-07	0.01374	Microglia_SHAM_vs_Microglia_OGD	0.168735017	0.260888081
Gmppb	6.37E-07	0.01373	Microglia_SHAM_vs_Microglia_OGD	0.053257889	0.091665288
Ucn2	6.34E-07	0.01367	Microglia_SHAM_vs_Microglia_OGD	0.02433074	0.003391899
H1f5	6.32E-07	0.01362	Microglia_SHAM_vs_Microglia_OGD	0.154300151	0.249032043
Nrip1	6.23E-07	0.01344	Microglia_SHAM_vs_Microglia_OGD	0.19516053	0.281724275
Ddost	6.13E-07	0.01321	Microglia_SHAM_vs_Microglia_OGD	0.960756719	1.184352395
Ryk	6.11E-07	0.01317	Microglia_SHAM_vs_Microglia_OGD	0.118297585	0.051784242
Gprasp2	6.04E-07	0.01302	Microglia_SHAM_vs_Microglia_OGD	0.016749404	0.054088825
Rpl3	5.97E-07	0.01287	Microglia_SHAM_vs_Microglia_OGD	6.178206509	6.819790061
Adh5	5.95E-07	0.01284	Microglia_SHAM_vs_Microglia_OGD	0.268943324	0.393770957
Pik3r2	5.96E-07	0.01284	Microglia_SHAM_vs_Microglia_OGD	0.04371083	0.100129342
Fam3c	5.86E-07	0.01263	Microglia_SHAM_vs_Microglia_OGD	0.347291882	0.458902727
Itpkb	5.84E-07	0.0126	Microglia_SHAM_vs_Microglia_OGD	1.869826603	1.471214925
Ptpn18	5.83E-07	0.01257	Microglia_SHAM_vs_Microglia_OGD	0.328148863	0.437757523
Crbn	5.78E-07	0.01246	Microglia_SHAM_vs_Microglia_OGD	0.180901251	0.26086406
Stx7	5.75E-07	0.01239	Microglia_SHAM_vs_Microglia_OGD	1.651381402	1.90739341
Eloa	5.72E-07	0.01233	Microglia_SHAM_vs_Microglia_OGD	0.682696033	0.828334811
Pgm1	5.31E-07	0.01145	Microglia_SHAM_vs_Microglia_OGD	0.05653632	0.105092313
Rnf187	5.27E-07	0.01137	Microglia_SHAM_vs_Microglia_OGD	0.259316412	0.346665884
Ccdc102a	5.26E-07	0.01134	Microglia_SHAM_vs_Microglia_OGD	0.086033382	0.149751617
Rnase11	5.21E-07	0.01123	Microglia_SHAM_vs_Microglia_OGD	0.000328623	0.026531222
Maf1	5.18E-07	0.01117	Microglia_SHAM_vs_Microglia_OGD	0.404629347	0.527491169
Gnptg	5.16E-07	0.01113	Microglia_SHAM_vs_Microglia_OGD	0.201795593	0.309542048
Samm50	5.09E-07	0.01097	Microglia_SHAM_vs_Microglia_OGD	0.167467459	0.257132335
Fez1	5.05E-07	0.0109	Microglia_SHAM_vs_Microglia_OGD	0.305415897	0.436437629
Edem2	4.91E-07	0.01058	Microglia_SHAM_vs_Microglia_OGD	0.796710482	0.953443263
Pam16	4.91E-07	0.01058	Microglia_SHAM_vs_Microglia_OGD	0.334247417	0.449021061
Ampd1	4.90E-07	0.01055	Microglia_SHAM_vs_Microglia_OGD	0.240326986	0.128058037
Tmem59	4.87E-07	0.0105	Microglia_SHAM_vs_Microglia_OGD	2.401657377	2.668694311
Tmf1	4.86E-07	0.01048	Microglia_SHAM_vs_Microglia_OGD	0.53359701	0.729030234
Med13	4.83E-07	0.01041	Microglia_SHAM_vs_Microglia_OGD	0.910416446	0.672635429
Rps27a.1	4.82E-07	0.01039	Microglia_SHAM_vs_Microglia_OGD	9.464439827	10.29973165
Cers2	4.79E-07	0.01033	Microglia_SHAM_vs_Microglia_OGD	0.762027234	0.979023013
Ppp6r1	4.67E-07	0.01006	Microglia_SHAM_vs_Microglia_OGD	0.19030419	0.267030354
Pyroxd2	4.64E-07	0.01001	Microglia_SHAM_vs_Microglia_OGD	0.060394542	0.113785808
Txndc9	4.60E-07	0.009916	Microglia_SHAM_vs_Microglia_OGD	0.69564417	0.837186884
Ap2s1	4.59E-07	0.00989	Microglia_SHAM_vs_Microglia_OGD	0.629947984	0.784164724
Erg28	4.57E-07	0.00986	Microglia_SHAM_vs_Microglia_OGD	0.314963141	0.417542038
Lrrc42	4.44E-07	0.009582	Microglia_SHAM_vs_Microglia_OGD	0.277077091	0.394775932
Fam104a	4.36E-07	0.009399	Microglia_SHAM_vs_Microglia_OGD	0.466426832	0.579416268
P2ry14	4.35E-07	0.009376	Microglia_SHAM_vs_Microglia_OGD	0.314952472	0.171998294
Exosc9	4.34E-07	0.009347	Microglia_SHAM_vs_Microglia_OGD	0.193904245	0.298786194
Zfp91	4.33E-07	0.009334	Microglia_SHAM_vs_Microglia_OGD	2.194483889	1.772991608
Tmem171	4.33E-07	0.009328	Microglia_SHAM_vs_Microglia_OGD	0.085097012	0.157873931
Ndufv1	4.32E-07	0.009313	Microglia_SHAM_vs_Microglia_OGD	0.300398663	0.390076531
Cyba	4.30E-07	0.009265	Microglia_SHAM_vs_Microglia_OGD	5.313922472	5.969910844
Isg20l2	4.27E-07	0.009208	Microglia_SHAM_vs_Microglia_OGD	0.057175961	0.114437176
Med25	4.25E-07	0.009163	Microglia_SHAM_vs_Microglia_OGD	0.065420555	0.126500733
Tpst2	4.22E-07	0.009097	Microglia_SHAM_vs_Microglia_OGD	0.207660745	0.301726
Aplp1	4.13E-07	0.008908	Microglia_SHAM_vs_Microglia_OGD	1.476918493	1.781942089
Rnf216	4.08E-07	0.008802	Microglia_SHAM_vs_Microglia_OGD	0.494467629	0.600156388
Btbd11	4.08E-07	0.008791	Microglia_SHAM_vs_Microglia_OGD	0.401964382	0.176681041
Acsf2	4.04E-07	0.008712	Microglia_SHAM_vs_Microglia_OGD	0.413962948	0.54939372
Evl	4.03E-07	0.008685	Microglia_SHAM_vs_Microglia_OGD	0.716240631	0.863923401
Bcl2l14	3.99E-07	0.008607	Microglia_SHAM_vs_Microglia_OGD	0.025350038	0.063188792
Cul4b	3.99E-07	0.008597	Microglia_SHAM_vs_Microglia_OGD	0.195503528	0.285738995
Rae1	3.98E-07	0.008573	Microglia_SHAM_vs_Microglia_OGD	0.297019129	0.369419426
Atp5f1d	3.96E-07	0.008548	Microglia_SHAM_vs_Microglia_OGD	1.04657814	1.249324922
Jtb	3.95E-07	0.008517	Microglia_SHAM_vs_Microglia_OGD	0.403867831	0.547871953
Mex3c	3.94E-07	0.008489	Microglia_SHAM_vs_Microglia_OGD	1.030995964	0.788725491
Dda1	3.94E-07	0.008487	Microglia_SHAM_vs_Microglia_OGD	0.455464595	0.572674594
Gstp1	3.86E-07	0.008331	Microglia_SHAM_vs_Microglia_OGD	1.253771855	1.552988479
Khdrbs1	3.84E-07	0.008284	Microglia_SHAM_vs_Microglia_OGD	1.077028648	1.247199526
Arl5a	3.84E-07	0.008269	Microglia_SHAM_vs_Microglia_OGD	0.124285645	0.204153922
Cdkn2aip	3.77E-07	0.008132	Microglia_SHAM_vs_Microglia_OGD	0.064300486	0.109029872
Ppp1r15b	3.72E-07	0.00803	Microglia_SHAM_vs_Microglia_OGD	0.286448762	0.403839289
Dnajb14	3.69E-07	0.00796	Microglia_SHAM_vs_Microglia_OGD	1.924777466	2.219867266
Fam220a	3.64E-07	0.007852	Microglia_SHAM_vs_Microglia_OGD	0.089391245	0.14993912
Myo9b	3.62E-07	0.0078	Microglia_SHAM_vs_Microglia_OGD	1.930494829	1.542537577
Ppp1cc	3.53E-07	0.007618	Microglia_SHAM_vs_Microglia_OGD	1.170792377	1.354123915
Unc50	3.49E-07	0.007532	Microglia_SHAM_vs_Microglia_OGD	0.148109127	0.21769307
Abraxas2	3.48E-07	0.007505	Microglia_SHAM_vs_Microglia_OGD	0.447539957	0.544767056
Nme2	3.48E-07	0.007498	Microglia_SHAM_vs_Microglia_OGD	1.60344536	1.909805606
Paics	3.48E-07	0.007493	Microglia_SHAM_vs_Microglia_OGD	0.253869755	0.382540544
Phf20l1.1	3.47E-07	0.007492	Microglia_SHAM_vs_Microglia_OGD	0.398979667	0.524904794
Fastk	3.44E-07	0.007416	Microglia_SHAM_vs_Microglia_OGD	0.191457415	0.269161159
Srpk1	3.41E-07	0.007361	Microglia_SHAM_vs_Microglia_OGD	0.289345201	0.394307226

Ssbp4	3.38E-07	0.007291	Microglia_SHAM_vs_Microglia_OGD	0.219972141	0.29161835
Faf1	3.32E-07	0.007168	Microglia_SHAM_vs_Microglia_OGD	0.179922844	0.255041216
Rab39a	3.32E-07	0.007148	Microglia_SHAM_vs_Microglia_OGD	0.083167391	0.151881172
Focad	3.31E-07	0.00713	Microglia_SHAM_vs_Microglia_OGD	0.2500567	0.357357157
Piezo1	3.27E-07	0.007042	Microglia_SHAM_vs_Microglia_OGD	4.724882694	4.014276582
Cdk6	3.26E-07	0.007022	Microglia_SHAM_vs_Microglia_OGD	2.306936867	1.867054323
Drg1	3.26E-07	0.007019	Microglia_SHAM_vs_Microglia_OGD	0.195582917	0.270870706
Slc9a1	3.25E-07	0.007013	Microglia_SHAM_vs_Microglia_OGD	0.230609653	0.128225672
Pygb	3.24E-07	0.006996	Microglia_SHAM_vs_Microglia_OGD	0.212624306	0.300067838
Anapc7	3.20E-07	0.006905	Microglia_SHAM_vs_Microglia_OGD	0.158579326	0.218860374
Zfp532	3.18E-07	0.006849	Microglia_SHAM_vs_Microglia_OGD	0.089409531	0.156698374
Polr2d	3.10E-07	0.00668	Microglia_SHAM_vs_Microglia_OGD	1.090727613	1.26173707
Tm9sf4	3.10E-07 0.006677		Microglia_SHAM_vs_Microglia_OGD	0.470774329	0.615910433
Uqcrc1	3.09E-07	0.00666	Microglia_SHAM_vs_Microglia_OGD	0.6116457	0.769296994
Cxcl10	3.01E-07	0.006495	Microglia_SHAM_vs_Microglia_OGD	3.255252522	2.78649432
Whrn	3.01E-07	0.006492	Microglia_SHAM_vs_Microglia_OGD	0.622581612	0.762154499
Cd99	3.00E-07	0.006469	Microglia_SHAM_vs_Microglia_OGD	0.392384369	0.523669811
Tmem179b	2.99E-07	0.006442	Microglia_SHAM_vs_Microglia_OGD	1.015849514	1.189357484
Psmc5	2.97E-07	0.006398	Microglia_SHAM_vs_Microglia_OGD	0.427859627	0.550967112
Rasgrp3	2.96E-07	0.006381	Microglia_SHAM_vs_Microglia_OGD	1.774107431	2.001144529
Anapc16	2.93E-07	0.006318	Microglia_SHAM_vs_Microglia_OGD	0.277635031	0.373934554
Mrps18a	2.89E-07	0.006233	Microglia_SHAM_vs_Microglia_OGD	0.242230553	0.329146716
Mfsd13a	2.88E-07	0.006215	Microglia_SHAM_vs_Microglia_OGD	0.074171916	0.13033329
Cox8b	2.83E-07	0.006102	Microglia_SHAM_vs_Microglia_OGD	0.000328623	0.027525524
Fgr	2.81E-07	0.006068	Microglia_SHAM_vs_Microglia_OGD	0.229774847	0.30131367
Mrps16	2.80E-07	0.006047	Microglia_SHAM_vs_Microglia_OGD	0.240788874	0.348527723
Ppil2	2.79E-07	0.006016	Microglia_SHAM_vs_Microglia_OGD	0.266390926	0.359164273
Kidins220	2.76E-07	0.005948	Microglia_SHAM_vs_Microglia_OGD	0.270028561	0.384023408
Csrp1	2.75E-07	0.005921	Microglia_SHAM_vs_Microglia_OGD	0.63322319	0.812354135
Tram2	2.75E-07	0.005921	Microglia_SHAM_vs_Microglia_OGD	0.066190323	0.118806179
Psmg2	2.74E-07	0.005908	Microglia_SHAM_vs_Microglia_OGD	0.096990095	0.1638562
Fgd4	2.72E-07	0.005867	Microglia_SHAM_vs_Microglia_OGD	0.134531074	0.224102385
Ttc3	2.72E-07	0.005866	Microglia_SHAM_vs_Microglia_OGD	0.598530107	0.71859206
Gpat4	2.71E-07	0.005848	Microglia_SHAM_vs_Microglia_OGD	0.152220662	0.233607471
Polr2j	2.68E-07	0.005768	Microglia_SHAM_vs_Microglia_OGD	0.33688443	0.42984885
Fes	2.63E-07	0.005665	Microglia_SHAM_vs_Microglia_OGD	0.044807172	0.101403465
Flt3lg	2.62E-07	0.005649	Microglia_SHAM_vs_Microglia_OGD	0.027932043	0.070787131
Fibp	2.62E-07	0.005646	Microglia_SHAM_vs_Microglia_OGD	0.247223908	0.329076951
Eng	2.61E-07	0.00563	Microglia_SHAM_vs_Microglia_OGD	0.509198267	0.661701497
Alpk2	2.59E-07	0.00559	Microglia_SHAM_vs_Microglia_OGD	0.141164185	0.066255191
Nfrkb	2.55E-07	0.005487	Microglia_SHAM_vs_Microglia_OGD	0.300847157	0.394181943
Pfkl	2.53E-07	0.005461	Microglia_SHAM_vs_Microglia_OGD	0.342512661	0.444836121
Cpeb2	2.53E-07	0.005458	Microglia_SHAM_vs_Microglia_OGD	0.198092719	0.101948859
Zc3h8	2.51E-07	0.005408	Microglia_SHAM_vs_Microglia_OGD	0.109011021	0.182767044
Hdgfl2	2.47E-07	0.005335	Microglia_SHAM_vs_Microglia_OGD	0.242880883	0.34906372
Bsg	2.45E-07	0.005282	Microglia_SHAM_vs_Microglia_OGD	2.922898924	3.381206118
Pcbd2	2.42E-07	0.005221	Microglia_SHAM_vs_Microglia_OGD	0.250382121	0.334342439
Mpg	2.42E-07	0.00521	Microglia_SHAM_vs_Microglia_OGD	0.053303238	0.111896417
Nbeal1	2.41E-07 0.005203		Microglia_SHAM_vs_Microglia_OGD	0.122745934	0.188746121
Rpl41	2.39E-07	0.00515	Microglia_SHAM_vs_Microglia_OGD	17.06089382	18.3479606
Fcho2	2.37E-07	0.005114	Microglia_SHAM_vs_Microglia_OGD	0.894770961	1.010261122
Nt5c	2.33E-07	0.005026	Microglia_SHAM_vs_Microglia_OGD	0.068747903	0.136260599
Rbm18	2.33E-07	0.005026	Microglia_SHAM_vs_Microglia_OGD	0.328473131	0.437149327
Smarca4	2.30E-07	0.004957	Microglia_SHAM_vs_Microglia_OGD	0.626199753	0.803355011
Arhgap30	2.28E-07	0.004914	Microglia_SHAM_vs_Microglia_OGD	0.222249524	0.320569945
Itm2a	2.27E-07	0.004896	Microglia_SHAM_vs_Microglia_OGD	0.183475575	0.296233348
Cct4	2.26E-07	0.00487	Microglia_SHAM_vs_Microglia_OGD	1.448192582	1.68726358
Rnpepl1	2.26E-07	0.004868	Microglia_SHAM_vs_Microglia_OGD	0.149607955	0.246583882
Nsd1	2.23E-07	0.004797	Microglia_SHAM_vs_Microglia_OGD	0.541093266	0.662622343
Calcrl	2.18E-07	0.004692	Microglia_SHAM_vs_Microglia_OGD	2.405305591	1.924042845
Btk	2.17E-07	0.004675	Microglia_SHAM_vs_Microglia_OGD	0.114129558	0.18470151
Mmrn2	2.14E-07	0.004605	Microglia_SHAM_vs_Microglia_OGD	0.05135589	0.104694478
Strn4	2.13E-07	0.004583	Microglia_SHAM_vs_Microglia_OGD	0.188725824	0.268775818
Ilkap	2.11E-07	0.004559	Microglia_SHAM_vs_Microglia_OGD	0.349945055	0.445079118
Ak3	2.09E-07	0.004512	Microglia_SHAM_vs_Microglia_OGD	0.238412204	0.323303386
Ufsp2	2.09E-07	0.004504	Microglia_SHAM_vs_Microglia_OGD	0.248805655	0.372027239
Lactb	2.04E-07	0.004406	Microglia_SHAM_vs_Microglia_OGD	0.395632389	0.50833879
Tmem230	1.99E-07	0.004298	Microglia_SHAM_vs_Microglia_OGD	0.40024027	0.546195901
Actr1b	1.99E-07	0.004286	Microglia_SHAM_vs_Microglia_OGD	0.184255664	0.258935254
Asnsd1	1.96E-07	0.004219	Microglia_SHAM_vs_Microglia_OGD	0.087368064	0.154297559
Hmbs	1.96E-07	0.004217	Microglia_SHAM_vs_Microglia_OGD	0.093222071	0.16057025
Hcfc1r1	1.95E-07	0.004194	Microglia_SHAM_vs_Microglia_OGD	0.020661792	0.067825933
Fus	1.93E-07	0.004164	Microglia_SHAM_vs_Microglia_OGD	5.335465289	5.86494255
Tbc1d15	1.91E-07	0.004117	Microglia_SHAM_vs_Microglia_OGD	0.437212618	0.56068024
Mrpl40	1.90E-07	0.004098	Microglia_SHAM_vs_Microglia_OGD	0.103975701	0.180947678
Slc7a7	1.79E-07	0.003849	Microglia_SHAM_vs_Microglia_OGD	0.173441984	0.275528132
Syngr2	1.78E-07	0.003834	Microglia_SHAM_vs_Microglia_OGD	0.101519584	0.17533009
Sh2d3c	1.76E-07	0.003793	Microglia_SHAM_vs_Microglia_OGD	0.053162612	0.103750961
Irs2	1.74E-07	0.003753	Microglia_SHAM_vs_Microglia_OGD	0.305967961	0.172546972
Rhbdf1	1.73E-07	0.003722	Microglia_SHAM_vs_Microglia_OGD	0.01843721	0.060587638
Socs6	1.72E-07	0.003716	Microglia_SHAM_vs_Microglia_OGD	0.259463122	0.338267451
Zranb2	1.72E-07	0.003714	Microglia_SHAM_vs_Microglia_OGD	1.118971236	1.308536014
Pstk	1.71E-07	0.00368	Microglia_SHAM_vs_Microglia_OGD	0.163656934	0.230647523
Tram1	1.71E-07	0.003676	Microglia_SHAM_vs_Microglia_OGD	1.848342046	2.139368809
Ivns1abp	1.70E-07	0.003663	Microglia_SHAM_vs_Microglia_OGD	3.10458986	3.568644181
Mettl3	1.69E-07	0.003651	Microglia_SHAM_vs_Microglia_OGD	0.138233869	0.222921316

Cast	1.69E-07	0.003642	Microglia_SHAM_vs_Microglia_OGD	1.863179825	2.127204424
Timm50	1.69E-07	0.003641	Microglia_SHAM_vs_Microglia_OGD	0.107718905	0.178430571
Atp23	1.66E-07	0.003587	Microglia_SHAM_vs_Microglia_OGD	0.168207173	0.250068977
Zfand6	1.66E-07	0.003569	Microglia_SHAM_vs_Microglia_OGD	1.059689509	1.267918537
Tarbp2	1.65E-07	0.00355	Microglia_SHAM_vs_Microglia_OGD	0.058588392	0.113556586
Map3k14	1.64E-07	0.003546	Microglia_SHAM_vs_Microglia_OGD	0.701864339	0.86427565
RGD1305350	1.64E-07	0.003531	Microglia_SHAM_vs_Microglia_OGD	0.153603252	0.232329972
Map3k11	1.60E-07	0.003455	Microglia_SHAM_vs_Microglia_OGD	0.213538719	0.31796926
Ube2d1	1.59E-07	0.003418	Microglia_SHAM_vs_Microglia_OGD	0.527195292	0.67118683
Cul2	1.57E-07	0.003393	Microglia_SHAM_vs_Microglia_OGD	0.267760205	0.376464367
Fmnl2	1.54E-07	0.003316	Microglia_SHAM_vs_Microglia_OGD	0.366417388	0.473742552
Ext2	1.53E-07	0.003308	Microglia_SHAM_vs_Microglia_OGD	0.257875566	0.344994544
Ktn1	1.52E-07	0.003282	Microglia_SHAM_vs_Microglia_OGD	2.44847518	1.990049237
Rufy3	1.52E-07	0.003271	Microglia_SHAM_vs_Microglia_OGD	0.279665697	0.382589881
Cgrrf1	1.50E-07	0.00324	Microglia_SHAM_vs_Microglia_OGD	0.283984374	0.389434108
Dguok	1.47E-07	0.003161	Microglia_SHAM_vs_Microglia_OGD	0.195407695	0.27292966
Tmub2	1.44E-07	0.003094	Microglia_SHAM_vs_Microglia_OGD	0.138832881	0.215901038
Pcnp	1.43E-07	0.003093	Microglia_SHAM_vs_Microglia_OGD	0.171750293	0.267477079
Atp6v1b2	1.40E-07	0.003026	Microglia_SHAM_vs_Microglia_OGD	1.046511464	0.721258437
Kif3b	1.40E-07	0.003025	Microglia_SHAM_vs_Microglia_OGD	0.0882055	0.162544843
Gxylt1	1.40E-07	0.003017	Microglia_SHAM_vs_Microglia_OGD	0.462351072	0.587867427
Slain2	1.40E-07	0.003012	Microglia_SHAM_vs_Microglia_OGD	0.447379188	0.598204403
Slamf9	1.39E-07	0.002992	Microglia_SHAM_vs_Microglia_OGD	0.024068291	0.084993699
Zmpste24	1.39E-07	0.002988	Microglia_SHAM_vs_Microglia_OGD	0.437621182	0.568236401
Zmym5	1.37E-07	0.002946	Microglia_SHAM_vs_Microglia_OGD	0.5921195	0.722959253
Rn7sl1	1.35E-07	0.002918	Microglia_SHAM_vs_Microglia_OGD	0.042582303	0.006066434
Cotl1	1.35E-07	0.002912	Microglia_SHAM_vs_Microglia_OGD	3.688838407	4.308793692
Rbm7	1.34E-07	0.00288	Microglia_SHAM_vs_Microglia_OGD	0.708092383	0.852875032
Rbmxl1	1.34E-07	0.00288	Microglia_SHAM_vs_Microglia_OGD	0.603890975	0.757714462
Plxdc1	1.32E-07	0.002847	Microglia_SHAM_vs_Microglia_OGD	0.066912944	0.019576729
Mboat7l1	1.32E-07	0.002834	Microglia_SHAM_vs_Microglia_OGD	0.693280231	0.816580112
Tpra1	1.31E-07	0.002832	Microglia_SHAM_vs_Microglia_OGD	0.170591484	0.251829572
Malsu1	1.27E-07	0.002748	Microglia_SHAM_vs_Microglia_OGD	0.128383255	0.223470066
Rab12	1.27E-07	0.002746	Microglia_SHAM_vs_Microglia_OGD	0.49154296	0.29379825
Pld2	1.27E-07	0.002735	Microglia_SHAM_vs_Microglia_OGD	0.092415512	0.159694247
Taf4b	1.27E-07	0.002728	Microglia_SHAM_vs_Microglia_OGD	0.148492018	0.060908929
B4galt7	1.25E-07	0.002686	Microglia_SHAM_vs_Microglia_OGD	0.028367587	0.088080732
Rpl15	1.24E-07	0.002682	Microglia_SHAM_vs_Microglia_OGD	7.18788335	6.464076974
Afdn	1.23E-07	0.002646	Microglia_SHAM_vs_Microglia_OGD	0.322298742	0.434381625
Slc30a1	1.20E-07	0.002584	Microglia_SHAM_vs_Microglia_OGD	0.624927669	0.451412876
Pura	1.19E-07	0.002565	Microglia_SHAM_vs_Microglia_OGD	0.150568651	0.24315313
Gemin7	1.15E-07	0.00247	Microglia_SHAM_vs_Microglia_OGD	0.135905735	0.222405913
Nqo2	1.13E-07 0.002446		Microglia_SHAM_vs_Microglia_OGD	0.000328623	0.027699066
Hnrnpa2b1	1.13E-07	0.00243	Microglia_SHAM_vs_Microglia_OGD	3.478427598	4.028366537
Ahnak	1.11E-07	0.0024	Microglia_SHAM_vs_Microglia_OGD	0.153033499	0.075606892
Cox4i1	1.11E-07	0.0024	Microglia_SHAM_vs_Microglia_OGD	3.502676766	3.914291467
Nans	1.10E-07	0.002378	Microglia_SHAM_vs_Microglia_OGD	0.141188284	0.23017422
Ube2d2	1.10E-07	0.002362	Microglia_SHAM_vs_Microglia_OGD	1.704822944	1.978008855
Ctps2	1.08E-07	0.002338	Microglia_SHAM_vs_Microglia_OGD	0.265974965	0.356820749
Srp9	1.08E-07	0.002327	Microglia_SHAM_vs_Microglia_OGD	0.508667442	0.648270111
Fam210b	1.08E-07	0.002319	Microglia_SHAM_vs_Microglia_OGD	0.182915594	0.26480556
Scamp4	1.06E-07	0.002293	Microglia_SHAM_vs_Microglia_OGD	0.209191319	0.311366906
Angptl4	1.06E-07	0.002288	Microglia_SHAM_vs_Microglia_OGD	0.031063682	0.074411761
B4galt1	1.05E-07	0.002268	Microglia_SHAM_vs_Microglia_OGD	0.233998298	0.323894993
Dera	1.03E-07	0.002212	Microglia_SHAM_vs_Microglia_OGD	0.574105652	0.724042138
Mdfi	1.01E-07	0.002173	Microglia_SHAM_vs_Microglia_OGD	0.080672321	0.149185089
Prr7	1.01E-07	0.002168	Microglia_SHAM_vs_Microglia_OGD	0.124846038	0.215447945
Rufy1	9.90E-08	0.002134	Microglia_SHAM_vs_Microglia_OGD	0.501790424	0.657213116
Ikbip	9.84E-08	0.002122	Microglia_SHAM_vs_Microglia_OGD	0.277970833	0.369054015
Meaf6	9.82E-08	0.002117	Microglia_SHAM_vs_Microglia_OGD	0.20360146	0.302313221
RGD1560821	9.82E-08	0.002117	Microglia_SHAM_vs_Microglia_OGD	0.004533333	0.038048415
Atad2b	9.80E-08	0.002114	Microglia_SHAM_vs_Microglia_OGD	0.84664661	0.998501022
Rrp1	9.62E-08	0.002074	Microglia_SHAM_vs_Microglia_OGD	0.170936434	0.262315513
Hormad2	9.50E-08	0.002048	Microglia_SHAM_vs_Microglia_OGD	0.092943215	0.035605271
Setx	9.45E-08	0.002038	Microglia_SHAM_vs_Microglia_OGD	0.356559656	0.485112433
Ndufaf6	9.30E-08	0.002004	Microglia_SHAM_vs_Microglia_OGD	0.083554273	0.156800694
Csf3r	9.29E-08	0.002003	Microglia_SHAM_vs_Microglia_OGD	0.172345564	0.279617431
Ss18l2	9.17E-08	0.001977	Microglia_SHAM_vs_Microglia_OGD	0.492376395	0.617311161
Rab35	9.15E-08	0.001972	Microglia_SHAM_vs_Microglia_OGD	0.180219147	0.271648425
Ptges3	9.05E-08	0.001952	Microglia_SHAM_vs_Microglia_OGD	2.780413585	3.180210567
Ap2b1	9.04E-08	0.001948	Microglia_SHAM_vs_Microglia_OGD	0.919495447	0.689061067
Stam2	8.99E-08	0.001939	Microglia_SHAM_vs_Microglia_OGD	0.185150206	0.273318035
Lef1	8.90E-08	0.001919	Microglia_SHAM_vs_Microglia_OGD	0.385229412	0.230702088
Zbtb43	8.86E-08	0.001911	Microglia_SHAM_vs_Microglia_OGD	0.069083798	0.124810136
Tgif2	8.84E-08	0.001907	Microglia_SHAM_vs_Microglia_OGD	0.389810552	0.499559492
Vps11	8.79E-08	0.001894	Microglia_SHAM_vs_Microglia_OGD	0.216992044	0.308424723
Kat8	8.76E-08	0.001889	Microglia_SHAM_vs_Microglia_OGD	0.196515502	0.30368967
Il10ra	8.67E-08	0.001869	Microglia_SHAM_vs_Microglia_OGD	0.362559041	0.488925011
Isyna1	8.59E-08	0.001852	Microglia_SHAM_vs_Microglia_OGD	0.179933714	0.280473662
Cped1	8.58E-08	0.001849	Microglia_SHAM_vs_Microglia_OGD	0.93765354	0.662293119
Atg3	8.55E-08	0.001844	Microglia_SHAM_vs_Microglia_OGD	0.388381183	0.507332798
Zfx	8.55E-08	0.001844	Microglia_SHAM_vs_Microglia_OGD	0.339719657	0.441610222
Gsdmd	8.53E-08	0.00184	Microglia_SHAM_vs_Microglia_OGD	0.114151652	0.179105994
Tdg	8.49E-08	0.001831	Microglia_SHAM_vs_Microglia_OGD	0.478369221	0.599591505
Trem2	8.47E-08	0.001827	Microglia_SHAM_vs_Microglia_OGD	8.298654253	9.092086415
Tbc1d10a	8.46E-08	0.001825	Microglia_SHAM_vs_Microglia_OGD	0.202836272	0.277329309

Timm13	8.39E-08	0.00181	Microglia_SHAM_vs_Microglia_OGD	0.784827033	0.925979431
Trnau1ap	8.38E-08	0.001808	Microglia_SHAM_vs_Microglia_OGD	0.080527769	0.146483275
Glycam1	8.37E-08	0.001804	Microglia_SHAM_vs_Microglia_OGD	0.000328623	0.04287615
Ubtd2	8.19E-08	0.001767	Microglia_SHAM_vs_Microglia_OGD	0.149326315	0.070054153
Racgap1	8.00E-08	0.001725	Microglia_SHAM_vs_Microglia_OGD	0.04346096	0.011961719
Srf	7.96E-08	0.001717	Microglia_SHAM_vs_Microglia_OGD	0.11453619	0.202341801
Pex16	7.90E-08	0.001704	Microglia_SHAM_vs_Microglia_OGD	0.09284599	0.154800094
Ndufb8	7.74E-08	0.001669	Microglia_SHAM_vs_Microglia_OGD	1.137744369	1.350249977
Dhrs3	7.57E-08	0.001633	Microglia_SHAM_vs_Microglia_OGD	0.065576918	0.113240655
Ubac1	7.55E-08	0.001627	Microglia_SHAM_vs_Microglia_OGD	0.057175015	0.119552041
Ostm1	7.40E-08	0.001596	Microglia_SHAM_vs_Microglia_OGD	0.430754084	0.560751583
Ndufb7	7.37E-08	0.001589	Microglia_SHAM_vs_Microglia_OGD	0.68181501	0.880810905
Adgrl3	7.36E-08	0.001586	Microglia_SHAM_vs_Microglia_OGD	0.111708776	0.055433104
Rxrb	7.35E-08	0.001585	Microglia_SHAM_vs_Microglia_OGD	0.2026866	0.304006147
Arfgap2	7.33E-08	0.001581	Microglia_SHAM_vs_Microglia_OGD	0.308593556	0.4110138
Slc52a2	7.19E-08	0.00155	Microglia_SHAM_vs_Microglia_OGD	0.056995615	0.119859799
Unc119	7.17E-08	0.001546	Microglia_SHAM_vs_Microglia_OGD	0.160117054	0.228665484
Rfc1	7.16E-08	0.001545	Microglia_SHAM_vs_Microglia_OGD	0.567822749	0.696496866
Dclre1a	7.09E-08	0.00153	Microglia_SHAM_vs_Microglia_OGD	0.197802185	0.272708534
Rap2a	7.03E-08	0.001515	Microglia_SHAM_vs_Microglia_OGD	0.154044132	0.236094779
Rgl2	7.03E-08	0.001515	Microglia_SHAM_vs_Microglia_OGD	0.156522217	0.239490822
Arl4a	6.99E-08	0.001507	Microglia_SHAM_vs_Microglia_OGD	0.946364344	0.616564136
Eea1	6.97E-08	0.001502	Microglia_SHAM_vs_Microglia_OGD	1.059870173	0.760097966
Nt5c3b	6.97E-08	0.001502	Microglia_SHAM_vs_Microglia_OGD	0.102027191	0.164993982
Gps1	6.90E-08	0.001488	Microglia_SHAM_vs_Microglia_OGD	0.24547628	0.350694049
Faslg	6.75E-08	0.001454	Microglia_SHAM_vs_Microglia_OGD	0.032263079	0.00513451
Osbpl1a	6.73E-08	0.001451	Microglia_SHAM_vs_Microglia_OGD	3.192498325	3.580902179
Tpgs1	6.65E-08	0.001434	Microglia_SHAM_vs_Microglia_OGD	0.148885089	0.233359989
Krcc1	6.64E-08	0.001432	Microglia_SHAM_vs_Microglia_OGD	1.268449488	1.52644642
Npepl1	6.64E-08	0.001432	Microglia_SHAM_vs_Microglia_OGD	0.081046052	0.134144989
Clip1	6.52E-08	0.001405	Microglia_SHAM_vs_Microglia_OGD	0.225119346	0.319618871
Tcn2	6.48E-08	0.001398	Microglia_SHAM_vs_Microglia_OGD	1.955551704	1.574742673
Adam10	6.23E-08	0.001344	Microglia_SHAM_vs_Microglia_OGD	0.636536611	0.79383794
Mitf	6.14E-08	0.001324	Microglia_SHAM_vs_Microglia_OGD	0.661788446	0.403147314
Atp5po	5.90E-08	0.001271	Microglia_SHAM_vs_Microglia_OGD	0.795843939	1.032371975
Daglb	5.89E-08	0.00127	Microglia_SHAM_vs_Microglia_OGD	1.14123068	1.323649585
Exosc4	5.86E-08	0.001263	Microglia_SHAM_vs_Microglia_OGD	0.190203335	0.278822556
P4ha1	5.73E-08	0.001236	Microglia_SHAM_vs_Microglia_OGD	1.888988111	2.155272131
Ripor1	5.72E-08	0.001234	Microglia_SHAM_vs_Microglia_OGD	0.062228005	0.132613785
Hk1	5.68E-08	0.001224	Microglia_SHAM_vs_Microglia_OGD	0.395255724	0.256539755
Cited2	5.67E-08	0.001223	Microglia_SHAM_vs_Microglia_OGD	7.883699236	7.09214317
Gkap1	5.62E-08	0.001212	Microglia_SHAM_vs_Microglia_OGD	0.413872436	0.269698388
Mgp	5.55E-08	0.001197	Microglia_SHAM_vs_Microglia_OGD	0.06033439	0.122863872
Bfar	5.54E-08	0.001195	Microglia_SHAM_vs_Microglia_OGD	0.304379499	0.406520684
Spns1	5.54E-08	0.001195	Microglia_SHAM_vs_Microglia_OGD	0.145030036	0.227293529
Tbc1d4	5.53E-08	0.001193	Microglia_SHAM_vs_Microglia_OGD	0.576608958	0.312552827
Cant1	5.49E-08	0.001185	Microglia_SHAM_vs_Microglia_OGD	0.084078175	0.166497942
Clta	5.50E-08	0.001185	Microglia_SHAM_vs_Microglia_OGD	7.846594372	8.45036734
Mterf3	5.49E-08	0.001183	Microglia_SHAM_vs_Microglia_OGD	0.156773571	0.254601051
Adap2	5.46E-08	0.001177	Microglia_SHAM_vs_Microglia_OGD	0.07162508	0.129189014
Rnf215	5.43E-08	0.001171	Microglia_SHAM_vs_Microglia_OGD	0.041525198	0.096554147
Sec23b	5.34E-08	0.001152	Microglia_SHAM_vs_Microglia_OGD	0.764296302	0.562415971
Ipo5	5.28E-08	0.001139	Microglia_SHAM_vs_Microglia_OGD	0.722830063	0.485308835
Nr2f6	5.21E-08	0.001124	Microglia_SHAM_vs_Microglia_OGD	0.059228966	0.116429409
Mef2c	5.14E-08	0.001109	Microglia_SHAM_vs_Microglia_OGD	15.88664188	14.18360262
Arhgap31	5.08E-08	0.001094	Microglia_SHAM_vs_Microglia_OGD	3.416322954	2.680026619
Poldip3	5.06E-08	0.001092	Microglia_SHAM_vs_Microglia_OGD	0.42333407	0.583010282
Galk1	5.04E-08	0.001087	Microglia_SHAM_vs_Microglia_OGD	0.113523576	0.20221414
Sugt1	5.01E-08	0.001081	Microglia_SHAM_vs_Microglia_OGD	0.543209894	0.707438537
Prkaca	4.94E-08	0.001066	Microglia_SHAM_vs_Microglia_OGD	0.182178507	0.273944262
Tob1	4.93E-08	0.001064	Microglia_SHAM_vs_Microglia_OGD	0.219587499	0.322475815
Kcnj13	4.93E-08	0.001063	Microglia_SHAM_vs_Microglia_OGD	0.00125302	0.041574899
Aebp2	4.92E-08	0.00106	Microglia_SHAM_vs_Microglia_OGD	1.728214053	1.358118784
Psmd13	4.89E-08	0.001055	Microglia_SHAM_vs_Microglia_OGD	0.548873461	0.709400325
Atp5mc1	4.86E-08	0.001047	Microglia_SHAM_vs_Microglia_OGD	1.625637872	1.945240864
Lpcat2	4.84E-08	0.001043	Microglia_SHAM_vs_Microglia_OGD	4.891768156	5.350733828
Uri1	4.82E-08	0.001039	Microglia_SHAM_vs_Microglia_OGD	0.268492776	0.394902033
Riox1	4.75E-08	0.001023	Microglia_SHAM_vs_Microglia_OGD	0.375615623	0.526333777
Fam207a	4.70E-08	0.001013	Microglia_SHAM_vs_Microglia_OGD	0.17142008	0.265634331
Ftl1	4.66E-08	0.001004	Microglia_SHAM_vs_Microglia_OGD	31.22846714	29.58495185
Atg4c	4.65E-08	0.001003	Microglia_SHAM_vs_Microglia_OGD	0.233701815	0.126687692
Man1b1	4.60E-08	0.0009921	Microglia_SHAM_vs_Microglia_OGD	0.202740645	0.288919112
Ist1	4.59E-08	0.0009907	Microglia_SHAM_vs_Microglia_OGD	0.767766635	0.530488385
Cxcl17	4.54E-08	0.0009779	Microglia_SHAM_vs_Microglia_OGD	0.151435098	0.066934632
Diablo	4.44E-08	0.0009563	Microglia_SHAM_vs_Microglia_OGD	0.077985518	0.161043093
Adprhl2	4.41E-08	0.0009511	Microglia_SHAM_vs_Microglia_OGD	0.135165787	0.223429852
Cd22	4.40E-08	0.0009492	Microglia_SHAM_vs_Microglia_OGD	0.063668376	0.017788432
Kdelr2	4.37E-08	0.0009425	Microglia_SHAM_vs_Microglia_OGD	0.225816993	0.309180649
Ybx1-ps3	4.25E-08	0.0009159	Microglia_SHAM_vs_Microglia_OGD	0.164356334	0.249294766
Il1rn	4.19E-08	0.0009037	Microglia_SHAM_vs_Microglia_OGD	0.183340099	0.064610417
Fam89b	4.11E-08	0.0008871	Microglia_SHAM_vs_Microglia_OGD	0.293058048	0.42571472
Cbx5	4.09E-08	0.000882	Microglia_SHAM_vs_Microglia_OGD	0.318066751	0.452387698
Faf2	3.99E-08	0.0008607	Microglia_SHAM_vs_Microglia_OGD	0.481899523	0.611646107
Rnf114	3.94E-08	0.0008499	Microglia_SHAM_vs_Microglia_OGD	0.249142355	0.355921278
Slc39a13	3.82E-08	0.0008228	Microglia_SHAM_vs_Microglia_OGD	0.06600182	0.12319561
Ncor2	3.74E-08	0.0008064	Microglia_SHAM_vs_Microglia_OGD	0.337238927	0.459040748

Dusp22	3.65E-08	0.0007877	Microglia_SHAM_vs_Microglia_OGD	0.232628379	0.339171891
Dnajc22	3.63E-08	0.0007831	Microglia_SHAM_vs_Microglia_OGD	0.095113883	0.177455836
Phip	3.62E-08	0.0007804	Microglia_SHAM_vs_Microglia_OGD	0.435436481	0.567521834
Ankh	3.60E-08	0.0007757	Microglia_SHAM_vs_Microglia_OGD	0.744131591	0.377629295
Nup62	3.48E-08	0.000751	Microglia_SHAM_vs_Microglia_OGD	0.251241214	0.369119475
Mrps5	3.45E-08	0.0007438	Microglia_SHAM_vs_Microglia_OGD	0.293068795	0.410060609
Calm2	3.41E-08	0.0007358	Microglia_SHAM_vs_Microglia_OGD	0.935557011	1.134760244
Mllt11	3.40E-08	0.0007333	Microglia_SHAM_vs_Microglia_OGD	0.093331661	0.163186693
Tspan5	3.30E-08	0.0007119	Microglia_SHAM_vs_Microglia_OGD	0.570066693	0.360617081
Id1	3.28E-08	0.0007081	Microglia_SHAM_vs_Microglia_OGD	0.308675176	0.429762858
Bag1	3.13E-08	0.0006742	Microglia_SHAM_vs_Microglia_OGD	0.728989426	0.929644116
Dcbld2	3.12E-08	0.0006736	Microglia_SHAM_vs_Microglia_OGD	0.089443445	0.029853988
Eef1b2	3.12E-08	0.0006716	Microglia_SHAM_vs_Microglia_OGD	3.298633741	2.847733382
Supt16h	3.07E-08	0.0006627	Microglia_SHAM_vs_Microglia_OGD	0.402063249	0.522668293
RGD1311345	3.05E-08	0.0006571	Microglia_SHAM_vs_Microglia_OGD	0.183255139	0.265946893
Gpaa1	2.97E-08	0.0006402	Microglia_SHAM_vs_Microglia_OGD	0.138667084	0.231569989
Nsmaf	2.93E-08	0.0006325	Microglia_SHAM_vs_Microglia_OGD	0.910165105	0.628762668
Ndufb11	2.93E-08	0.0006317	Microglia_SHAM_vs_Microglia_OGD	1.336834682	1.589722728
Napg	2.92E-08	0.0006305	Microglia_SHAM_vs_Microglia_OGD	0.195066774	0.282415942
Smg5	2.77E-08	0.0005972	Microglia_SHAM_vs_Microglia_OGD	0.294875876	0.423821939
Prkcq	2.76E-08	0.0005953	Microglia_SHAM_vs_Microglia_OGD	1.324999507	1.639089597
Ssr1	2.76E-08	0.0005943	Microglia_SHAM_vs_Microglia_OGD	1.208771545	0.94074905
Snrpd3	2.71E-08	0.0005849	Microglia_SHAM_vs_Microglia_OGD	1.365077779	1.041104743
Laptm5	2.59E-08	0.0005576	Microglia_SHAM_vs_Microglia_OGD	7.705453763	8.457370117
Tpp2	2.59E-08	0.0005574	Microglia_SHAM_vs_Microglia_OGD	0.599028278	0.780878415
Ubqln1	2.54E-08	0.0005477	Microglia_SHAM_vs_Microglia_OGD	0.553178833	0.727168632
Mrpl58	2.51E-08	0.0005409	Microglia_SHAM_vs_Microglia_OGD	0.278409105	0.405367807
Hmgcr	2.45E-08	0.0005292	Microglia_SHAM_vs_Microglia_OGD	0.475049391	0.643741464
Mkrn1	2.44E-08	0.0005264	Microglia_SHAM_vs_Microglia_OGD	0.732155637	0.945199702
Alpk1	2.40E-08	0.0005182	Microglia_SHAM_vs_Microglia_OGD	0.994548667	1.277033034
Gpr132	2.40E-08	0.0005175	Microglia_SHAM_vs_Microglia_OGD	0.278034789	0.402424977
Sgpp1	2.35E-08	0.0005065	Microglia_SHAM_vs_Microglia_OGD	0.433090754	0.582479954
Gng10	2.34E-08	0.0005055	Microglia_SHAM_vs_Microglia_OGD	4.630335921	5.283825427
Gyg1	2.31E-08	0.0004979	Microglia_SHAM_vs_Microglia_OGD	0.125200396	0.225571699
Isca2	2.30E-08	0.0004949	Microglia_SHAM_vs_Microglia_OGD	0.453070516	0.603724254
Lonp2	2.29E-08	0.0004931	Microglia_SHAM_vs_Microglia_OGD	0.180482312	0.297691121
Derl1	2.26E-08	0.0004875	Microglia_SHAM_vs_Microglia_OGD	0.898215161	1.100577881
Cmklr1	2.24E-08	0.0004824	Microglia_SHAM_vs_Microglia_OGD	0.498758304	0.611471045
Mettl26	2.24E-08	0.0004823	Microglia_SHAM_vs_Microglia_OGD	0.066736386	0.154091987
Actr1a	2.23E-08	0.0004811	Microglia_SHAM_vs_Microglia_OGD	0.284969927	0.412452252
Asap1	2.19E-08	0.0004722	Microglia_SHAM_vs_Microglia_OGD	0.451492995	0.6022651
Smim10l1	2.18E-08	0.0004706	Microglia_SHAM_vs_Microglia_OGD	0.422032476	0.562670708
Use1	2.17E-08	0.0004683	Microglia_SHAM_vs_Microglia_OGD	0.613517465	0.788230819
Nat9	2.15E-08	0.000464	Microglia_SHAM_vs_Microglia_OGD	0.098958903	0.190943866
Fgfr1op2	2.10E-08	0.0004522	Microglia_SHAM_vs_Microglia_OGD	0.701876524	0.859768897
Ppcs	2.10E-08	0.0004521	Microglia_SHAM_vs_Microglia_OGD	0.04412902	0.098387365
Dbt	2.07E-08	0.0004457	Microglia_SHAM_vs_Microglia_OGD	0.281305297	0.396620073
Sirt7	2.06E-08	0.000445	Microglia_SHAM_vs_Microglia_OGD	0.32096795	0.434303108
Cdk5rap3	1.98E-08	0.0004277	Microglia_SHAM_vs_Microglia_OGD	0.186532101	0.280880833
Hsbp1	1.91E-08	0.0004123	Microglia_SHAM_vs_Microglia_OGD	0.534068925	0.722126243
Tmem115	1.90E-08	0.0004104	Microglia_SHAM_vs_Microglia_OGD	0.167805966	0.261656689
Zbtb10	1.89E-08	0.0004074	Microglia_SHAM_vs_Microglia_OGD	0.199984899	0.089125263
RT1-CE4	1.88E-08	0.0004054	Microglia_SHAM_vs_Microglia_OGD	7.855953611	8.538217418
Srfbp1	1.85E-08	0.0003994	Microglia_SHAM_vs_Microglia_OGD	0.102765037	0.190416884
Fdx2	1.84E-08	0.0003977	Microglia_SHAM_vs_Microglia_OGD	0.216940802	0.33418078
Sgpl1	1.80E-08	0.0003882	Microglia_SHAM_vs_Microglia_OGD	1.01391248	0.770310386
Mtmr4	1.80E-08	0.0003873	Microglia_SHAM_vs_Microglia_OGD	0.040750661	0.099164411
Zdhhc12	1.78E-08	0.0003839	Microglia_SHAM_vs_Microglia_OGD	0.195828996	0.29699588
Hdac2	1.74E-08	0.0003758	Microglia_SHAM_vs_Microglia_OGD	0.474749635	0.605665766
Thap11	1.74E-08	0.0003746	Microglia_SHAM_vs_Microglia_OGD	0.087937814	0.155956033
Lrrfip1	1.73E-08	0.0003729	Microglia_SHAM_vs_Microglia_OGD	1.379817016	1.011834093
Rpp25l	1.73E-08	0.0003719	Microglia_SHAM_vs_Microglia_OGD	0.208565504	0.321597653
Osbpl11	1.72E-08	0.0003699	Microglia_SHAM_vs_Microglia_OGD	0.278847081	0.387322354
Rce1	1.64E-08	0.0003533	Microglia_SHAM_vs_Microglia_OGD	0.286513229	0.399463579
Hist1h2an	1.63E-08	0.0003517	Microglia_SHAM_vs_Microglia_OGD	0.210776294	0.3345846
Ptp4a1	1.62E-08	0.0003503	Microglia_SHAM_vs_Microglia_OGD	0.334588735	0.456053928
Dnajc16	1.62E-08	0.0003501	Microglia_SHAM_vs_Microglia_OGD	0.091619335	0.17306284
Uchl3.1	1.60E-08	0.0003455	Microglia_SHAM_vs_Microglia_OGD	0.147794268	0.237228789
Hsf1	1.60E-08	0.0003441	Microglia_SHAM_vs_Microglia_OGD	0.099484627	0.172510074
Astn1	1.59E-08	0.0003435	Microglia_SHAM_vs_Microglia_OGD	0.393085627	0.248807006
Aph1b	1.56E-08	0.0003358	Microglia_SHAM_vs_Microglia_OGD	0.814307818	1.059493526
Hnrnpf	1.55E-08	0.0003331	Microglia_SHAM_vs_Microglia_OGD	3.302288906	3.799787739
Atp2b1	1.52E-08	0.0003275	Microglia_SHAM_vs_Microglia_OGD	4.50978843	3.636968331
Plxnb2	1.51E-08	0.0003257	Microglia_SHAM_vs_Microglia_OGD	0.74437218	0.942430207
Map4k4	1.51E-08	0.0003249	Microglia_SHAM_vs_Microglia_OGD	3.957035439	3.102529413
Tom1l1	1.48E-08	0.00032	Microglia_SHAM_vs_Microglia_OGD	0.276366361	0.403634306
Def6	1.48E-08	0.0003199	Microglia_SHAM_vs_Microglia_OGD	0.043917695	0.098591736
Hbb-bs	1.48E-08	0.0003185	Microglia_SHAM_vs_Microglia_OGD	0.001926984	0.045898095
Osgin1	1.47E-08	0.0003164	Microglia_SHAM_vs_Microglia_OGD	0.055970546	0.142209958
Tanc2	1.45E-08	0.0003119	Microglia_SHAM_vs_Microglia_OGD	0.949923024	1.114424387
Slc48a1	1.44E-08	0.0003095	Microglia_SHAM_vs_Microglia_OGD	0.435992617	0.605427848
Mbp	1.43E-08	0.0003072	Microglia_SHAM_vs_Microglia_OGD	4.816928605	5.275309643
Usp45	1.42E-08	0.0003069	Microglia_SHAM_vs_Microglia_OGD	0.13107936	0.216303282
Trappc5	1.41E-08	0.0003046	Microglia_SHAM_vs_Microglia_OGD	0.255685987	0.392591799
Gcdh	1.40E-08	0.0003013	Microglia_SHAM_vs_Microglia_OGD	0.047629494	0.11237023
Top1	1.33E-08	0.0002857	Microglia_SHAM_vs_Microglia_OGD	4.790718442	4.080705879

Rab30	1.32E-08	0.0002835	Microglia_SHAM_vs_Microglia_OGD	0.088979112	0.176747223
Csnk1a1	1.30E-08	0.0002808	Microglia_SHAM_vs_Microglia_OGD	3.922657151	3.22633706
Milr1	1.29E-08	0.0002782	Microglia_SHAM_vs_Microglia_OGD	0.190540812	0.282147462
Senp6	1.28E-08	0.0002755	Microglia_SHAM_vs_Microglia_OGD	1.545562794	1.808511573
Akap9	1.26E-08	0.0002721	Microglia_SHAM_vs_Microglia_OGD	0.987582278	1.182619491
Chd4	1.25E-08	0.0002702	Microglia_SHAM_vs_Microglia_OGD	4.317860192	3.637519587
Fam114a1	1.25E-08	0.0002685	Microglia_SHAM_vs_Microglia_OGD	0.108016716	0.180557945
Ngly1	1.23E-08	0.0002653	Microglia_SHAM_vs_Microglia_OGD	0.203482126	0.293801474
Slc7a1	1.22E-08	0.0002636	Microglia_SHAM_vs_Microglia_OGD	0.729048013	0.502357389
Slc66a2	1.22E-08	0.0002627	Microglia_SHAM_vs_Microglia_OGD	0.27295936	0.399888685
Tmed10	1.21E-08	0.000261	Microglia_SHAM_vs_Microglia_OGD	1.490740935	1.807647427
Tmc7	1.21E-08	0.0002603	Microglia_SHAM_vs_Microglia_OGD	0.06985524	0.144654664
Pgam1	1.20E-08	0.0002594	Microglia_SHAM_vs_Microglia_OGD	1.087003051	1.33331838
Etf1	1.19E-08	0.000257	Microglia_SHAM_vs_Microglia_OGD	1.814342299	2.083331922
Clptm1	1.17E-08	0.0002514	Microglia_SHAM_vs_Microglia_OGD	0.541560176	0.739302451
Stard4	1.16E-08	0.0002501	Microglia_SHAM_vs_Microglia_OGD	0.026770593	0.083205993
Rbck1	1.15E-08	0.0002479	Microglia_SHAM_vs_Microglia_OGD	0.188554384	0.288731425
Gtf2e2	1.12E-08	0.0002413	Microglia_SHAM_vs_Microglia_OGD	0.143668047	0.236087999
Adss	1.11E-08	0.0002387	Microglia_SHAM_vs_Microglia_OGD	1.372099409	1.09045466
Cttnbp2nl	1.09E-08	0.0002339	Microglia_SHAM_vs_Microglia_OGD	8.932293815	9.830088441
Rnaseh2b	1.08E-08	0.0002333	Microglia_SHAM_vs_Microglia_OGD	0.289335443	0.411863067
Bhlhe40	1.08E-08	0.0002326	Microglia_SHAM_vs_Microglia_OGD	0.479388336	0.287364132
P4hb	1.08E-08	0.0002323	Microglia_SHAM_vs_Microglia_OGD	3.039591458	2.459135772
Dhrs7b	1.06E-08	0.0002286	Microglia_SHAM_vs_Microglia_OGD	0.17936847	0.294652843
Ppm1m	1.06E-08	0.000228	Microglia_SHAM_vs_Microglia_OGD	0.096913778	0.174724488
Nmt2	1.01E-08	0.0002173	Microglia_SHAM_vs_Microglia_OGD	0.497839999	0.318106765
Pcdh17	1.00E-08	0.0002162	Microglia_SHAM_vs_Microglia_OGD	0.057239029	0.009468107
Slc7a5	9.72E-09	0.0002097	Microglia_SHAM_vs_Microglia_OGD	0.731822529	0.484176429
Sec22a	9.69E-09	0.000209	Microglia_SHAM_vs_Microglia_OGD	0.274607941	0.412011611
Tmem223	9.64E-09	0.0002078	Microglia_SHAM_vs_Microglia_OGD	0.275078342	0.407238381
Cdc123	9.56E-09	0.0002061	Microglia_SHAM_vs_Microglia_OGD	0.153859133	0.261465412
Slc39a3	9.30E-09	0.0002005	Microglia_SHAM_vs_Microglia_OGD	0.06963179	0.146122247
Yipf4	9.29E-09	0.0002004	Microglia_SHAM_vs_Microglia_OGD	0.351271962	0.469307606
Maged2	9.20E-09	0.0001984	Microglia_SHAM_vs_Microglia_OGD	0.172740232	0.242901544
C3	9.14E-09	0.0001972	Microglia_SHAM_vs_Microglia_OGD	1.563641178	1.909457251
Bcl7b	9.13E-09	0.0001968	Microglia_SHAM_vs_Microglia_OGD	0.355509456	0.468641864
Anapc5	8.87E-09	0.0001912	Microglia_SHAM_vs_Microglia_OGD	0.309003705	0.439691861
Cep350	8.43E-09	0.0001819	Microglia_SHAM_vs_Microglia_OGD	0.330310019	0.458774827
Phf20	8.42E-09	0.0001815	Microglia_SHAM_vs_Microglia_OGD	0.438948853	0.599030119
RGD1562114	8.31E-09	0.0001791	Microglia_SHAM_vs_Microglia_OGD	0.199144147	0.312812261
Lcp2	8.18E-09	0.0001763	Microglia_SHAM_vs_Microglia_OGD	1.707785784	2.014835079
Skap2	8.05E-09	0.0001736	Microglia_SHAM_vs_Microglia_OGD	1.353765206	1.593036993
Pafah1b1	7.97E-09	0.0001717	Microglia_SHAM_vs_Microglia_OGD	3.954527585	4.525926799
Folr1	7.94E-09	0.0001711	Microglia_SHAM_vs_Microglia_OGD	0.010988809	0.059689554
Ddx17	7.89E-09	0.0001702	Microglia_SHAM_vs_Microglia_OGD	0.997292809	1.231924823
Txnl4a	7.88E-09	0.00017	Microglia_SHAM_vs_Microglia_OGD	0.405045192	0.548466774
Vim	7.74E-09	0.0001668	Microglia_SHAM_vs_Microglia_OGD	1.036820548	1.318303615
Lsm7	7.69E-09	0.0001657	Microglia_SHAM_vs_Microglia_OGD	0.666166643	0.84196107
Zfp771	7.65E-09	0.000165	Microglia_SHAM_vs_Microglia_OGD	0.0443327	0.093271933
Zc3h13	7.55E-09	0.0001629	Microglia_SHAM_vs_Microglia_OGD	1.760794441	1.322432749
Pgm2	7.42E-09	0.0001601	Microglia_SHAM_vs_Microglia_OGD	0.149944391	0.263358693
Lpin2	7.41E-09	0.0001599	Microglia_SHAM_vs_Microglia_OGD	0.38806883	0.524636802
Fis1	7.37E-09	0.0001589	Microglia_SHAM_vs_Microglia_OGD	1.51689029	1.816366308
Tmem165	7.18E-09	0.0001549	Microglia_SHAM_vs_Microglia_OGD	0.308024716	0.409092295
Ythdc1	7.18E-09	0.0001549	Microglia_SHAM_vs_Microglia_OGD	2.245877106	1.796115304
Tbl1x	6.99E-09	0.0001507	Microglia_SHAM_vs_Microglia_OGD	0.460262451	0.608192455
Orc5	6.91E-09	0.000149	Microglia_SHAM_vs_Microglia_OGD	0.076712103	0.150813228
Tiparp	6.89E-09	0.0001486	Microglia_SHAM_vs_Microglia_OGD	0.861102425	0.578471722
Tomm22	6.77E-09	0.000146	Microglia_SHAM_vs_Microglia_OGD	0.711080889	0.916227775
Siah1	6.75E-09	0.0001455	Microglia_SHAM_vs_Microglia_OGD	0.232884685	0.353146227
Ostc	6.52E-09	0.0001406	Microglia_SHAM_vs_Microglia_OGD	0.552901318	0.699527716
Cbx1	6.43E-09	0.0001385	Microglia_SHAM_vs_Microglia_OGD	0.189347905	0.276935387
Peli1	6.36E-09	0.0001372	Microglia_SHAM_vs_Microglia_OGD	2.046023752	1.622731504
Cobll1	6.34E-09	0.0001366	Microglia_SHAM_vs_Microglia_OGD	0.070044577	0.023725462
Ube2j1	6.30E-09	0.0001357	Microglia_SHAM_vs_Microglia_OGD	0.493415113	0.643149368
Uqcrh	6.24E-09	0.0001346	Microglia_SHAM_vs_Microglia_OGD	2.936637374	2.447590121
Nuak1	5.93E-09	0.0001279	Microglia_SHAM_vs_Microglia_OGD	0.727344738	0.935153221
Prdx5	5.91E-09	0.0001274	Microglia_SHAM_vs_Microglia_OGD	1.044433799	0.716061743
Tpd52l2	5.87E-09	0.0001266	Microglia_SHAM_vs_Microglia_OGD	0.174388728	0.298702735
Sec11c	5.84E-09	0.000126	Microglia_SHAM_vs_Microglia_OGD	1.107289642	1.352442147
Glo1	5.83E-09	0.0001257	Microglia_SHAM_vs_Microglia_OGD	1.918613237	2.306233694
Sgta	5.71E-09	0.0001232	Microglia_SHAM_vs_Microglia_OGD	0.247464373	0.37197838
Dstn	5.60E-09	0.0001208	Microglia_SHAM_vs_Microglia_OGD	3.549107972	3.073732973
Stard3	5.43E-09	0.000117	Microglia_SHAM_vs_Microglia_OGD	0.508096812	0.657300216
Nadk	5.41E-09	0.0001167	Microglia_SHAM_vs_Microglia_OGD	0.486562242	0.651645436
Ranbp2	5.39E-09	0.0001161	Microglia_SHAM_vs_Microglia_OGD	1.465668444	1.140761531
Ripk3	5.35E-09	0.0001154	Microglia_SHAM_vs_Microglia_OGD	0.446560231	0.296254465
Uqcc2	5.32E-09	0.0001147	Microglia_SHAM_vs_Microglia_OGD	0.240610982	0.393754867
Smyd2	5.31E-09	0.0001145	Microglia_SHAM_vs_Microglia_OGD	0.130561467	0.213886006
Retreg2	5.26E-09	0.0001134	Microglia_SHAM_vs_Microglia_OGD	0.057757035	0.131494706
Abcd1	5.21E-09	0.0001124	Microglia_SHAM_vs_Microglia_OGD	0.060210996	0.115374795
Uap1	5.18E-09	0.0001116	Microglia_SHAM_vs_Microglia_OGD	0.456790223	0.26649707
Ncor1	5.16E-09	0.0001113	Microglia_SHAM_vs_Microglia_OGD	1.629661001	1.963177271
Arf5	5.07E-09	0.0001093	Microglia_SHAM_vs_Microglia_OGD	1.850761116	2.119029761
Klrb1b	5.06E-09	0.0001092	Microglia_SHAM_vs_Microglia_OGD	0.167903498	0.08106324
Chic2	4.99E-09	0.0001076	Microglia_SHAM_vs_Microglia_OGD	0.613981058	0.779222244

Commd2	4.89E-09	0.0001053	Microglia_SHAM_vs_Microglia_OGD	0.371441332	0.496051283
Uqcr10	4.88E-09	0.0001052	Microglia_SHAM_vs_Microglia_OGD	2.233545442	2.662987213
Araf	4.87E-09	0.000105	Microglia_SHAM_vs_Microglia_OGD	0.179959623	0.286913291
Tm9sf2	4.87E-09	0.0001049	Microglia_SHAM_vs_Microglia_OGD	0.448738246	0.571989307
Fance	4.79E-09	0.0001032	Microglia_SHAM_vs_Microglia_OGD	0.106457313	0.17756167
Zfp655	4.74E-09	0.0001022	Microglia_SHAM_vs_Microglia_OGD	0.376313578	0.239318288
Klhl6	4.70E-09	0.0001014	Microglia_SHAM_vs_Microglia_OGD	1.621871915	1.191966906
Bicd2	4.70E-09	0.0001012	Microglia_SHAM_vs_Microglia_OGD	0.216231365	0.336401546
Dusp8	4.67E-09	0.0001006	Microglia_SHAM_vs_Microglia_OGD	0.058684216	0.130647894
Atf6	4.66E-09	0.0001004	Microglia_SHAM_vs_Microglia_OGD	1.630233544	1.231845587
